# Genetic evidence strengthens the bidirectional connection between gut microbiota and periodontitis: insights from a two-sample Mendelian randomization study

**DOI:** 10.1186/s12967-023-04559-9

**Published:** 2023-09-28

**Authors:** Xinjian Ye, Bin Liu, Yijing Bai, Yue Cao, Sirui Lin, Linshuoshuo Lyu, Haohao Meng, Yuwei Dai, Ding Ye, Weiyi Pan, Zhiyong Wang, Yingying Mao, Qianming Chen

**Affiliations:** 1 School of Stomatology, Zhejiang University School of Medicine, Zhejiang Provincial Clinical Research Center for Oral Diseases, Key Laboratory of Oral Biomedical Research of Zhejiang Province, Stomatology Hospital, Cancer Center of Zhejiang University, Hangzhou, China; 2 Department of Epidemiology, School of Public Health, Zhejiang Chinese Medical University, Hangzhou, China; 3 The First School of Clinical Medicine, Zhejiang Chinese Medical University, Hangzhou, China; 4 School of Stomatology, Zhejiang Chinese Medical University, Hangzhou, China; 5 Department of Environmental Health Sciences, Yale School of Public Health, New Haven, USA

**Keywords:** Gut microbiota, Periodontitis, Mendelian randomization, Oral-gut axis, Extra-oral inflammatory comorbidity, Probiotics

## Abstract

**Background:**

Recent research has established the correlation between gut microbiota and periodontitis via oral-gut axis. Intestinal dysbiosis may play a pivotal bridging role in extra-oral inflammatory comorbidities caused by periodontitis. However, it is unclear whether the link is merely correlative or orchestrated by causative mechanistic interactions. This two-sample Mendelian randomization (MR) study was performed to evaluate the potential bidirectional causal relationships between gut microbiota and periodontitis.

**Materials and Methods:**

A two-sample MR analysis was performed using summary statistics from genome-wide association studies (GWAS) for gut microbiota (n = 18,340) and periodontitis (cases = 12,251; controls = 22,845). The inverse-variance weighted (IVW) method was used for the primary analysis, and we employed sensitivity analyses to assess the robustness of the main results. The PhenoScanner database was then searched for pleiotropy SNPs associated with potential confounders. In order to identify the possibly influential SNPs, we further conducted the leave-one-out analysis. Finally, a reverse MR analysis was performed to evaluate the possibility of links between periodontitis and genetically predicted gut microbiota alternation.

**Results:**

2,699 single nucleotide polymorphisms (SNPs) associated with 196 microbiota genera were selected as instrumental variables (IVs). IVW method suggested that order *Enterobacteriales* (OR: 1.35, 95% CI 1.10–1.66), family *Bacteroidales* S24.7group (OR: 1.22, 95% CI 1.05–1.41), genus *Lachnospiraceae* UCG008 (OR: 1.16, 95% CI 1.03–1.31), genus *Prevotella* 7 (OR: 1.11, 95% CI 1.01–1.23), and order *Pasteurellales* (OR: 1.12, 95% CI 1.00–1.26) may be associated with a higher risk of periodontitis, while genus *Ruminiclostridium* 6 may be linked to a lower risk (OR: 0.82, 95% CI 0.70–0.95). The sensitivity and heterogeneity analyses yielded no indication of horizontal pleiotropy or heterogeneity. Only the association between order *Enterobacteriales* and the likelihood of periodontitis remained consistent across all alternative MR approaches. In the reverse MR analysis, four microbiota genera were genetically predicted to be down-regulated in periodontitis, whereas two were predicted to be up-regulated.

**Conclusions:**

The present MR analysis demonstrated the potential bidirectional causal relationships between gut microbiota and periodontitis. Our research provided fresh insights for the prevention and management of periodontitis. Future research is required to support the finding of our current study.

**Supplementary Information:**

The online version contains supplementary material available at 10.1186/s12967-023-04559-9.

## Introduction

Triggered by etiological agents and contributory factors, periodontitis is a chronic infectious disease of the periodontal supporting tissues [1]. Severe periodontitis, as a major public health issue, threatens thousands of people worldwide, imposing a considerable economic and health burden on society [2]. Gut microbiota is the largest microbial habitat in the human body, and since it performs crucial metabolic and immunological functions, any changes in it may have substantial systemic repercussions [3].

Recent studies have highlighted the ‘‘oral-gut axis’’ in the interactions between oral and gut microbiota [4, 5], which may be also involved in the crosstalk of periodontitis-mediated systemic inflammatory comorbidities [6]. Microbial dysregulation and immunological inflammatory responses induced by ‘‘oral-gut axis’’ alterations are common manifestations of periodontitis and multiple inflammatory comorbidities [7]. Specifically, periodontitis-associated pathobionts may influence the composition of intestinal microbiota by continuous saliva swallowing, hence impacting systemic diseases [8, 9]. Systemic disease-induced changes in gut microbiota, on the other hand, are frequently accompanied by changes in oral microbiota and local periodontal lesions via affecting the host immune response [10, 11].

Observational studies have revealed a relationship between gut microbiota and periodontitis in recent years, with modifications in intestine microbiota species observed in ligature-induced periodontitis mice and periodontitis patients [12, 13]. Besides, by addressing the imbalance of oral and gut microbiota, periodontal treatment has been shown to successfully reduce inflammatory symptoms in patients suffering from periodontitis and systemic disorders [14]. Some non-surgical periodontal therapy (NSPT) methods, such as oral probiotics, have been proposed as adjuncts in subgingival instrumentation to adjust the ecology of gut environmental niches, in an effort to maintain the intestinal micro-ecological balance and reverse the established dysbiosis [15, 16]. These preliminary studies revealed the significance of gut microbiota in periodontitis, despite the fact that there was minimal clinical evidence to support them [17].

Nonetheless, from a medical and therapeutic standpoint, it is significant to determine whether the link between gut microbiota and periodontitis is purely correlative or driven by causative mechanistic interactions. Despite extensive research into epidemiology and pathophysiology, the causal association between gut microbiota and periodontitis remains unclear due to reverse causality and other confounding effects [18]. Mendelian randomization (MR) leverages the disease-genotype correlation to simulate the effect of exposure factors on disease by introducing genetic variations related to exposure factors as instrumental variables (IVs) [19]. With advantages of temporal rationality and minimization of confounding factors, MR is viewed as a complementary strategy to randomized controlled trials [20].

Here, we conducted a two-sample bidirectional MR study based on the publicly available genome-wide association studies (GWAS) databases to investigate the potential causal relationships between gut microbiota and periodontitis, providing genetic evidence for the significance of intestine flora in periodontitis.

## Methods

### Study design

**Fig. 1:**
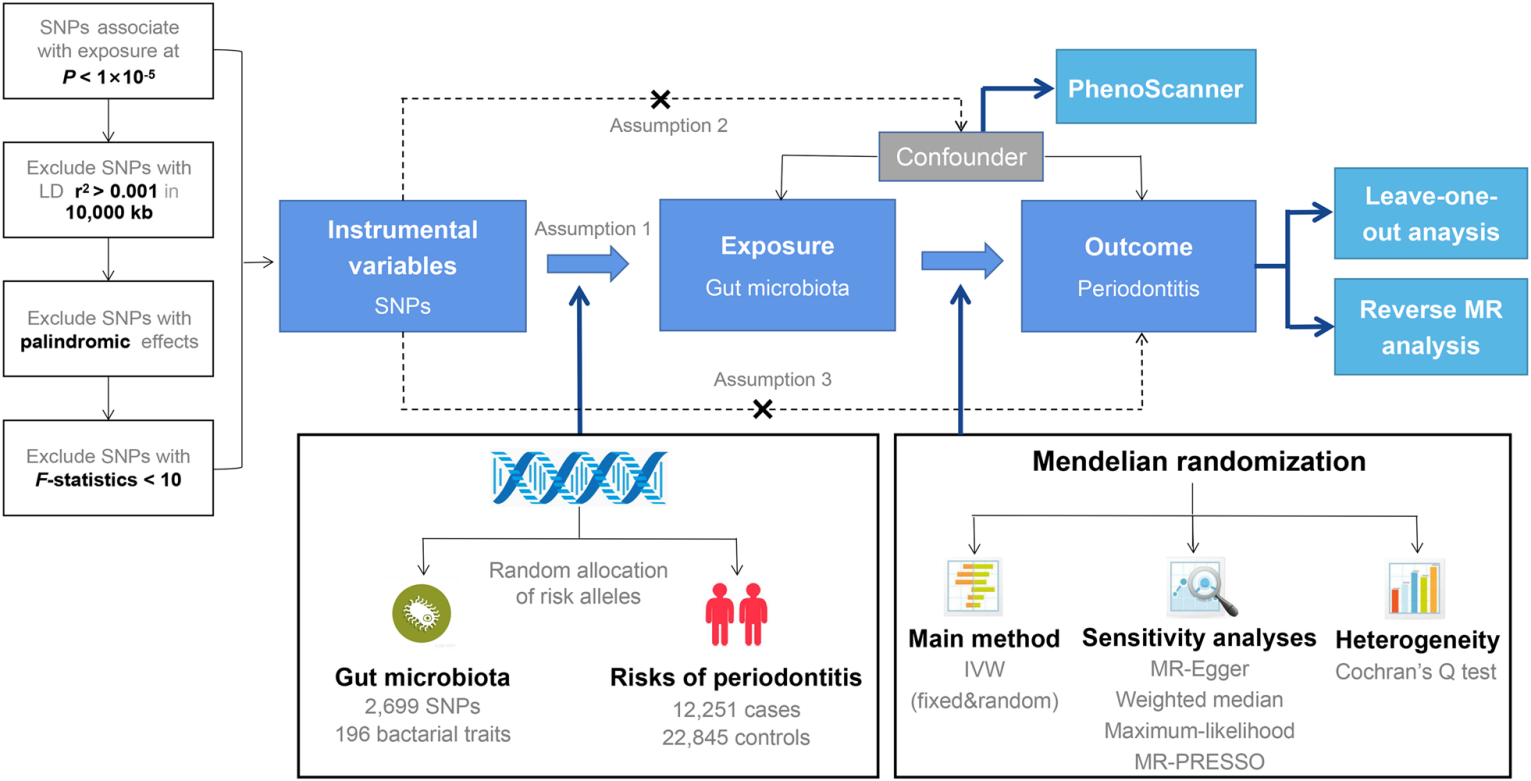
The core design and key assumptions of the present MR study. *IVW* inverse-variance weighted, the main analysis to investigate the association between exposure and outcome, *LD* linkage disequilibrium, it is used to calculate the correlations between SNPs; *MR* Mendelian randomization, *SNP* single nucleotide polymorphism, as genetic instrumental variables for the exposure and outcome, *MR-PRESSO* Mendelian randomization pleiotropy RESidual Sum and Outlier, a method for testing and correcting pleiotropic biases in SNPs

In our study, single nucleotide polymorphisms (SNPs) from GWAS were selected as genetic IVs. As presented in Fig. 1, our two-sample MR study was built upon three principal assumptions [21]:

1. Relevance assumption: The IVs had a strong connection to the exposure.
2. Independence assumption: There was no correlation between the IVs and any variables that affected both exposure and outcome.
3. Exclusion restriction assumption: The IVs did not alter the outcome through any other causal pathways other than their effects on the exposure.

No further ethical approval was required because the present study was based on publicly available GWAS data. Our study was reported according to the “STrengthening the Reporting of OBservational studies in Epidemiology using Mendelian Randomization (STROBE-MR)” checklist [22].

### Data source

A meta-analysis of GWAS, which comprised 18,340 individuals from 24 mixed-descent cohorts, yielded summary statistics for human gut microbiota composition [23]. After adjusting for age, gender, technical variables, and genetic principal components, association estimates for 211 bacterial taxa were obtained using both genetic and gut microbiota data.

Periodontitis summary statistics were derived from the Gene-Lifestyle Interactions in Dental Endpoints consortium (GLIDE) [24], which included six European ancestry cohorts (cases = 12,251; controls = 22,845). Among them, three were diagnosed using the Centers for Disease Control and Prevention/American Academy of Periodontology (CDC/AAP) criterion, two were diagnosed through the Community Periodontal Index (CPI), and one was participant-reported periodontitis.

Table 1 and Additional file 1: Tables S1, S2 highlight the features of GWAS characteristics and included cohorts.

**Table 1:** Description of GWAS information

Traits	Year	Cohorts	Population			SNPs	PMID
			Number	Age	Decent		
Gut microbiota	2021	24	18,340	4–88	Mixed	5,717,754	33462485
Periodontitis	2019	6	35,096	18–93	European	10,800,407	31235808

*GWAS* genome-wide association study, *SNP* single nucleotide polymorphism

### Instrument selection

The gut microbiota was categorized into 5 biological groupings after the removal of 15 bacterial taxa without specific name (unknown family or genus). We first selected IVs for gut microbiota based on a loose cutoff at *P* < 1 × 10^–5^ [25, 26]. Independent SNPs (*r*^2^ < 0.001, distance > 10,000 kb) were preserved after calculating the linkage disequilibrium (LD) of related SNPs. Palindromic SNPs, whose alleles consist of a base and its complementary base, were also excluded due to their confusing targeted alleles. In the reverse MR analysis, independent SNPs with genome-wide significance (*P* < 5 × 10^–6^ and *r*^2^ < 0.001, distance > 10,000 kb) were selected as IVs for periodontitis.

The detailed information on the included IVs is summarized in Additional file 1: Table S3.

### Statistical analyses

First, *R*^2^ was introduced to denote the proportion of phenotypic variance interpreted by SNPs (Eq. 1) [27]. *F*-statistics were further calculated cumulatively in order to evaluate the strength of IVs (Eq. 2) [28]. The threshold of *F*-statistic > 10 was considered as strong statistical power, indicating the weak instrument bias was unlikely to impact the effect estimates of the causal linkages [29].

1 $${R}^{2}=2\times EAF\times \left(1-EAF\right)\times {Beta}^{2}$$

2 $$F-\mathrm{statistic}=\frac{\mathrm{n}-\mathrm{k}-1}{\mathrm{k}}\times \frac{{R}^{2}}{1-{R}^{2}}$$

(Note: n, k, and EAF indicate the sample size, the number of IVs used, and effect allele frequency, respectively).

The primary study employed the inverse-variance weighted (IVW) approach, which assumed the validity of all IVs and combined the effects to produce a weighted total effect [30]. To measure the heterogeneity of IVs, Cochran's Q statistics were used. If significant heterogeneity was discovered (*P* < 0.05), the random-effects model was applied. Otherwise, the fixed-effects model was applied (*P* > 0.05) [31]. We further conducted a series of sensitivity analyses to assess the robustness of the results from IVW. When the effect of sensitivity analyses was identical to that of IVW with *p*-value < 0.05, the results were considered stable. Firstly, the weighted median estimator was used to produce robust causal estimates when even up to 50% IVs were invalid [32]. Secondly, under an assumption of a linear relationship between exposure and outcome, the maximum likelihood-based method offered normal bivariate distribution for the estimated causal association [33]. Thirdly, to give more robust causal conclusions, the MR pleiotropy residual sum and outlier (MR-PRESSO) test was used to detect and correct outliers with potential horizontal pleiotropy by deleting aberrant SNPs [26]. Fourthly, the MR-Egger technique included an intercept term in the regression model to quantify the directional pleiotropy. An intercept term that was considerably different from zero in statistics revealed the presence of pleiotropy and a breach of the basic MR assumption [34].

Moreover, we searched the PhenoScanner database for previously published confounders related to included SNPs with genome-wide significance (*P* < 1 × 10^−5^) to explore and minimize interferences from potential confounding factors, as well as to ensure the stability of the results [35]. The leave-one-out analysis was also employed to identify the influential SNPs in the causal estimates between significant gut microbiota and periodontitis [36]. Finally, a reverse MR analysis was performed to assess the possibility of reverse causality between genetically predicted gut microbiota alternation and periodontitis.

*P* < 0.05 for two-sided was regarded as the threshold of statistical significance. Odds ratios (OR) with 95% confidence intervals (CI) were used to describe the effect between gut microbiota and periodontitis. All analyses were performed using “MendelianRandomization (version 0.7.0)”, “MRPRESSO (version 1.0)”, and “TwoSampleMR (version 0.5.7)” packages in R software (version 4.3.1), as well as Sangerbox [37].

## Results

### Selection of instrumental variables

Following a variety of quality control procedures, 2699 SNPs associated with 196 bacterial species were selected as IVs. The *F*-statistics for gut microbiota ranged from 21.63 to 144.84, with an average of 52.04, all of which exceeded the threshold of > 10, indicating that weak instrument bias was less likely to occur. It should be noted that the more taxonomically distinct microbiota genera were picked when two of them shared the same SNPs in our study (e.g., we used the order *Enterobacteriales* other than the family *Enterobacteriaceae*).

### Causal effects of gut microbiota on periodontitis

**Fig. 2:**
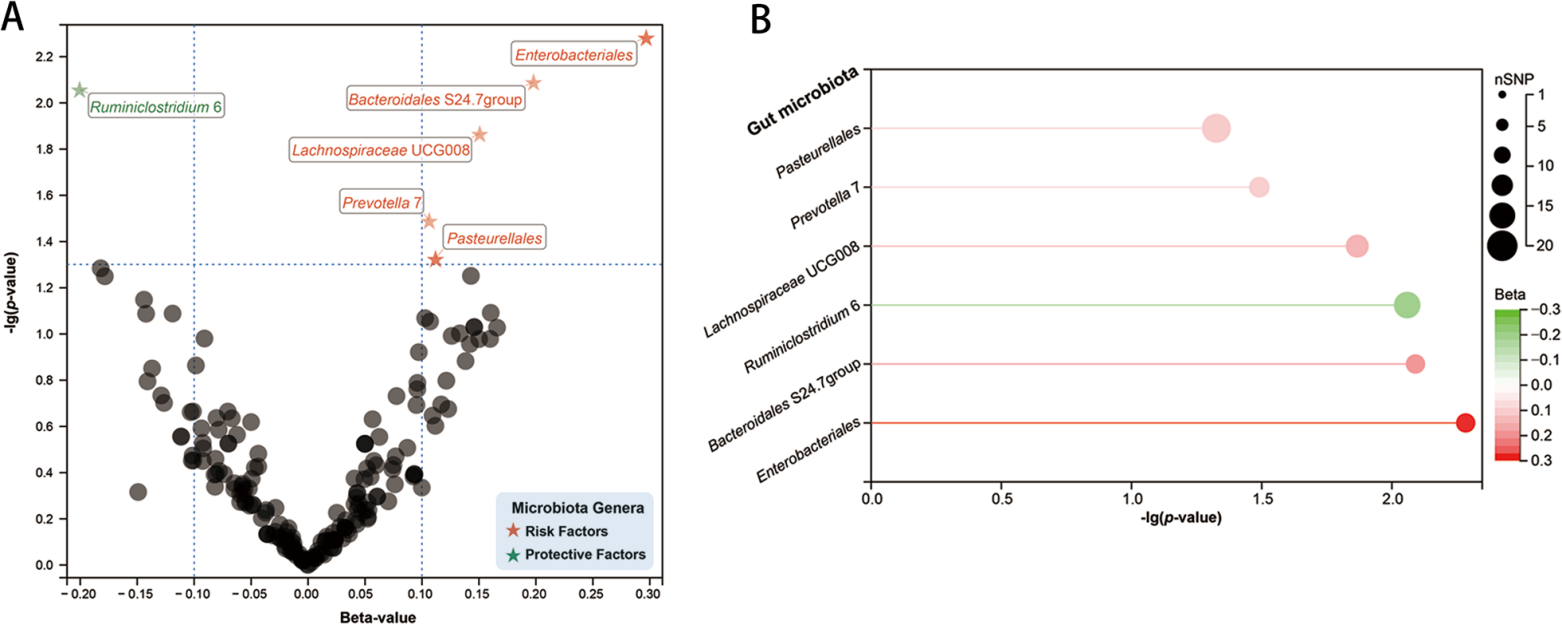
Results of the primary IVW analysis. **A** The volcano plot illustrates the link between 196 gut microbiota and periodontitis risk. The X-axis represents the beta-value, the Y-axis represents the logarithmic *p*-value with a base of 10, *P* < 0.05 is considered as statistically significant. Red and green star points represent the risk and protective microbiota genera for periodontitis, respectively. **B** The lollipop plot further depicts six statistically significant gut microbiota genera by *p*-value rank, the size of the points represents the number of SNPs, and the color of the points represents the beta-value. *CI* confidence interval, *IVW* inverse-variance weighted, *OR* odds ratio; *SNP* single nucleotide polymorphism

**Fig. 3:**
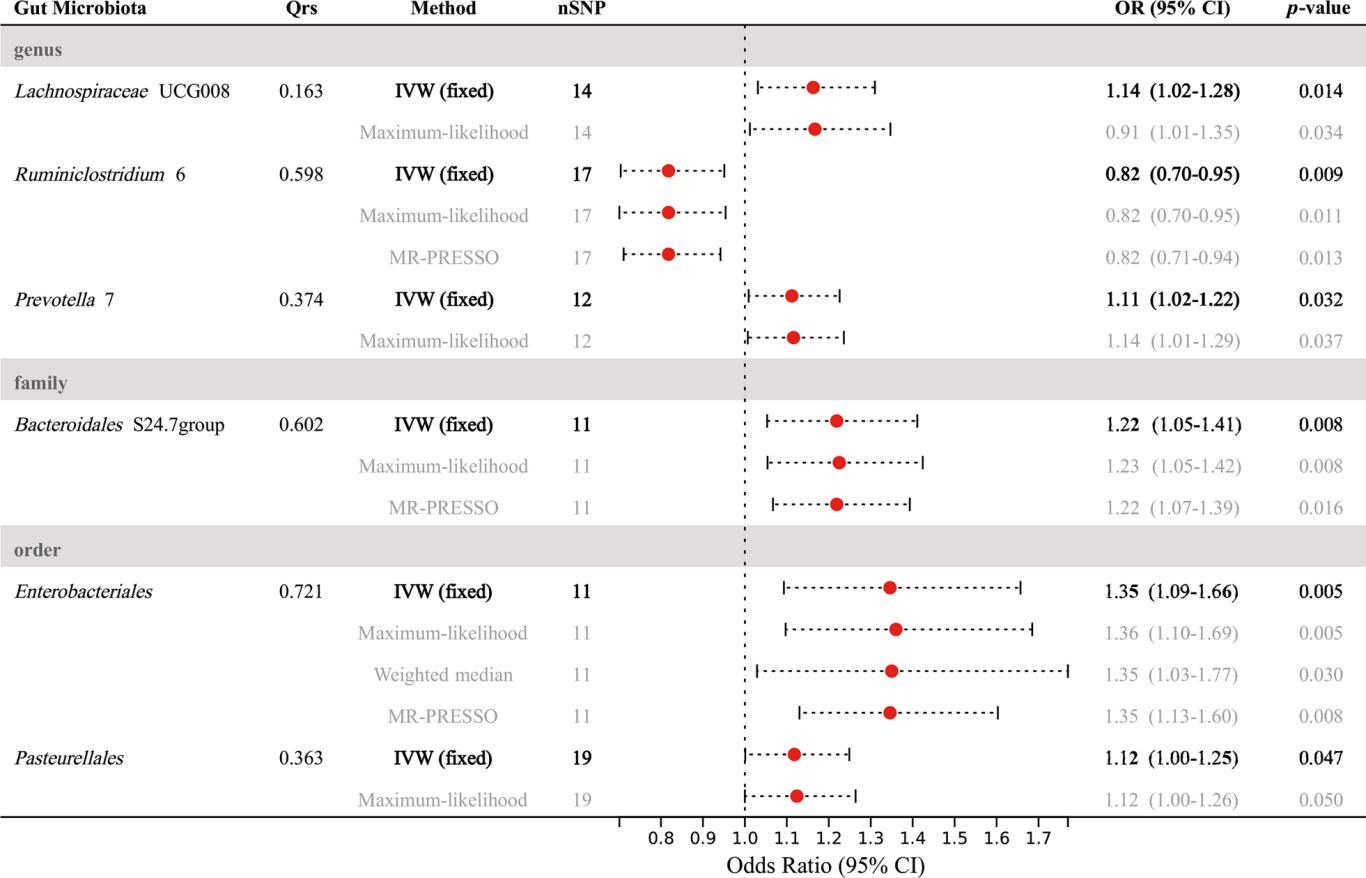
Forest plot of the MR analyses for the associations between gut microbiota genera and risk of periodontitis. *CI* confidence interval, *MR* Mendelian randomization; *OR* odds ratio

In the exploration stage, we adopted the IVW method to conduct a preliminary investigation (Fig. 2). No significant heterogeneity was found through Cochran’ Q tests. As a result, we discovered that order *Enterobacteriales* (OR: 1.35, 95% CI 1.09–1.66, *P* = 0.005), family *Bacteroidales* S24.7group (OR: 1.22, 95% CI 1.05–1.41, *P* = 0.008), genus *Lachnospiraceae* UCG008 (OR: 1.16, 95% CI 1.03–1.31, *P* = 0.014), genus *Prevotella* 7 (OR: 1.11, 95% CI 1.01–1.23, *P* = 0.032), and order *Pasteurellales* (OR: 1.12, 95% CI 1.00–1.26, *P* = 0.047), were linked to a higher risk of periodontitis, while genus *Ruminiclostridium* 6 was linked to a lower risk of periodontitis (OR: 0.82, 95% CI 0.70–0.95, *P* = 0.009) (Fig. 3).

In terms of sensitivity analysis, MR-Egger regression analysis revealed no signs of directional pleiotropy (*p*-value for intercept term > 0.05). In the maximum-likelihood method, all microbiota genera remained stable, while three of them remained stable in the MR-PRESSO test (OR: 1.35, 95% CI 1.13–1.60, *P* = 0.008 for order *Enterobacteriales*; OR: 0.82, 95% CI 0.71–0.94, *P* = 0.013 for genus *Ruminiclostridium* 6; OR: 1.22, 95% CI 1.07–1.39, *P* = 0.016 for family *Bacteroidales* S24.7group). In the weighted-median method, however, only the order *Enterobacteriales* remained stable (OR: 1.35, 95% CI 1.03–1.77, *P* = 0.03) (Fig. 3 and Additional file 1: Table S4).

**Fig. 4:**
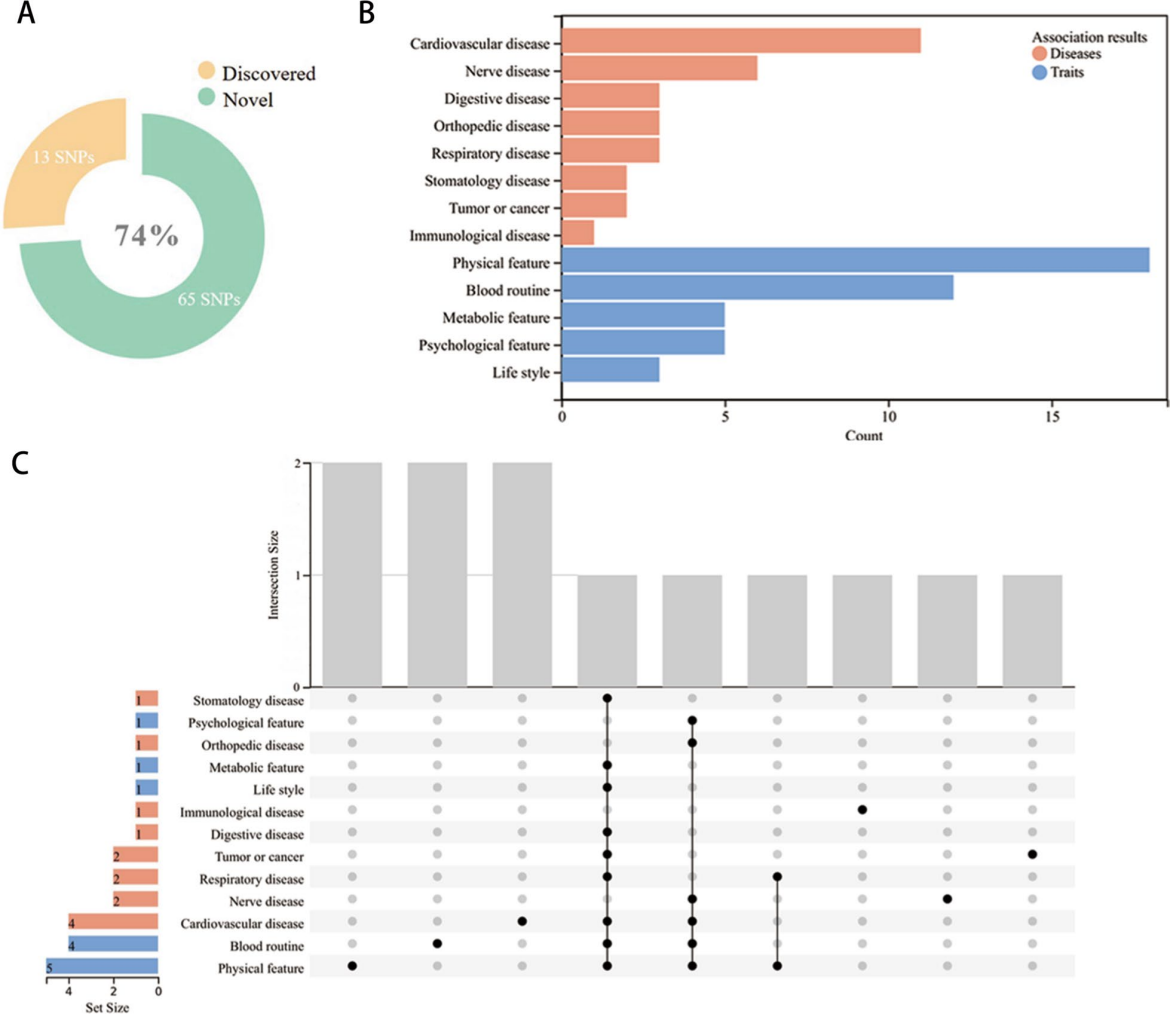
Results of sensitivity analyses for potential confounders. **A** The PhenoScanner database was examined for previously recognized confounders associated with SNPs of genome-wide significance (*P* < 1 × 10 ^−5^), the results demonstrate that 74% of the SNPs are novel in the database. **B** The bar chart displays the types and counts of diseases and traits related with the included SNPs. **C** The UpSet Venn diagram depicts the link between the included SNPs as well as the data sets of diseases and traits. *SNP* single nucleotide polymorphism

Moreover, based on the search results of PhenoScanner database (Additional file 1: Table S5), novel SNPs accounted for 74% of the IVs in our study. Eight diseases and five traits in the research results were identified as potential confounding factors. And the primary confounders were regarded as physical feature, blood routine and cardiovascular disease (Fig. 4). Of note, *rs2548459* has been linked to dentition defect and edentulism. However, considering the modest connections between *rs2548459* and periodontitis, this pleiotropy should be minimal. After removing these pleiotropic SNPs, four microbiota genera still maintained statistically significant, validating the results of the present MR study (Additional file 2: Fig. S1). Last but not least, the leave-one-out analysis discovered that there were no influential SNPs that were substantially associated with the outcome (Additional file 1: Fig. S2).

### Causal effects of periodontitis on gut microbiota

**Fig. 5:**
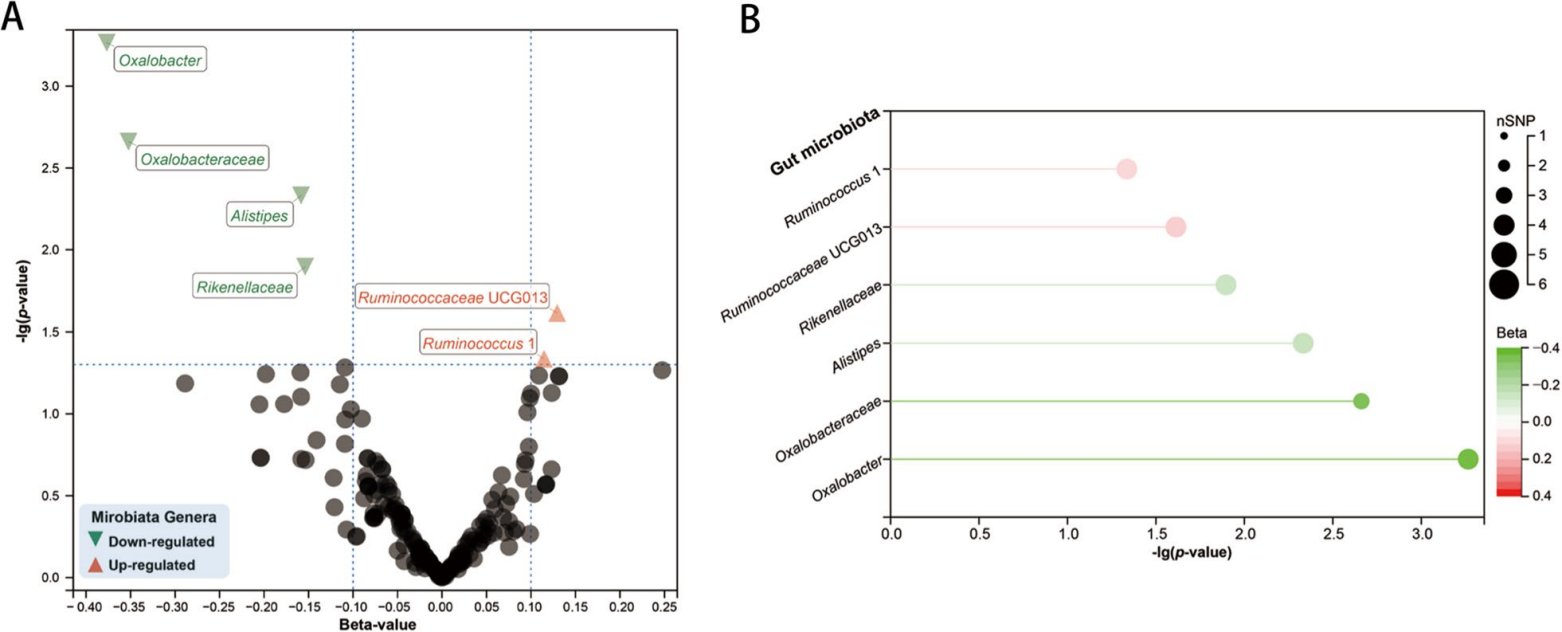
Results of the reverse MR analysis. **A** The volcano plot illustrates the effect of periodontitis on genetically predicted composition of 196 gut microbiota. The X-axis represents the beta-value, the Y-axis represents the logarithmic *p*-value with a base of 10, *P* < 0.05 is considered as statistically significant. Red and green triangle points represent the microbial genera up and down regulated by periodontitis, respectively. **B** The lollipop plot further depicts six statistically significant intestinal microbial genera by *p*-value rank, the size of the points represents the number of SNPs, and the color of the points represents the beta-value. *IVW* inverse-variance weighted, *SNP* single nucleotide polymorphism

In the reverse direction, four SNPs linked to periodontitis met the criteria for usage as IVs. There was no evidence of weak instrument bias or heterogeneity statistics among the IVs, nor of horizontal pleiotropy between IVs and microbiota genera. According to IVW analysis, 59.2% of the genetically predicted microbiota genera showed a down-regulated trend in periodontitis, with four microbiota genera demonstrating a statistically significant decline (OR: 1.22, 95% CI 1.05–1.41, *P* = 0.008 for genus *Oxalobacter*; OR: 0.70, 95% CI 0.56–0.88, *P* = 0.002 for family *Oxalobacteraceae*; OR: 0.85, 95% CI 0.77–0.95, *P* = 0.005 for genus *Alistipes*; and OR: 0.86, 95% CI 0.76–0.97, *P* = 0.013 for family *Rikenellaceae*). Furthermore, two microbiota genera, including genus *Ruminococcaceae* UCG013 (OR: 1.14, 95% CI 1.02–1.27, *P* = 0.024) and genus *Ruminococcus* 1 (OR: 1.12, 95% CI 1.00–1.26, *P* = 0.046), exhibited a statistically significant increase in periodontitis (Fig. 5). In sensitivity analysis, four down-regulated microbiota genera remained stable (Additional file 1: Table S6 and Additional file 2: Fig. S3).

## Discussion

In the present research, we employed a two-sample MR study to explore the causal potential relationship between gut microbiota genera and periodontitis. We uncovered signs that the gut microbiota genera and periodontitis may interact. Specifically, five microbiota genera were discovered to be associated with a higher risk of periodontitis, whereas one was discovered to be linked with a lower risk. Besides, periodontitis affected the genetically predicted composition of gut microbiota genera, with statistically significant differences reported in six of these genera.

The relationship between gut microbiota and periodontitis has long piqued the interest of researchers [38]. Traditional research methods, however, are unable to fully explain the complex relationships between gut microbiota and periodontitis due to confounding variables and temporal causal interactions [19]. Exploring from the standpoint of host genetic variation becomes an attractive and crucial research field [39]. A recent study in the TwinsUK registry, for example, confirmed the connections between a collection of putative host genetic variants and gut microbial composition by periodontal condition [40]. Similar benefits applied to the research of MR, the development of MR provides a new paradigm for studying causal linkages, which has been widely applied in the field of periodontitis (Additional file 1: Table S7).

Notably, the order *Enterobacteriales*, which remained stable among 196 microbiota genera in all sensitivity analyses, may play key roles in periodontitis. Previous research discovered that *Enterobacteriales*, as one of the most prevalent bacteria in the intestine, can flourish in an inflammatory environment due to metabolic alterations [41]. As a result, mass proliferation of *Enterobacteriales* may jeopardize colonization resistances mediated by the indigenous microbiota, leading to increased inflammatory sensitivity [42]. Consistent with our findings, Kitamoto observed that *Enterobacteriales* accumulate in both the oral cavity and the intestine as a result of periodontitis [43]. Interestingly, the accumulated *Enterobacteriales* in the intestine may be translocated and ectopically colonized by periodontitis-induced oral microbiota, indicating the interaction and linkage between gut and oral microbiota [6].

We also revealed that the gut microbiota *Lachnospiraceae* UCG008, *Prevotella* 7, *Bacteroidales* S24.7group, *Pasteurellales*, and *Ruminiclostridium* 6 were causally associated with periodontitis, shedding light on the role of the gut microbiota in periodontal etiology. Similar to our findings, a study discovered *Ruminococcaceae* and *Prevotella* in greater abundance in the intestines of periodontitis patients, while *Lactobacillales* and *Prevotella* were detected in higher proportions in the intestines of gingivitis patients [13]. *Prevotella* was also detected four times more frequently in the subgingival microbiome of adults with severe periodontitis than in periodontally healthy people according to a recent research [44]. Intriguingly, the microbiota found to be related with periodontitis in our study closely mirrored the microbiome found to be associated with anxiety disorders in a research by Wei [45], implying that gut microbiota may mediate periodontitis-systemic disease comorbidity.

In the reverse MR, we explored the effect of periodontitis on the genetically predicted gut microbiota alternation. Given that the majority of the bacteria in our database were intestine-resident, it was not surprising that the majority of the microbiota genera in our research showed a down-regulated pattern during periodontitis, which may reflect the harm caused by periodontitis to the healthy gut microbiota. A clinical study revealed that periodontitis patients had a decrease in the α-variety of gut microbiota, as seen by a rise in the *Firmicutes* to *Bacteroidetes* ratio [13]. Similar modifications were found in our study with an increase in *Firmicutes* (genus *Ruminococcaceae* UCG013 and *Ruminococcus* 1) and a decrease in *Bacteroidetes* (genus *Alistipes*). We further observed changes in genus *Oxalobacter*, family *Oxalobacteraceae*, and family *Rikenellaceae* in an ingenious way. Unfortunately, we were unable to find specific periodontal pathogens such as *Porphyromonas gingivalis* and *Fusobacterium nucleatum* [46] in the database we used, undermining the genetic evidence for ectopic colonization of periodontal-derived bacteria in the intestine.

Recently, potential mechanisms of intestinal bacteria mediating oral disease and overall health has been investigated [47]. On the one hand, ectopic colonization of periodontitis-associated pathobionts in the intestine induces intestinal inflammation and alters local homeostasis by activating both innate (e.g., macrophages) and adaptive (e.g., T helper-17 cells) immunity heterotopically via the “oral-gut axis” [6, 7], which was also identified as a key link in the extra-oral comorbidity crosstalk, including inflammatory bowel disease (IBD) [48], Alzheimer's disease (AD) [49], nonalcoholic fatty liver disease (NAFLD) [50], colorectal cancer [51], hypertension [4], and arthritis [5]. Changes in the variety and quantity of intestinal microbiota induced by systemic disease, on the other hand, frequently coexist with extraintestinal symptoms in locations such as the oral cavity, which manifested as more severe loss of periodontal attachment and alveolar bone resorption in people with periodontitis [11]. These symptoms were thought to be related to an inflammatory sensitive state and an aberrant host immune response triggered by a breakdown in gut flora equilibrium [48, 52]. A recent study observed that trimethylamine-N-oxide (TMAO) can regulate periodontal immunology and inflammation by changing the intestinal milieu, which may influence periodontitis development via the bidirectional interaction of the “oral-gut axis” [53]. The importance of gut microbiota in periodontitis and general health indicates that we can explore targets on the “oral-gut axis” to manage and intervene in inflammation disorders by governing intestinal microbiota using immunological approaches [7].

Finally, our findings have several clinical implications. Brownlie’s study found that probiotics containing *lactobacilli* acids inhibited the growth of commensal *Lachnospiraceae* and *Bacteroidales* S24.7group bacteria [54], while, in our study, these two microbiota genera were identified to be associated with a higher risk of periodontitis. We also discovered that order *Ruminiclostridium* 6, a Gram-positive probiotic, may reduce the likelihood of periodontitis. The present findings made it reasonable to figure out that probiotics featuring specific microbial genera (e.g., *lactobacill* or *Ruminiclostridium*) may play a role in periodontitis. As suggested by a systematic review [55], the appropriate use of probiotics as adjuncts to subgingival instrumentation may be beneficial to the management of periodontitis, as well as to the prevention or mitigation of extra-oral inflammatory comorbidities [56]. Despite the fact that the European Federation of Periodontology (EFP) has not yet supported this application due to a lack of relevant data on its efficacy [57].

However, there were certain limitations in our study that should be addressed when interpreting the results. First of all, while we strive for uniformity throughout population sources, a small part of the gut microbiota data was obtained from multiple race sets, which may have biased our estimates. Second, due to the limited information available in the GWAS database, bacterial taxa were only analyzed at the genus level rather than at more specialized levels (e.g., species or strains). Similarly, we only covered periodontitis and were unavailable to undertake further subtype analysis (e.g., gingivitis or periodontal abscess). Third, several sensitivity analyses disputed significant results from the primary IVW method. We were unable to entirely rule out interferences from unobserved pleiotropies despite our best efforts to explore and eliminate confounding factors. Fourth, we did not locate any representative IVs when employed the standard IVs criteria (*P* < 5 × 10^–8^), thus we used a more flexible threshold during the screening process (*P* < 1 × 10^–5^ for gut microbiota, *P* < 5 × 10^–6^ for periodontitis). Due to the same reasons, we failed to correct the results using multiple testing correction. Fifth, the conclusions may not be entirely applicable to people of non-European ancestry, and the use of summary-level statistics may result in the omission of critical information. Finally, even though we adhered to the STROBE-MR statement, not all of its recommendations could be met for the restricted availability of information (e.g., we were unable to determine whether overlapping individuals were enrolled between the exposure and outcome).

## Conclusions

The present MR analysis confirmed the bidirectional causal relationships between gut microbiota and periodontitis. Our research offered some supports for the prevention and management of periodontitis as well as fresh information on the mechanisms underlying periodontal-systemic comorbidities caused by gut microbiota. Future research is required to back up the findings of our current study.

### Supplementary Information

**Additional file1: Table S1.** Characteristics of the cohorts included in the genome-wide meta-analysis of periodontitis. **Table S2.** Characteristics of the cohorts included in the genome-wide meta-analysis of gut microbiome. **Table S3.** Characteristics of the genetic variants associated with gut microbiome and periodontitis that have been identified statistically significant. **Table S4.** Effect estimates of the associations between 196 bacterial traits and risk of periodontitis in MR analysis. **Table S5.** The results of relevant confounding factors for included SNPs obtained from the PhenoScanner database. **Table S6.** Effect estimates of the associations between periodontitis and genetically predicted gut microbiome traits in the reverse MR analysis. **Table S7.** Summary of MR Studies related to periodontitis.

**Additional file 2: Fig. S1.** Forest plot of the results after removing potential pleiotropic SNPs. **Fig.** **S2.** Results of leave-one-out sensitivity analysis. **Fig. S3.** Forest plot of the results in reverse MR for the causal effects of periodontitis on genetically predicted gut microbiota composition.

## Data Availability

Data from GLIDE can be obtained via application (https://data.bris.ac.uk/data/dataset/). Data about potential confounding factors can be searched in the PhenoScanner database via application (https://www.phenoscanner.medschl.cam.ac.uk/). The data generated or analyzed during this study are available in this published article and its supplementary information files.

## Abbreviations

AD: Alzheimer’s disease
CDC/AAP: Centers for Disease Control and Prevention/American Academy of Periodontology
CI: Confidence interval
CPI: Community periodontal index
EAF: Effect allele frequency
EFP: European federation of periodontology
GLIDE: Gene-lifestyle interactions in dental endpoints
GWAS: Genome-wide association studies
IBD: Inflammatory bowel disease
IVs: Instrumental variables
IVW: Inverse-variance weighted
LD: Linkage disequilibrium
MR: Mendelian randomization
MR-PRESSO: MR pleiotropy residual sum and outlier test
NAFLD: Nonalcoholic fatty liver disease
NSPT: Non-surgical periodontal therapy
OR: Odds ratio
SNPs: Single nucleotide polymorphisms
STROBE-MR: STrengthening the reporting of OBservational studies in epidemiology using Mendelian randomization
TMAO: Trimethylamine-N-oxide
