# Supplementary material for: Genetic evidence strengthens the bidirectional connection between gut microbiota and periodontitis: insights from a two-sample Mendelian randomization study
Source: J Transl Med. 2023 Sep 28;21:674. doi: 10.1186/s12967-023-04559-9 (PMC10537583; doi:10.1186/s12967-023-04559-9)
Supplement: Supplementary file 1 — Additional file1: Table S1. Characteristics of the cohorts included in the genome-wide meta-analysis of periodontitis. Table S2. Characteristics of the cohorts included in the genome-wide meta-analysis of gut microbiome. Table S3. Characteristics of the genetic variants associated with gut microbiome and periodontitis that have been identified statistically significant. Table S4. Effect estimates of the associations between 196 bacterial traits and risk of periodontitis in MR analysis. Table S5. The results of relevant confounding factors for included SNPs obtained from the PhenoScanner database. Table S6. Effect estimates of the associations between periodontitis and genetically predicted gut microbiome traits in the reverse MR analysis. Table S7. Summary of MR Studies related to periodontitis. [file 12967_2023_4559_MOESM1_ESM.docx]

Supporting information for

**Genetic evidence strengthens the bidirectional connection between gut microbiota and periodontitis: insights from a two-sample Mendelian randomization study**

*Xinjian Ye, Bin Liu, Yijing Bai, Yue Cao, Sirui Lin, Linshuoshuo Lyu, Haohao Meng, Yuwei Dai, Ding Ye, Weiyi Pan, Zhiyong Wang, Yingying Mao & Qianming Chen*

**Additional file 1: Supplementary Tables**

Table of Contents

[Table S1. Characteristics of the cohorts included in the genome-wide meta-analysis of periodontitis. 2](#_Toc26331)

[Table S2. Characteristics of the cohorts included in the genome-wide meta-analysis of gut microbiome. 4](#_Toc10189)

[Table S3. Characteristics of the genetic variants associated with gut microbiome and periodontitis that have been identified statistically significant. 10](#_Toc17976)

[Table S4. Effect estimates of the associations between 196 bacterial traits and risk of periodontitis in MR analysis. 13](#_Toc8871)

[Table S5. The results of relevant confounding factors for included SNPs obtained from the PhenoScanner database. 46](#_Toc5115)

[Table S6. Effect estimates of the associations between periodontitis and genetically predicted gut microbiome traits in the reverse MR analysis. 50](#_Toc25355)

[Table S7. Summary of MR Studies related to periodontitis. 83](#_Toc10212)

# Table S1. Characteristics of the cohorts included in the genome-wide meta-analysis of periodontitis.

| **Cohort** | **Ethical approval** | **Participants** | | |
| --- | --- | --- | --- | --- |
|  |  | **Number** | **Age, mean (SD)** | **Female, %** |
| Atherosclerosis Risk in Communities | The institutional review board at the University of North Carolina, NC | 4,504 (2,680 / 1,824) | 62.7 (5.6) | 52.0 |
| Study of Health in Pomerania | The Local Ethics committee of the University of Greifswald, Germany | 3,264 (1,812 / 1,452) | 45.8 (14.9) | 51.1 |
| Study of Health in Pomerania Trend | The Local Ethics committee of the University of Greifswald, Germany | 819 (424 / 395) | 49.2 (13.2) | 55.1 |
| The Center for Oral Health in Appalachia cohort 1 | The University of Pittsburgh Institutional Review Board, PA, site approval, West Virginia University Institutional Review Board, WV | 772 (161 / 611) | 33.9 (9.0) | 62.7 |
| Swedish Twin Biobank | The Local ethics committee at Karolinska Institute, Sweden | 2,849 (1,521 / 1,328) | 63.6 (8.0) | 53.6 |
| Women's Genome Health Study | The Brigham and Women’s Hospital Institutional Review Board for Human Subjects Research | 22,888 (5,653 / 17,235) | 54.7 (7.1) | 100.0 |

*(Continued* ***Table S1****)*

| **Cohort** | **Design** | | | | **PMID** |
| --- | --- | --- | --- | --- | --- |
|  | **Recruitment age** | **Period** | **Recruitment strategy** | **Diagnostic criteria** |  |
| Atherosclerosis Risk in Communities | 45-64 | 1987-89, 1990-92, 1993-95, 1996-98 | A random selection from the defined populations were invited to participate | Centers for Disease Control and Prevention/American Academy of Periodontology definitions | 11701471, 23459936, 26962152 |
| Study of Health in Pomerania | 20-81 | 1997-2001 | Two-stage cluster sampling design by sex and age |  | 25496455, 24024966 |
| Study of Health in Pomerania Trend | 20-83 | 2008-2012 | A stratified random sample from population registries |  | 25496455, 24024966 |
| The Center for Oral Health in Appalachia cohort 1 | 18+ | 2002-2009 | Recruited by announcements in the target regions | Probing depth ≥ 5.5 mm in 2 or more sextants, or if participants reported ever having gum surgery | 18522740, 24347629 |
| Swedish Twin Biobank | 46-93 | 2004-2008 | All twins born before 1958 who were registered in the Swedish Twin Register | Two or more tooth surfaces with probing depth ≥ 5 mm, or at least four tooth surfaces with probing depth ≥ 4 mm | 17254424, 23137839, 26544805 |
| Women's Genome Health Study | 45+ | 1992-1995 | Recruited 70.6% of all women in Women's Health Study | Participant-reported diagnosis of periodontitis | 25385537, 29928831 |

**Abbreviation**: *SD*, standard deviation

# Table S2. Characteristics of the cohorts included in the genome-wide meta-analysis of gut microbiome.

| **Cohort** | **Ethical approval** | **Participants** | | | | | |
| --- | --- | --- | --- | --- | --- | --- | --- |
|  |  | **Ethnicity** | **Number** | **Age, mean (SD)** | **Female, %** | **PPI users** | **Antibiotic users** |
| BSPSPC | The institutional ethical review committee of Kiel University, Germany | European | 721 | 61.5 (12.6) | 45.1 | NA | NA |
| Coronary Artery Risk Development in Young Adults Study Microbiome-B | Institutional Review Boards of University of Alabama at Birmingham, Birmingham, AL, Kaiser Permanente Division of Research, Oakland CA | Afro-American | 114 | 54.3 (3.8) | 59.6 | 15 | 16 |
| Coronary Artery Risk Development in Young Adults Study Microbiome-W |  | European | 257 | 55.8 (3.2) | 52.3 | 17 | 37 |
| The Danish study of Functional Disorders | Ethical Committee of Copenhagen County (Ethics Committee: KA-2006-0011; H-3-2011-081; H-3-2012-0015) | European | 2396 | 54.9 (11.5) | 53.0 | NA | NA |
| Flemish Gut Flora Project | The medical ethics committee of the University of Brussels; Brussels University Hospital | European | 2259 | 50.9 (14.3) | 59.7 | 359 | 625 |
| FOCUS | The institutional ethical review committee of Kiel University, Germany | European | 960 | 51.4 (14.6) | 58.0 | NA | NA |
| The CCC GEM-HCE_v12 | Mount Sinai Hospital Research Ethics Board (Toronto-Managing Center) and local centers | European | 378 | 18.6 (7.8) | 54.8 | NA | 24 |
| The CCC GEM-HCE_v24 |  | Admixed | 203 | 17.5 (8.1) | 51.7 | NA | 26 |
| The CCC GEM-ICHIP_HCE |  | European | 662 | 20.9 (7.9) | 55.1 | NA | 23 |
| The Generation R Study | The Medical Ethical Committee of Erasmus MC, University Medical Center Rotterdam | Multi-ethnic | 1328 | 9.8 (0.3) | 49.4 | 0 | 0 |
| Kangbuk Samsung Cohort Study | The EUMC review board 2014-06-024 and KBSMC review board 2013-01-245 | East Asian | 811 | 44.1 (8.1) | 39.3 | 13 | 28 |
| HCHS/SOL | NA | Hispanic | 1097 | 57.2 (10.9) | 61.6 | 341 | 321 |
| LifeLines-DEEP | The UMCG Institutional Review Board | European | 875 | 45.1(13.3) | 57.6 | 70 | 8 |
| METabolic Syndrome In Men | Ethics Committee of the Northern Savo Hospital District, Finland | European | 522 | 61.9(5.4) | 0.0 | 0 | 4 |
| Maastricht Irritable Bowel Syndrome | The Maastricht University Medical Center, IRB | European | 80 | 48.7(13.2) | 57.5 | NA | NA |
| NeurGenetics Research Consortium | The institutional review boards of the participating institutions: Albany Medical Center, Emory University; Kaiser Permanente Northwest Division | European | 77 | 71.9(7.5) | 58.4 | 11 | 12 |
| Personalized Nutrition Project | Tel Aviv Sourasky Medical Center Institutional Review Board: TLV-0658-12, TLV-0050-13 and TLV-0522-10; Kfar Shaul Hospital IRB: 0-73; Weizmann Institute of Science Bioethics and Embryonic Stem Cell Research oversight committee | Middle-East | 481 | 43.7 (13.1) | 63.6 | NA | NA |
| Population-based Colonoscopy | The local Committee of Research Ethics (Forskningskommitté Syd) at Karolinska Institute, Stockholm | European | 134 | 54.8(11.3) | 61.9 | 6 | 12 |
| Rotterdam Study III | The IRB of the Erasmus Medical Center and by the review board of the Netherlands Ministry of Health, Welfare and Sports | European | 1220 | 62.3(5.9) | 57.8 | 260 | 0 |
| Study of Health in Pomerania | The medical ethics committee of the University of Greifswald | European | 996 | 56.9 (13.5) | 53.6 | 98 | 19 |
| Study of Health in Pomerania -TREND |  | European | 905 | 50.2 (13.7) | 56.2 | 51 | 6 |
| COPSAC2010 | Danish Ethics Committee (H-B-2008-093) and the Danish Data Protection Agency (2008-41-2599) | European | 380 | 4.4 (0.8) | 47.1 | 0 | 84 |
| The Netherlands Twin Registry | Central Ethics Committee on Research involving human subjects of the VU University Medical Center, Amsterdam | European | 279 | 35.4(11.7) | 70.3 | NA | NA |
| TwinsUK | The Cornell University IRB | European | 1205 | 61.5(10.7) | 91.4 | 62 | 78 |

*(Continued* ***Table S2****)*

| **Cohort** | **Design** | | | | **PMID** |
| --- | --- | --- | --- | --- | --- |
|  | **Recruitment age** | **Recruitment period** | **Recruitment strategy** | **Study design** |  |
| BSPSPC | 25-82 | NA | NA | Population-based | NA |
| Coronary Artery Risk Development in Young Adults Study Microbiome-B | 48-61 | NA | NA |  | NA |
| Coronary Artery Risk Development in Young Adults Study Microbiome-W |  |  |  |  |  |
| The Danish study of Functional Disorders | 18-76 | 2011-2015 | Recruited men and women from the general population examined randomly |  | 28275316 |
| Flemish Gut Flora Project | 16-88 | 2013.6-2016.4 | NA |  | 27126039 |
| FOCUS | 16-81 | NA | NA |  | NA |
| The CCC GEM-HCE_v12 | 6-35 | since 2008 | Recruited healthy first-degree relatives of Crohn’s Disease patients |  | NA |
| The CCC GEM-HCE_v24 |  |  |  |  |  |
| The CCC GEM-ICHIP_HCE |  |  |  |  |  |
| The Generation R Study | 0-13 | From the early prenatal phase to early adolescence | Recruited children born between April 2002 and January 2006 |  | 28070760 |
| Kangbuk Samsung Cohort Study | NA | 2014.6-2014.9 | NA |  | NA |
| HCHS/SOL | NA | NA | NA |  | NA |
| LifeLines-DEEP | 18-81 | 2013.4-2013.8 | NA |  | NA |
| METabolic Syndrome In Men | 45-73 | 2005-2010 | Recruited by randomly selected non-diabetic Finnish men |  | NA |
| Maastricht Irritable Bowel Syndrome | 19-71 | NA | Recruited age and gender matched healthy controls of a clinical diagnosis of IBS according to the Rome III criteria |  | NA |
| NeurGenetics Research Consortium | 55-88 | 2009-2014 | Recruited controls who were free of neurodegenerative disease |  | 20711177 |
| Personalized Nutrition Project | 18-70 | Personalized Nutrition Project | NA |  | NA |
| Population-based Colonoscopy | 18-70 | NA | Recruited by randomly selected |  | 20205503, 24384686 |
| Rotterdam Study III | ≥40 | 2006-2012 | Recruited adult/elderly individuals aged ≥40 years living in the Rotterdam, Netherlands |  | 29064009 |
| Study of Health in Pomerania | 20-79 | 2008-2012 | From the total population of West Pomerania inhabitants in 1996, and stratification variables are age, sex and city/county of residence. |  | 20167617 |
| Study of Health in Pomerania-TREND |  |  |  |  |  |
| COPSAC2010 | 4-6 | 2008-2010 | Seven hundred and thirty‐eight mothers were recruited from week 24 of gestation, and 700 of their children were included in the birth cohort | Children | 24118234 |
| The Netherlands Twin Registry | 19-68 | NA | One of each twin pair from the Netherlands Twin Register was randomly selected | Twins Study | 35511521 |
| TwinsUK | 18-82 | 2010-2016 | One twin out of each pair was randomly excluded from the population of volunteers |  | 31526404 |

**Abbreviation**: *SD*, standard deviation.

# Table S3. Characteristics of the genetic variants associated with gut microbiome and periodontitis that have been identified statistically significant.

| **Trait** | **SNP** | **Chr** | **Position** | **Effect allele** | **Beta** | **SE** | ***p*-value** |
| --- | --- | --- | --- | --- | --- | --- | --- |
| **Family. *Bacteroidales* S24.7group** | rs7217209 | 17 | 21205200 | C | 0.084 | 0.019 | 8.43E-06 |
|  | rs738193 | 22 | 25943322 | T | 0.085 | 0.017 | 3.82E-07 |
|  | rs689695 | 15 | 47352866 | C | 0.081 | 0.017 | 1.28E-06 |
|  | rs17043785 | 2 | 53139362 | T | -0.176 | 0.035 | 5.12E-07 |
|  | rs78609301 | 6 | 63236747 | A | -0.087 | 0.020 | 7.09E-06 |
|  | rs941000 | 7 | 90714667 | C | 0.085 | 0.016 | 3.16E-07 |
|  | rs6831034 | 4 | 149300138 | T | -0.096 | 0.021 | 6.10E-06 |
|  | rs10872669 | 6 | 151515172 | A | -0.123 | 0.028 | 9.49E-06 |
|  | rs61508842* | 3 | 158653545 | T | 0.123 | 0.027 | 7.83E-06 |
|  | rs11135366 | 5 | 164763068 | C | 0.084 | 0.018 | 8.78E-06 |
|  | rs12748533 | 1 | 242767252 | G | -0.082 | 0.017 | 2.59E-06 |
|  |  |  |  |  |  |  |  |
| **Genus. *Lachnospiraceae* UCG008** | rs10741777 | 11 | 19572557 | T | -0.097 | 0.019 | 7.69E-07 |
|  | rs57254474 | 8 | 23071054 | G | 0.089 | 0.020 | 6.92E-06 |
|  | rs75356640 | 15 | 31697284 | G | 0.137 | 0.030 | 9.83E-06 |
|  | rs10751237* | 11 | 74275056 | A | -0.082 | 0.017 | 3.17E-06 |
|  | rs10793103 | 11 | 74391775 | C | 0.097 | 0.018 | 9.35E-08 |
|  | rs11236216 | 11 | 74405061 | T | 0.084 | 0.018 | 2.35E-06 |
|  | rs57091572* | 6 | 81214803 | A | -0.110 | 0.024 | 2.86E-06 |
|  | rs955844 | 16 | 84992314 | A | 0.112 | 0.023 | 1.81E-06 |
|  | rs10801803 | 1 | 90818872 | G | -0.117 | 0.024 | 1.40E-06 |
|  | rs9873555 | 3 | 100496680 | G | -0.121 | 0.023 | 2.41E-07 |
|  | rs67078837 | 4 | 114220647 | T | -0.085 | 0.017 | 7.68E-07 |
|  | rs61944774 | 12 | 129539818 | A | 0.180 | 0.039 | 6.34E-06 |
|  | rs13024781 | 2 | 168628871 | T | -0.080 | 0.017 | 2.29E-06 |
|  | rs62277846 | 2 | 228100140 | C | 0.102 | 0.021 | 1.59E-06 |
|  |  |  |  |  |  |  |  |
| **Genus. Prevotella 7** | rs12124567* | 1 | 3343802 | A | -0.121 | 0.028 | 9.49E-06 |
|  | rs57404562* | 2 | 12101762 | C | 0.155 | 0.032 | 6.22E-07 |
|  | rs16937247 | 11 | 19943115 | G | 0.146 | 0.035 | 9.64E-06 |
|  | rs9608249* | 22 | 24613952 | A | -0.158 | 0.034 | 2.07E-06 |
|  | rs9426434 | 1 | 29736216 | T | -0.124 | 0.028 | 9.72E-06 |
|  | rs79263163 | 11 | 39821773 | A | -0.144 | 0.032 | 7.51E-06 |
|  | rs118038478 | 16 | 47237609 | A | 0.206 | 0.047 | 7.85E-06 |
|  | rs430270 | 3 | 60474379 | A | 0.139 | 0.030 | 2.87E-06 |
|  | rs9959718 | 18 | 71442664 | G | 0.133 | 0.028 | 1.90E-06 |
|  | rs12195431 | 6 | 91019654 | T | 0.197 | 0.044 | 8.73E-06 |
|  | rs2918132 | 10 | 133000320 | C | -0.115 | 0.025 | 6.42E-06 |
|  | rs2240542 | 2 | 242066314 | C | 0.121 | 0.026 | 4.84E-06 |
|  |  |  |  |  |  |  |  |
| **Genus. *Ruminiclostridium* 6** | rs792058 | 2 | 5548605 | G | 0.055 | 0.013 | 8.58E-06 |
|  | rs67479537* | 19 | 10115515 | T | 0.119 | 0.026 | 9.30E-06 |
|  | rs1756364 | 14 | 20954661 | G | 0.100 | 0.020 | 2.54E-07 |
|  | rs35362464 | 4 | 36478011 | C | 0.072 | 0.017 | 8.99E-06 |
|  | rs77193512 | 11 | 40289063 | A | 0.074 | 0.015 | 1.30E-06 |
|  | rs2548459* | 19 | 49209339 | C | 0.055 | 0.012 | 6.40E-06 |
|  | rs71414120 | 14 | 56938952 | T | 0.201 | 0.041 | 1.08E-06 |
|  | rs1871858 | 15 | 61047117 | C | -0.105 | 0.024 | 9.12E-06 |
|  | rs61060922 | 16 | 72136154 | T | 0.159 | 0.032 | 1.09E-06 |
|  | rs72991535 | 18 | 76018244 | T | 0.136 | 0.030 | 4.95E-06 |
|  | rs11992182* | 8 | 79766499 | A | 0.063 | 0.014 | 4.65E-06 |
|  | rs663262 | 11 | 86179076 | T | -0.135 | 0.031 | 3.39E-06 |
|  | rs73176030 | 7 | 101271282 | T | 0.059 | 0.013 | 7.29E-06 |
|  | rs9555756 | 13 | 111703249 | A | -0.080 | 0.018 | 7.10E-06 |
|  | rs116969552 | 10 | 128214403 | A | -0.167 | 0.038 | 9.16E-06 |
|  | rs10829821 | 10 | 132651293 | T | -0.098 | 0.022 | 3.47E-06 |
|  | rs79968172 | 1 | 240503826 | G | 0.116 | 0.024 | 1.66E-06 |
|  |  |  |  |  |  |  |  |
| **Order. *Enterobacteriales*** | rs111229068 | 11 | 2563450 | A | 0.111 | 0.024 | 3.65E-06 |
|  | rs80319214 | 2 | 4015933 | C | 0.099 | 0.022 | 6.95E-06 |
|  | rs4792380* | 17 | 13521040 | A | 0.116 | 0.026 | 9.49E-06 |
|  | rs11026530 | 11 | 22379097 | T | 0.082 | 0.019 | 9.43E-06 |
|  | rs61973590 | 14 | 27718039 | C | -0.061 | 0.013 | 8.54E-06 |
|  | rs2374342 | 2 | 42133542 | C | 0.058 | 0.013 | 4.52E-06 |
|  | rs35673018 | 16 | 54327745 | G | 0.090 | 0.020 | 7.63E-06 |
|  | rs504442 | 18 | 55145547 | T | 0.084 | 0.019 | 5.17E-06 |
|  | rs62210023 | 20 | 55340092 | A | 0.061 | 0.013 | 3.13E-06 |
|  | rs78143293 | 18 | 57672335 | A | -0.085 | 0.017 | 1.20E-06 |
|  | rs79757635 | 13 | 110840418 | C | 0.076 | 0.017 | 9.32E-06 |
|  |  |  |  |  |  |  |  |
| **Order. *Pasteurellales*** | rs731534 | 20 | 5412480 | C | -0.099 | 0.022 | 9.46E-06 |
|  | rs62568866 | 9 | 6818307 | T | -0.118 | 0.026 | 7.23E-06 |
|  | rs10840326* | 11 | 9951672 | C | -0.072 | 0.015 | 1.44E-06 |
|  | rs10965428 | 9 | 22718481 | C | -0.120 | 0.026 | 4.29E-06 |
|  | rs72756943 | 5 | 26531908 | G | 0.140 | 0.030 | 3.35E-06 |
|  | rs4822728 | 22 | 26891808 | T | 0.069 | 0.015 | 4.72E-06 |
|  | rs16970009 | 17 | 32862601 | A | 0.187 | 0.043 | 7.32E-06 |
|  | rs111582866 | 16 | 48742489 | G | -0.114 | 0.026 | 7.07E-06 |
|  | rs9382510 | 6 | 55448491 | C | -0.088 | 0.017 | 2.48E-07 |
|  | rs6092684 | 20 | 57274077 | A | 0.068 | 0.015 | 3.44E-06 |
|  | rs9895850 | 17 | 64535013 | T | -0.176 | 0.041 | 9.08E-06 |
|  | rs12050685* | 15 | 73477482 | A | -0.067 | 0.015 | 9.19E-06 |
|  | rs9938097 | 16 | 84977389 | T | -0.071 | 0.016 | 8.23E-06 |
|  | rs76022354 | 10 | 94306385 | C | 0.243 | 0.050 | 1.83E-06 |
|  | rs73139353 | 3 | 97972214 | A | -0.223 | 0.048 | 8.71E-06 |
|  | rs78909003 | 9 | 105650242 | T | -0.241 | 0.050 | 2.05E-06 |
|  | rs35510 | 12 | 115491973 | A | 0.123 | 0.026 | 4.02E-06 |
|  | rs6972479 | 7 | 116918060 | A | -0.078 | 0.018 | 7.75E-06 |
|  | rs12191680 | 6 | 129224488 | C | 0.102 | 0.020 | 3.05E-07 |
|  |  |  |  |  |  |  |  |
| **Risk of periodontitis** | rs2976950* | 8 | 8249082 | A | 0.096 | 0.020 | 7.99E-07 |
|  | rs13005050 | 2 | 52705571 | T | -0.143 | 0.031 | 3.76E-06 |
|  | rs4956201 | 4 | 109527782 | C | -0.241 | 0.047 | 3.89E-07 |
|  | rs6816769 | 4 | 122216017 | C | -0.135 | 0.029 | 4.57E-06 |

**Abbreviations:** *Chr*, chromosome; *SE*, standard error; *SNP*, single nucleotide polymorphism.

**Note:** *SNPs detected with possible pleiotropy in **Table S6**.

# Table S4. Effect estimates of the associations between 196 bacterial traits and risk of periodontitis in MR analysis.

| **Gut microbiota** | **R^2^** | ***F*-statistic** | **Method** | **nSNP** | **OR** | **95% CI** | ***p*-value** | **Qrs** |
| --- | --- | --- | --- | --- | --- | --- | --- | --- |
| **Class** |  |  |  |  |  |  |  |  |
| *Actinobacteria* | 4.64% | 38.76 |  |  |  |  |  |  |
|  |  |  | Inverse-variance weighted (fixed) | 23 | 0.99 | 0.86-1.13 | 0.856 | 0.934 |
|  |  |  | MR-Egger | 23 | / | / | 0.941* | / |
|  |  |  | Weighted median | 23 | 1.00 | 0.82-1.21 | 0.97 | / |
|  |  |  | Maximum-likelihood method | 23 | 0.99 | 0.86-1.14 | 0.862 | / |
|  |  |  | MR-PRESSO test | 23 | 0.99 | 0.89-1.10 | 0.815 | 0.932 |
| *Alphaproteobacteria* | 2.30% | 43.24 |  |  |  |  |  |  |
|  |  |  | Inverse-variance weighted (fixed) | 10 | 0.95 | 0.79-1.13 | 0.538 | 0.899 |
|  |  |  | MR-Egger | 10 | / | / | 0.745* | / |
|  |  |  | Weighted median | 10 | 0.98 | 0.78-1.24 | 0.884 | / |
|  |  |  | Maximum-likelihood method | 10 | 0.95 | 0.79-1.13 | 0.549 | / |
|  |  |  | MR-PRESSO test | 10 | 0.95 | 0.84-1.07 | 0.39 | 0.898 |
| *Bacilli* | 3.50% | 30.17 |  |  |  |  |  |  |
|  |  |  | Inverse-variance weighted (fixed) | 22 | 0.99 | 0.85-1.15 | 0.872 | 0.83 |
|  |  |  | MR-Egger | 22 | / | / | 0.565* | / |
|  |  |  | Weighted median | 22 | 0.97 | 0.79-1.18 | 0.741 | / |
|  |  |  | Maximum-likelihood method | 22 | 0.99 | 0.85-1.15 | 0.874 | / |
|  |  |  | MR-PRESSO test | 22 | 0.99 | 0.87-1.12 | 0.849 | 0.825 |
| *Bacteroidia* | 2.28% | 28.54 |  |  |  |  |  |  |
|  |  |  | Inverse-variance weighted (fixed) | 16 | 1.16 | 0.98-1.37 | 0.093 | 0.665 |
|  |  |  | MR-Egger | 16 | / | / | 0.896* | / |
|  |  |  | Weighted median | 16 | 1.16 | 0.92-1.47 | 0.203 | / |
|  |  |  | Maximum-likelihood method | 16 | 1.16 | 0.98-1.38 | 0.084 | / |
|  |  |  | MR-PRESSO test | 16 | 1.16 | 0.99-1.35 | 0.082 | 0.671 |
| *Betaproteobacteria* | 2.02% | 25.21 |  |  |  |  |  |  |
|  |  |  | Inverse-variance weighted (fixed) | 15 | 1.17 | 0.98-1.41 | 0.081 | 0.401 |
|  |  |  | MR-Egger | 15 | / | / | 0.480* | / |
|  |  |  | Weighted median | 15 | 1.13 | 0.87-1.45 | 0.366 | / |
|  |  |  | Maximum-likelihood method | 15 | 1.19 | 0.98-1.43 | 0.077 | / |
|  |  |  | MR-PRESSO test | 15 | 1.17 | 0.98-1.41 | 0.11 | 0.418 |
| *Clostridia* | 1.97% | 21.63 |  |  |  |  |  |  |
|  |  |  | Inverse-variance weighted (fixed) | 17 | 0.94 | 0.78-1.14 | 0.533 | 0.32 |
|  |  |  | MR-Egger | 17 | / | / | 0.827* | / |
|  |  |  | Weighted median | 17 | 0.97 | 0.74-1.27 | 0.83 | / |
|  |  |  | Maximum-likelihood method | 17 | 0.94 | 0.77-1.15 | 0.548 | / |
|  |  |  | MR-PRESSO test | 17 | 0.94 | 0.77-1.15 | 0.566 | 0.329 |
| *Coriobacteriia* | 2.99% | 26.93 |  |  |  |  |  |  |
|  |  |  | Inverse-variance weighted (fixed) | 21 | 1.03 | 0.88-1.21 | 0.689 | 0.746 |
|  |  |  | MR-Egger | 21 | / | / | 0.851* | / |
|  |  |  | Weighted median | 21 | 0.98 | 0.78-1.23 | 0.85 | / |
|  |  |  | Maximum-likelihood method | 21 | 1.03 | 0.88-1.22 | 0.686 | / |
|  |  |  | MR-PRESSO test | 21 | 1.03 | 0.90-1.19 | 0.655 | 0.762 |
| *Deltaproteobacteria* | 2.22% | 29.78 |  |  |  |  |  |  |
|  |  |  | Inverse-variance weighted (fixed) | 14 | 1.09 | 0.92-1.29 | 0.311 | 0.467 |
|  |  |  | MR-Egger | 14 | / | / | 0.341* | / |
|  |  |  | Weighted median | 14 | 1.18 | 0.94-1.50 | 0.16 | / |
|  |  |  | Maximum-likelihood method | 14 | 1.10 | 0.92-1.30 | 0.295 | / |
|  |  |  | MR-PRESSO test | 14 | 1.09 | 0.92-1.29 | 0.325 | 0.473 |
| *Erysipelotrichia* | 1.65% | 23.71 |  |  |  |  |  |  |
|  |  |  | Inverse-variance weighted (fixed) | 13 | 0.96 | 0.78-1.19 | 0.734 | 0.913 |
|  |  |  | MR-Egger | 13 | / | / | 0.189* | / |
|  |  |  | Weighted median | 13 | 0.94 | 0.72-1.22 | 0.63 | / |
|  |  |  | Maximum-likelihood method | 13 | 0.96 | 0.78-1.19 | 0.737 | / |
|  |  |  | MR-PRESSO test | 13 | 0.96 | 0.83-1.12 | 0.642 | 0.918 |
| *Gammaproteobacteria* | 1.39% | 28.61 |  |  |  |  |  |  |
|  |  |  | Inverse-variance weighted (fixed) | 9 | 1.10 | 0.88-1.37 | 0.414 | 0.26 |
|  |  |  | MR-Egger | 9 | / | / | 0.356* | / |
|  |  |  | Weighted median | 9 | 0.98 | 0.71-1.34 | 0.881 | / |
|  |  |  | Maximum-likelihood method | 9 | 1.10 | 0.85-1.42 | 0.464 | / |
|  |  |  | MR-PRESSO test | 9 | 1.10 | 0.86-1.41 | 0.487 | 0.272 |
| *Lentisphaeria* | 4.81% | 92.58 |  |  |  |  |  |  |
|  |  |  | Inverse-variance weighted (fixed) | 10 | 1.04 | 0.92-1.18 | 0.488 | 0.735 |
|  |  |  | MR-Egger | 10 | / | / | 0.428* | / |
|  |  |  | Weighted median | 10 | 1.04 | 0.89-1.22 | 0.622 | / |
|  |  |  | Maximum-likelihood method | 10 | 1.05 | 0.92-1.18 | 0.481 | / |
|  |  |  | MR-PRESSO test | 10 | 1.04 | 0.94-1.15 | 0.419 | 0.723 |
| *Melainabacteria* | 5.25% | 78.13 |  |  |  |  |  |  |
|  |  |  | Inverse-variance weighted (fixed) | 13 | 1.02 | 0.91-1.13 | 0.781 | 0.205 |
|  |  |  | MR-Egger | 13 | / | / | 0.323* | / |
|  |  |  | Weighted median | 13 | 1.10 | 0.94-1.29 | 0.216 | / |
|  |  |  | Maximum-likelihood method | 13 | 1.02 | 0.89-1.16 | 0.802 | / |
|  |  |  | MR-PRESSO test | 13 | 1.02 | 0.90-1.15 | 0.812 | 0.229 |
| *Methanobacteria* | 7.23% | 119.05 |  |  |  |  |  |  |
|  |  |  | Inverse-variance weighted (fixed) | 12 | 1.05 | 0.96-1.16 | 0.299 | 0.399 |
|  |  |  | MR-Egger | 12 | / | / | 0.583* | / |
|  |  |  | Weighted median | 12 | 1.06 | 0.93-1.21 | 0.358 | / |
|  |  |  | Maximum-likelihood method | 12 | 1.05 | 0.96-1.16 | 0.29 | / |
|  |  |  | MR-PRESSO test | 12 | 1.05 | 0.95-1.16 | 0.332 | 0.412 |
| *Mollicutes* | 2.60% | 40.77 |  |  |  |  |  |  |
|  |  |  | Inverse-variance weighted (fixed) | 12 | 0.95 | 0.81-1.12 | 0.549 | 0.341 |
|  |  |  | MR-Egger | 12 | / | / | 0.036* | / |
|  |  |  | Weighted median | 12 | 0.98 | 0.78-1.23 | 0.837 | / |
|  |  |  | Maximum-likelihood method | 12 | 0.95 | 0.80-1.13 | 0.574 | / |
|  |  |  | MR-PRESSO test | 12 | 0.95 | 0.81-1.13 | 0.582 | 0.354 |
| *Negativicutes* | 1.67% | 23.92 |  |  |  |  |  |  |
|  |  |  | Inverse-variance weighted (fixed) | 13 | 0.89 | 0.73-1.09 | 0.278 | 0.397 |
|  |  |  | MR-Egger | 13 | / | / | 0.086* | / |
|  |  |  | Weighted median | 13 | 0.97 | 0.74-1.26 | 0.808 | / |
|  |  |  | Maximum-likelihood method | 13 | 0.90 | 0.73-1.10 | 0.303 | / |
|  |  |  | MR-PRESSO test | 13 | 0.89 | 0.73-1.10 | 0.311 | 0.426 |
| *Verrucomicrobiae* | 2.63% | 38.04 |  |  |  |  |  |  |
|  |  |  | Inverse-variance weighted (fixed) | 13 | 1.02 | 0.87-1.21 | 0.778 | 0.33 |
|  |  |  | MR-Egger | 13 | / | / | 0.102* | / |
|  |  |  | Weighted median | 13 | 1.05 | 0.85-1.31 | 0.646 | / |
|  |  |  | Maximum-likelihood method | 13 | 1.02 | 0.86-1.22 | 0.786 | / |
|  |  |  | MR-PRESSO test | 13 | 1.02 | 0.86-1.22 | 0.795 | 0.333 |
| **Family** |  |  |  |  |  |  |  |  |
| *Acidaminococcaceae* | 1.50% | 35.00 |  |  |  |  |  |  |
|  |  |  | Inverse-variance weighted (fixed) | 8 | 0.92 | 0.74-1.14 | 0.458 | 0.03 |
|  |  |  | MR-Egger | 8 | / | / | 0.493* | / |
|  |  |  | Weighted median | 8 | 0.80 | 0.60-1.06 | 0.12 | / |
|  |  |  | Maximum-likelihood method | 8 | 0.92 | 0.66-1.29 | 0.621 | / |
|  |  |  | MR-PRESSO test | 7 | 0.92 | 0.67-1.27 | 0.634 | 0.035 |
| *Actinomycetaceae* | 1.76% | 65.52 |  |  |  |  |  |  |
|  |  |  | Inverse-variance weighted (fixed) | 5 | 1.10 | 0.88-1.37 | 0.405 | 0.897 |
|  |  |  | MR-Egger | 5 | / | / | 0.854* | / |
|  |  |  | Weighted median | 5 | 1.13 | 0.87-1.47 | 0.363 | / |
|  |  |  | Maximum-likelihood method | 5 | 1.10 | 0.88-1.37 | 0.405 | / |
|  |  |  | MR-PRESSO test | 5 | 1.10 | 0.98-1.23 | 0.184 | 0.9 |
| *Alcaligenaceae* | 2.77% | 26.11 |  |  |  |  |  |  |
|  |  |  | Inverse-variance weighted (fixed) | 20 | 1.14 | 0.98-1.34 | 0.099 | 0.527 |
|  |  |  | MR-Egger | 20 | / | / | 0.167* | / |
|  |  |  | Weighted median | 20 | 1.14 | 0.91-1.43 | 0.27 | / |
|  |  |  | Maximum-likelihood method | 20 | 1.15 | 0.98-1.35 | 0.097 | / |
|  |  |  | MR-PRESSO test | 20 | 1.14 | 0.98-1.33 | 0.106 | 0.517 |
| *Bacteroidaceae* | 1.23% | 22.84 |  |  |  |  |  |  |
|  |  |  | Inverse-variance weighted (fixed) | 12 | 1.02 | 0.82-1.27 | 0.843 | 0.382 |
|  |  |  | MR-Egger | 12 | / | / | 0.932* | / |
|  |  |  | Weighted median | 12 | 0.88 | 0.64-1.20 | 0.407 | / |
|  |  |  | Maximum-likelihood method | 12 | 1.02 | 0.81-1.29 | 0.842 | / |
|  |  |  | MR-PRESSO test | 12 | 1.02 | 0.81-1.28 | 0.851 | 0.37 |
| *BacteroidalesS24.7* | 3.06% | 57.94 |  |  |  |  |  |  |
|  |  |  | Inverse-variance weighted (fixed) | 11 | 1.22 | 1.05-1.41 | 0.008 | 0.602 |
|  |  |  | MR-Egger | 11 | / | / | 0.930* | / |
|  |  |  | Weighted median | 11 | 1.14 | 0.93-1.39 | 0.206 | / |
|  |  |  | Maximum-likelihood method | 11 | 1.23 | 1.05-1.42 | 0.008 | / |
|  |  |  | MR-PRESSO test | 11 | 1.22 | 1.07-1.39 | 0.016 | 0.644 |
| *Bifidobacteriaceae* | 4.44% | 34.00 |  |  |  |  |  |  |
|  |  |  | Inverse-variance weighted (fixed) | 25 | 0.93 | 0.82-1.06 | 0.298 | 0.953 |
|  |  |  | MR-Egger | 25 | / | / | 0.206* | / |
|  |  |  | Weighted median | 25 | 0.91 | 0.76-1.10 | 0.32 | / |
|  |  |  | Maximum-likelihood method | 25 | 0.93 | 0.82-1.07 | 0.316 | / |
|  |  |  | MR-PRESSO test | 25 | 0.93 | 0.84-1.03 | 0.181 | 0.961 |
| *Christensenellaceae* | 2.03% | 31.59 |  |  |  |  |  |  |
|  |  |  | Inverse-variance weighted (fixed) | 12 | 0.84 | 0.70-1.00 | 0.056 | 0.758 |
|  |  |  | MR-Egger | 12 | / | / | 0.557* | / |
|  |  |  | Weighted median | 12 | 0.82 | 0.65-1.04 | 0.097 | / |
|  |  |  | Maximum-likelihood method | 12 | 0.84 | 0.70-1.01 | 0.063 | / |
|  |  |  | MR-PRESSO test | 12 | 0.84 | 0.72-0.97 | 0.041 | 0.78 |
| *Clostridiaceae1* | 1.71% | 29.00 |  |  |  |  |  |  |
|  |  |  | Inverse-variance weighted (fixed) | 11 | 1.08 | 0.89-1.31 | 0.447 | 0.578 |
|  |  |  | MR-Egger | 11 | / | / | 0.033* | / |
|  |  |  | Weighted median | 11 | 1.13 | 0.87-1.47 | 0.357 | / |
|  |  |  | Maximum-likelihood method | 11 | 1.08 | 0.89-1.32 | 0.438 | / |
|  |  |  | MR-PRESSO test | 11 | 1.08 | 0.90-1.29 | 0.43 | 0.537 |
| *ClostridialesvadinBB60* | 3.73% | 41.81 |  |  |  |  |  |  |
|  |  |  | Inverse-variance weighted (fixed) | 17 | 0.96 | 0.85-1.10 | 0.578 | 0.997 |
|  |  |  | MR-Egger | 17 | / | / | 0.945* | / |
|  |  |  | Weighted median | 17 | 0.93 | 0.79-1.10 | 0.401 | / |
|  |  |  | Maximum-likelihood method | 17 | 0.96 | 0.85-1.10 | 0.577 | / |
|  |  |  | MR-PRESSO test | 17 | 0.96 | 0.90-1.03 | 0.322 | 0.999 |
| *Coriobacteriaceae* | 2.99% | 26.93 |  |  |  |  |  |  |
|  |  |  | Inverse-variance weighted (fixed) | 21 | 1.03 | 0.88-1.21 | 0.689 | 0.746 |
|  |  |  | MR-Egger | 21 | / | / | 0.851* | / |
|  |  |  | Weighted median | 21 | 0.98 | 0.78-1.23 | 0.85 | / |
|  |  |  | Maximum-likelihood method | 21 | 1.03 | 0.88-1.22 | 0.686 | / |
|  |  |  | MR-PRESSO test | 21 | 1.03 | 0.90-1.19 | 0.655 | 0.764 |
| *Defluviitaleaceae* | 3.59% | 52.53 |  |  |  |  |  |  |
|  |  |  | Inverse-variance weighted (fixed) | 13 | 1.01 | 0.88-1.16 | 0.897 | 0.183 |
|  |  |  | MR-Egger | 13 | / | / | 0.235* | / |
|  |  |  | Weighted median | 13 | 0.99 | 0.80-1.22 | 0.93 | / |
|  |  |  | Maximum-likelihood method | 13 | 1.01 | 0.85-1.20 | 0.907 | / |
|  |  |  | MR-PRESSO test | 13 | 1.01 | 0.86-1.19 | 0.913 | 0.18 |
| *Desulfovibrionaceae* | 2.00% | 31.20 |  |  |  |  |  |  |
|  |  |  | Inverse-variance weighted (fixed) | 12 | 1.06 | 0.89-1.27 | 0.506 | 0.539 |
|  |  |  | MR-Egger | 12 | / | / | 0.373* | / |
|  |  |  | Weighted median | 12 | 1.18 | 0.92-1.51 | 0.192 | / |
|  |  |  | Maximum-likelihood method | 12 | 1.07 | 0.89-1.28 | 0.494 | / |
|  |  |  | MR-PRESSO test | 12 | 1.06 | 0.90-1.26 | 0.498 | 0.559 |
| *Enterobacteriaceae* | 1.84% | 31.17 |  |  |  |  |  |  |
|  |  |  | Inverse-variance weighted (fixed) | 11 | 1.35 | 1.09-1.66 | 0.005 | 0.721 |
|  |  |  | MR-Egger | 11 | / | / | 0.657* | / |
|  |  |  | Weighted median | 11 | 1.35 | 1.03-1.77 | 0.03 | / |
|  |  |  | Maximum-likelihood method | 11 | 1.36 | 1.10-1.69 | 0.005 | / |
|  |  |  | MR-PRESSO test | 11 | 1.35 | 1.13-1.60 | 0.008 | 0.739 |
| *Erysipelotrichaceae* | 1.65% | 23.71 |  |  |  |  |  |  |
|  |  |  | Inverse-variance weighted (fixed) | 13 | 0.96 | 0.78-1.19 | 0.734 | 0.913 |
|  |  |  | MR-Egger | 13 | / | / | 0.189* | / |
|  |  |  | Weighted median | 13 | 0.94 | 0.72-1.22 | 0.63 | / |
|  |  |  | Maximum-likelihood method | 13 | 0.96 | 0.78-1.19 | 0.737 | / |
|  |  |  | MR-PRESSO test | 13 | 0.96 | 0.83-1.12 | 0.642 | 0.911 |
| *FamilyXI* | 6.97% | 137.38 |  |  |  |  |  |  |
|  |  |  | Inverse-variance weighted (fixed) | 10 | 1.00 | 0.91-1.10 | 0.938 | 0.153 |
|  |  |  | MR-Egger | 10 | / | / | 0.004* | / |
|  |  |  | Weighted median | 10 | 0.99 | 0.86-1.13 | 0.866 | / |
|  |  |  | Maximum-likelihood method | 10 | 1.00 | 0.89-1.12 | 0.949 | / |
|  |  |  | MR-PRESSO test | 10 | 1.00 | 0.89-1.12 | 0.95 | 0.161 |
| *FamilyXIII* | 2.24% | 30.01 |  |  |  |  |  |  |
|  |  |  | Inverse-variance weighted (fixed) | 14 | 1.04 | 0.84-1.27 | 0.732 | 0.54 |
|  |  |  | MR-Egger | 14 | / | / | 0.734* | / |
|  |  |  | Weighted median | 14 | 0.93 | 0.70-1.23 | 0.604 | / |
|  |  |  | Maximum-likelihood method | 14 | 1.04 | 0.84-1.28 | 0.722 | / |
|  |  |  | MR-PRESSO test | 14 | 1.04 | 0.85-1.26 | 0.726 | 0.524 |
| *Lachnospiraceae* | 2.61% | 27.33 |  |  |  |  |  |  |
|  |  |  | Inverse-variance weighted (fixed) | 18 | 0.97 | 0.83-1.15 | 0.759 | 0.516 |
|  |  |  | MR-Egger | 18 | / | / | 0.136* | / |
|  |  |  | Weighted median | 18 | 0.93 | 0.74-1.17 | 0.524 | / |
|  |  |  | Maximum-likelihood method | 18 | 0.97 | 0.82-1.15 | 0.756 | / |
|  |  |  | MR-PRESSO test | 18 | 0.97 | 0.83-1.14 | 0.757 | 0.505 |
| *Lactobacillaceae* | 4.43% | 65.42 |  |  |  |  |  |  |
|  |  |  | Inverse-variance weighted (fixed) | 13 | 1.04 | 0.92-1.18 | 0.511 | 0.236 |
|  |  |  | MR-Egger | 13 | / | / | 0.468* | / |
|  |  |  | Weighted median | 13 | 1.12 | 0.93-1.35 | 0.225 | / |
|  |  |  | Maximum-likelihood method | 13 | 1.05 | 0.90-1.21 | 0.547 | / |
|  |  |  | MR-PRESSO test | 13 | 1.04 | 0.91-1.20 | 0.569 | 0.216 |
| *Methanobacteriaceae* | 7.23% | 119.05 |  |  |  |  |  |  |
|  |  |  | Inverse-variance weighted (fixed) | 12 | 1.05 | 0.96-1.16 | 0.299 | 0.399 |
|  |  |  | MR-Egger | 12 | / | / | 0.583* | / |
|  |  |  | Weighted median | 12 | 1.06 | 0.93-1.21 | 0.358 | / |
|  |  |  | Maximum-likelihood method | 12 | 1.05 | 0.96-1.16 | 0.29 | / |
|  |  |  | MR-PRESSO test | 12 | 1.05 | 0.95-1.16 | 0.332 | 0.397 |
| *Oxalobacteraceae* | 6.93% | 90.93 |  |  |  |  |  |  |
|  |  |  | Inverse-variance weighted (fixed) | 15 | 1.04 | 0.94-1.15 | 0.422 | 0.741 |
|  |  |  | MR-Egger | 15 | / | / | 0.837* | / |
|  |  |  | Weighted median | 15 | 1.06 | 0.92-1.21 | 0.404 | / |
|  |  |  | Maximum-likelihood method | 15 | 1.04 | 0.94-1.15 | 0.406 | / |
|  |  |  | MR-PRESSO test | 15 | 1.04 | 0.96-1.13 | 0.365 | 0.721 |
| *Pasteurellaceae* | 5.52% | 56.29 |  |  |  |  |  |  |
|  |  |  | Inverse-variance weighted (fixed) | 19 | 1.12 | 1.00-1.25 | 0.047 | 0.363 |
|  |  |  | MR-Egger | 19 | / | / | 0.666* | / |
|  |  |  | Weighted median | 19 | 1.17 | 0.99-1.37 | 0.065 | / |
|  |  |  | Maximum-likelihood method | 19 | 1.12 | 1.00-1.26 | 0.05 | / |
|  |  |  | MR-PRESSO test | 19 | 1.12 | 1.00-1.25 | 0.073 | 0.371 |
| *Peptococcaceae* | 2.80% | 52.72 |  |  |  |  |  |  |
|  |  |  | Inverse-variance weighted (fixed) | 10 | 1.08 | 0.92-1.26 | 0.339 | 0.738 |
|  |  |  | MR-Egger | 10 | / | / | 0.223* | / |
|  |  |  | Weighted median | 10 | 1.04 | 0.84-1.29 | 0.712 | / |
|  |  |  | Maximum-likelihood method | 10 | 1.09 | 0.92-1.28 | 0.326 | / |
|  |  |  | MR-PRESSO test | 10 | 1.08 | 0.95-1.23 | 0.272 | 0.643 |
| *Peptostreptococcaceae* | 3.05% | 36.03 |  |  |  |  |  |  |
|  |  |  | Inverse-variance weighted (fixed) | 16 | 1.00 | 0.85-1.17 | 0.961 | 0.03 |
|  |  |  | MR-Egger | 16 | / | / | 0.334* | / |
|  |  |  | Weighted median | 16 | 0.93 | 0.74-1.18 | 0.556 | / |
|  |  |  | Maximum-likelihood method | 16 | 1.00 | 0.80-1.24 | 0.97 | / |
|  |  |  | MR-PRESSO test | 15 | 1.00 | 0.81-1.23 | 0.971 | 0.029 |
| *Porphyromonadaceae* | 1.46% | 22.60 |  |  |  |  |  |  |
|  |  |  | Inverse-variance weighted (fixed) | 12 | 1.05 | 0.86-1.30 | 0.62 | 0.222 |
|  |  |  | MR-Egger | 12 | / | / | 0.560* | / |
|  |  |  | Weighted median | 12 | 1.20 | 0.90-1.60 | 0.213 | / |
|  |  |  | Maximum-likelihood method | 12 | 1.06 | 0.83-1.35 | 0.652 | / |
|  |  |  | MR-PRESSO test | 12 | 1.05 | 0.83-1.34 | 0.671 | 0.239 |
| *Prevotellaceae* | 3.07% | 32.27 |  |  |  |  |  |  |
|  |  |  | Inverse-variance weighted (fixed) | 18 | 0.99 | 0.85-1.16 | 0.948 | 0.157 |
|  |  |  | MR-Egger | 18 | / | / | 0.016* | / |
|  |  |  | Weighted median | 18 | 0.89 | 0.71-1.11 | 0.3 | / |
|  |  |  | Maximum-likelihood method | 18 | 1.00 | 0.83-1.19 | 0.958 | / |
|  |  |  | MR-PRESSO test | 18 | 0.99 | 0.83-1.19 | 0.955 | 0.148 |
| *Rhodospirillaceae* | 3.91% | 46.76 |  |  |  |  |  |  |
|  |  |  | Inverse-variance weighted (fixed) | 17 | 0.98 | 0.87-1.11 | 0.756 | 0.492 |
|  |  |  | MR-Egger | 17 | / | / | 0.329* | / |
|  |  |  | Weighted median | 17 | 0.90 | 0.76-1.06 | 0.212 | / |
|  |  |  | Maximum-likelihood method | 17 | 0.98 | 0.86-1.11 | 0.752 | / |
|  |  |  | MR-PRESSO test | 17 | 0.98 | 0.87-1.11 | 0.755 | 0.51 |
| *Rikenellaceae* | 3.07% | 25.20 |  |  |  |  |  |  |
|  |  |  | Inverse-variance weighted (fixed) | 23 | 1.13 | 0.98-1.32 | 0.102 | 0.533 |
|  |  |  | MR-Egger | 23 | / | / | 0.731* | / |
|  |  |  | Weighted median | 23 | 1.10 | 0.89-1.37 | 0.376 | / |
|  |  |  | Maximum-likelihood method | 23 | 1.14 | 0.98-1.33 | 0.1 | / |
|  |  |  | MR-PRESSO test | 23 | 1.13 | 0.98-1.31 | 0.107 | 0.564 |
| *Ruminococcaceae* | 1.90% | 29.58 |  |  |  |  |  |  |
|  |  |  | Inverse-variance weighted (fixed) | 12 | 0.91 | 0.75-1.11 | 0.354 | 0.063 |
|  |  |  | MR-Egger | 12 | / | / | 0.733* | / |
|  |  |  | Weighted median | 12 | 1.15 | 0.86-1.53 | 0.346 | / |
|  |  |  | Maximum-likelihood method | 12 | 0.91 | 0.70-1.19 | 0.49 | / |
|  |  |  | MR-PRESSO test | 12 | 0.91 | 0.71-1.18 | 0.495 | 0.056 |
| *Streptococcaceae* | 2.70% | 26.71 |  |  |  |  |  |  |
|  |  |  | Inverse-variance weighted (fixed) | 19 | 0.94 | 0.80-1.11 | 0.485 | 0.73 |
|  |  |  | MR-Egger | 19 | / | / | 0.342* | / |
|  |  |  | Weighted median | 19 | 0.98 | 0.78-1.22 | 0.849 | / |
|  |  |  | Maximum-likelihood method | 19 | 0.95 | 0.80-1.11 | 0.502 | / |
|  |  |  | MR-PRESSO test | 19 | 0.94 | 0.82-1.09 | 0.438 | 0.736 |
| *Veillonellaceae* | 3.79% | 34.40 |  |  |  |  |  |  |
|  |  |  | Inverse-variance weighted (fixed) | 21 | 0.89 | 0.78-1.02 | 0.082 | 0.898 |
|  |  |  | MR-Egger | 21 | / | / | 0.420* | / |
|  |  |  | Weighted median | 21 | 0.86 | 0.72-1.04 | 0.129 | / |
|  |  |  | Maximum-likelihood method | 21 | 0.89 | 0.78-1.02 | 0.09 | / |
|  |  |  | MR-PRESSO test | 21 | 0.89 | 0.80-0.99 | 0.039 | 0.9 |
| *Verrucomicrobiaceae* | 2.63% | 38.04 |  |  |  |  |  |  |
|  |  |  | Inverse-variance weighted (fixed) | 13 | 1.02 | 0.87-1.21 | 0.779 | 0.33 |
|  |  |  | MR-Egger | 13 | / | / | 0.103* | / |
|  |  |  | Weighted median | 13 | 1.05 | 0.85-1.31 | 0.647 | / |
|  |  |  | Maximum-likelihood method | 13 | 1.02 | 0.86-1.22 | 0.786 | / |
|  |  |  | MR-PRESSO test | 13 | 1.02 | 0.86-1.22 | 0.796 | 0.318 |
| *Victivallaceae* | 9.17% | 123.35 |  |  |  |  |  |  |
|  |  |  | Inverse-variance weighted (fixed) | 15 | 0.96 | 0.88-1.05 | 0.33 | 0.322 |
|  |  |  | MR-Egger | 15 | / | / | 0.697* | / |
|  |  |  | Weighted median | 15 | 0.91 | 0.80-1.04 | 0.174 | / |
|  |  |  | Maximum-likelihood method | 15 | 0.96 | 0.87-1.05 | 0.364 | / |
|  |  |  | MR-PRESSO test | 15 | 0.96 | 0.87-1.05 | 0.375 | 0.312 |
| **Genus** |  |  |  |  |  |  |  |  |
| *Actinomyces* | 2.66% | 62.61 |  |  |  |  |  |  |
|  |  |  | Inverse-variance weighted (fixed) | 8 | 0.99 | 0.84-1.17 | 0.95 | 0.838 |
|  |  |  | MR-Egger | 8 | / | / | 0.610* | / |
|  |  |  | Weighted median | 8 | 0.99 | 0.81-1.22 | 0.956 | / |
|  |  |  | Maximum-likelihood method | 8 | 0.99 | 0.84-1.17 | 0.949 | / |
|  |  |  | MR-PRESSO test | 8 | 0.99 | 0.89-1.12 | 0.931 | 0.856 |
| *Adlercreutzia* | 3.11% | 49.06 |  |  |  |  |  |  |
|  |  |  | Inverse-variance weighted (fixed) | 12 | 1.01 | 0.87-1.16 | 0.936 | 0.831 |
|  |  |  | MR-Egger | 12 | / | / | 0.829* | / |
|  |  |  | Weighted median | 12 | 0.97 | 0.80-1.18 | 0.785 | / |
|  |  |  | Maximum-likelihood method | 12 | 1.01 | 0.87-1.17 | 0.934 | / |
|  |  |  | MR-PRESSO test | 12 | 1.01 | 0.90-1.13 | 0.919 | 0.837 |
| *Akkermansia* | 2.63% | 38.06 |  |  |  |  |  |  |
|  |  |  | Inverse-variance weighted (fixed) | 13 | 1.02 | 0.87-1.21 | 0.778 | 0.331 |
|  |  |  | MR-Egger | 13 | / | / | 0.102* | / |
|  |  |  | Weighted median | 13 | 1.05 | 0.85-1.31 | 0.645 | / |
|  |  |  | Maximum-likelihood method | 13 | 1.03 | 0.86-1.23 | 0.778 | / |
|  |  |  | MR-PRESSO test | 13 | 1.02 | 0.86-1.22 | 0.795 | 0.342 |
| *Alistipes* | 1.80% | 22.37 |  |  |  |  |  |  |
|  |  |  | Inverse-variance weighted (fixed) | 15 | 1.13 | 0.93-1.37 | 0.211 | 0.316 |
|  |  |  | MR-Egger | 15 | / | / | 0.244* | / |
|  |  |  | Weighted median | 15 | 1.22 | 0.94-1.58 | 0.131 | / |
|  |  |  | Maximum-likelihood method | 15 | 1.13 | 0.92-1.40 | 0.24 | / |
|  |  |  | MR-PRESSO test | 15 | 1.13 | 0.92-1.39 | 0.261 | 0.326 |
| *Allisonella* | 6.06% | 131.44 |  |  |  |  |  |  |
|  |  |  | Inverse-variance weighted (fixed) | 9 | 0.95 | 0.86-1.06 | 0.379 | 0.26 |
|  |  |  | MR-Egger | 9 | / | / | 0.533* | / |
|  |  |  | Weighted median | 9 | 0.92 | 0.81-1.06 | 0.26 | / |
|  |  |  | Maximum-likelihood method | 9 | 0.95 | 0.85-1.07 | 0.428 | / |
|  |  |  | MR-PRESSO test | 9 | 0.95 | 0.85-1.07 | 0.456 | 0.281 |
| *Alloprevotella* | 4.85% | 133.35 |  |  |  |  |  |  |
|  |  |  | Inverse-variance weighted (fixed) | 7 | 0.98 | 0.87-1.09 | 0.668 | 0.655 |
|  |  |  | MR-Egger | 7 | / | / | 0.614* | / |
|  |  |  | Weighted median | 7 | 1.00 | 0.87-1.16 | 0.967 | / |
|  |  |  | Maximum-likelihood method | 7 | 0.97 | 0.87-1.09 | 0.664 | / |
|  |  |  | MR-PRESSO test | 7 | 0.98 | 0.89-1.07 | 0.625 | 0.686 |
| *Anaerofilum* | 5.45% | 88.11 |  |  |  |  |  |  |
|  |  |  | Inverse-variance weighted (fixed) | 12 | 0.93 | 0.83-1.04 | 0.217 | 0.888 |
|  |  |  | MR-Egger | 12 | / | / | 0.715* | / |
|  |  |  | Weighted median | 12 | 0.91 | 0.79-1.05 | 0.209 | / |
|  |  |  | Maximum-likelihood method | 12 | 0.93 | 0.83-1.04 | 0.224 | / |
|  |  |  | MR-PRESSO test | 12 | 0.93 | 0.86-1.01 | 0.116 | 0.908 |
| *Anaerostipes* | 2.19% | 27.40 |  |  |  |  |  |  |
|  |  |  | Inverse-variance weighted (fixed) | 15 | 0.97 | 0.81-1.17 | 0.745 | 0.526 |
|  |  |  | MR-Egger | 15 | / | / | 0.647* | / |
|  |  |  | Weighted median | 15 | 1.04 | 0.82-1.32 | 0.743 | / |
|  |  |  | Maximum-likelihood method | 15 | 0.97 | 0.80-1.17 | 0.741 | / |
|  |  |  | MR-PRESSO test | 15 | 0.97 | 0.81-1.16 | 0.741 | 0.535 |
| *Anaerotruncus* | 2.14% | 25.10 |  |  |  |  |  |  |
|  |  |  | Inverse-variance weighted (fixed) | 15 | 0.87 | 0.73-1.05 | 0.141 | 0.098 |
|  |  |  | MR-Egger | 15 | / | / | 0.296* | / |
|  |  |  | Weighted median | 15 | 0.81 | 0.62-1.06 | 0.122 | / |
|  |  |  | Maximum-likelihood method | 15 | 0.87 | 0.69-1.10 | 0.25 | / |
|  |  |  | MR-PRESSO test | 15 | 0.87 | 0.70-1.09 | 0.251 | 0.098 |
| *Bacteroides* | 1.47% | 27.38 |  |  |  |  |  |  |
|  |  |  | Inverse-variance weighted (fixed) | 12 | 1.02 | 0.82-1.27 | 0.843 | 0.382 |
|  |  |  | MR-Egger | 12 | / | / | 0.932* | / |
|  |  |  | Weighted median | 12 | 0.88 | 0.64-1.20 | 0.407 | / |
|  |  |  | Maximum-likelihood method | 12 | 1.02 | 0.81-1.29 | 0.842 | / |
|  |  |  | MR-PRESSO test | 12 | 1.02 | 0.81-1.28 | 0.851 | 0.377 |
| *Barnesiella* | 2.63% | 31.01 |  |  |  |  |  |  |
|  |  |  | Inverse-variance weighted (fixed) | 17 | 0.90 | 0.77-1.06 | 0.218 | 0.749 |
|  |  |  | MR-Egger | 17 | / | / | 0.316* | / |
|  |  |  | Weighted median | 17 | 0.98 | 0.79-1.22 | 0.852 | / |
|  |  |  | Maximum-likelihood method | 17 | 0.90 | 0.76-1.07 | 0.228 | / |
|  |  |  | MR-PRESSO test | 17 | 0.90 | 0.78-1.04 | 0.173 | 0.724 |
| *Bifidobacterium* | 4.32% | 37.58 |  |  |  |  |  |  |
|  |  |  | Inverse-variance weighted (fixed) | 22 | 1.03 | 0.90-1.18 | 0.683 | 0.85 |
|  |  |  | MR-Egger | 22 | / | / | 0.958* | / |
|  |  |  | Weighted median | 22 | 0.98 | 0.82-1.18 | 0.839 | / |
|  |  |  | Maximum-likelihood method | 22 | 1.03 | 0.90-1.18 | 0.681 | / |
|  |  |  | MR-PRESSO test | 22 | 1.03 | 0.92-1.15 | 0.628 | 0.832 |
| *Bilophila* | 2.82% | 31.30 |  |  |  |  |  |  |
|  |  |  | Inverse-variance weighted (fixed) | 17 | 0.94 | 0.81-1.10 | 0.454 | 0.499 |
|  |  |  | MR-Egger | 17 | / | / | 0.564* | / |
|  |  |  | Weighted median | 17 | 1.02 | 0.83-1.26 | 0.835 | / |
|  |  |  | Maximum-likelihood method | 17 | 0.95 | 0.81-1.10 | 0.474 | / |
|  |  |  | MR-PRESSO test | 17 | 0.94 | 0.81-1.09 | 0.456 | 0.487 |
| *Blautia* | 2.01% | 28.89 |  |  |  |  |  |  |
|  |  |  | Inverse-variance weighted (fixed) | 13 | 1.02 | 0.84-1.23 | 0.841 | 0.326 |
|  |  |  | MR-Egger | 13 | / | / | 0.860* | / |
|  |  |  | Weighted median | 13 | 1.12 | 0.86-1.47 | 0.398 | / |
|  |  |  | Maximum-likelihood method | 13 | 1.02 | 0.83-1.26 | 0.847 | / |
|  |  |  | MR-PRESSO test | 13 | 1.02 | 0.83-1.25 | 0.854 | 0.326 |
| *Butyricicoccus* | 1.95% | 40.56 |  |  |  |  |  |  |
|  |  |  | Inverse-variance weighted (fixed) | 9 | 0.90 | 0.73-1.11 | 0.336 | 0.323 |
|  |  |  | MR-Egger | 9 | / | / | 0.934* | / |
|  |  |  | Weighted median | 9 | 0.94 | 0.71-1.25 | 0.661 | / |
|  |  |  | Maximum-likelihood method | 9 | 0.90 | 0.72-1.13 | 0.375 | / |
|  |  |  | MR-PRESSO test | 9 | 0.90 | 0.72-1.13 | 0.396 | 0.372 |
| *Butyricimonas* | 3.92% | 41.48 |  |  |  |  |  |  |
|  |  |  | Inverse-variance weighted (fixed) | 18 | 1.10 | 0.96-1.26 | 0.163 | 0.208 |
|  |  |  | MR-Egger | 18 | / | / | 0.248* | / |
|  |  |  | Weighted median | 18 | 1.01 | 0.83-1.23 | 0.922 | / |
|  |  |  | Maximum-likelihood method | 18 | 1.11 | 0.95-1.29 | 0.203 | / |
|  |  |  | MR-PRESSO test | 18 | 1.10 | 0.95-1.28 | 0.231 | 0.217 |
| *Butyrivibrio* | 10.11% | 128.73 |  |  |  |  |  |  |
|  |  |  | Inverse-variance weighted (fixed) | 16 | 0.95 | 0.87-1.03 | 0.241 | 0.525 |
|  |  |  | MR-Egger | 16 | / | / | 0.921* | / |
|  |  |  | Weighted median | 16 | 0.98 | 0.88-1.10 | 0.767 | / |
|  |  |  | Maximum-likelihood method | 16 | 0.95 | 0.87-1.04 | 0.248 | / |
|  |  |  | MR-PRESSO test | 16 | 0.95 | 0.88-1.03 | 0.243 | 0.547 |
| *CandidatusSoleaferrea* | 5.83% | 70.84 |  |  |  |  |  |  |
|  |  |  | Inverse-variance weighted (fixed) | 16 | 1.02 | 0.91-1.13 | 0.791 | 0.208 |
|  |  |  | MR-Egger | 16 | / | / | 0.118* | / |
|  |  |  | Weighted median | 16 | 1.07 | 0.91-1.25 | 0.433 | / |
|  |  |  | Maximum-likelihood method | 16 | 1.02 | 0.89-1.16 | 0.806 | / |
|  |  |  | MR-PRESSO test | 16 | 1.02 | 0.90-1.15 | 0.818 | 0.197 |
| *Catenibacterium* | 3.28% | 124.43 |  |  |  |  |  |  |
|  |  |  | Inverse-variance weighted (fixed) | 5 | 1.10 | 0.96-1.26 | 0.173 | 0.586 |
|  |  |  | MR-Egger | 5 | / | / | 0.495* | / |
|  |  |  | Weighted median | 5 | 1.10 | 0.91-1.32 | 0.316 | / |
|  |  |  | Maximum-likelihood method | 5 | 1.10 | 0.96-1.27 | 0.172 | / |
|  |  |  | MR-PRESSO test | 5 | 1.10 | 0.98-1.24 | 0.181 | 0.617 |
| *ChristensenellaceaeR.7* | 1.50% | 25.33 |  |  |  |  |  |  |
|  |  |  | Inverse-variance weighted (fixed) | 11 | 1.07 | 0.86-1.34 | 0.53 | 0.873 |
|  |  |  | MR-Egger | 11 | / | / | 0.582* | / |
|  |  |  | Weighted median | 11 | 1.03 | 0.78-1.36 | 0.846 | / |
|  |  |  | Maximum-likelihood method | 11 | 1.07 | 0.86-1.34 | 0.527 | / |
|  |  |  | MR-PRESSO test | 11 | 1.07 | 0.91-1.26 | 0.407 | 0.874 |
| *Clostridiuminnocuum* | 5.90% | 104.48 |  |  |  |  |  |  |
|  |  |  | Inverse-variance weighted (fixed) | 12 | 0.94 | 0.84-1.04 | 0.232 | 0.694 |
|  |  |  | MR-Egger | 12 | / | / | 0.603* | / |
|  |  |  | Weighted median | 12 | 0.95 | 0.82-1.09 | 0.45 | / |
|  |  |  | Maximum-likelihood method | 12 | 0.93 | 0.84-1.04 | 0.23 | / |
|  |  |  | MR-PRESSO test | 12 | 0.94 | 0.85-1.03 | 0.194 | 0.698 |
| *Clostridiumsensustricto1* | 1.86% | 38.54 |  |  |  |  |  |  |
|  |  |  | Inverse-variance weighted (fixed) | 9 | 0.92 | 0.76-1.12 | 0.406 | 0.053 |
|  |  |  | MR-Egger | 9 | / | / | 0.979* | / |
|  |  |  | Weighted median | 9 | 0.81 | 0.62-1.07 | 0.134 | / |
|  |  |  | Maximum-likelihood method | 9 | 0.92 | 0.70-1.22 | 0.564 | / |
|  |  |  | MR-PRESSO test | 8 | 0.92 | 0.70-1.20 | 0.565 | 0.048 |
| *Collinsella* | 1.93% | 27.67 |  |  |  |  |  |  |
|  |  |  | Inverse-variance weighted (fixed) | 13 | 0.98 | 0.82-1.18 | 0.868 | 0.775 |
|  |  |  | MR-Egger | 13 | / | / | 0.784* | / |
|  |  |  | Weighted median | 13 | 0.97 | 0.76-1.23 | 0.802 | / |
|  |  |  | Maximum-likelihood method | 13 | 0.98 | 0.82-1.19 | 0.869 | / |
|  |  |  | MR-PRESSO test | 13 | 0.98 | 0.85-1.14 | 0.844 | 0.77 |
| *Coprobacter* | 5.29% | 73.18 |  |  |  |  |  |  |
|  |  |  | Inverse-variance weighted (fixed) | 14 | 1.05 | 0.93-1.19 | 0.425 | 0.442 |
|  |  |  | MR-Egger | 14 | / | / | 0.077* | / |
|  |  |  | Weighted median | 14 | 1.08 | 0.90-1.28 | 0.408 | / |
|  |  |  | Maximum-likelihood method | 14 | 1.05 | 0.93-1.19 | 0.405 | / |
|  |  |  | MR-PRESSO test | 14 | 1.05 | 0.93-1.19 | 0.441 | 0.41 |
| *Coprococcus1* | 2.38% | 31.91 |  |  |  |  |  |  |
|  |  |  | Inverse-variance weighted (fixed) | 14 | 0.98 | 0.82-1.17 | 0.827 | 0.591 |
|  |  |  | MR-Egger | 14 | / | / | 0.924* | / |
|  |  |  | Weighted median | 14 | 0.90 | 0.71-1.14 | 0.383 | / |
|  |  |  | Maximum-likelihood method | 14 | 0.98 | 0.82-1.18 | 0.824 | / |
|  |  |  | MR-PRESSO test | 14 | 0.98 | 0.83-1.16 | 0.818 | 0.604 |
| *Coprococcus2* | 2.36% | 36.88 |  |  |  |  |  |  |
|  |  |  | Inverse-variance weighted (fixed) | 12 | 0.95 | 0.80-1.12 | 0.529 | 0.651 |
|  |  |  | MR-Egger | 12 | / | / | 0.529* | / |
|  |  |  | Weighted median | 12 | 0.92 | 0.74-1.16 | 0.482 | / |
|  |  |  | Maximum-likelihood method | 12 | 0.95 | 0.80-1.13 | 0.539 | / |
|  |  |  | MR-PRESSO test | 12 | 0.95 | 0.81-1.10 | 0.494 | 0.656 |
| *Coprococcus3* | 1.62% | 27.50 |  |  |  |  |  |  |
|  |  |  | Inverse-variance weighted (fixed) | 12 | 0.90 | 0.73-1.12 | 0.353 | 0.338 |
|  |  |  | MR-Egger | 12 | / | / | 0.580* | / |
|  |  |  | Weighted median | 12 | 0.85 | 0.65-1.12 | 0.253 | / |
|  |  |  | Maximum-likelihood method | 12 | 0.90 | 0.72-1.13 | 0.372 | / |
|  |  |  | MR-PRESSO test | 12 | 0.90 | 0.72-1.13 | 0.399 | 0.36 |
| *DefluviitaleaceaeUCG011* | 3.18% | 54.72 |  |  |  |  |  |  |
|  |  |  | Inverse-variance weighted (fixed) | 11 | 0.98 | 0.84-1.14 | 0.783 | 0.23 |
|  |  |  | MR-Egger | 11 | / | / | 0.108* | / |
|  |  |  | Weighted median | 11 | 0.95 | 0.76-1.17 | 0.603 | / |
|  |  |  | Maximum-likelihood method | 11 | 0.98 | 0.82-1.17 | 0.809 | / |
|  |  |  | MR-PRESSO test | 11 | 0.98 | 0.82-1.16 | 0.813 | 0.251 |
| *Desulfovibrio* | 3.18% | 50.13 |  |  |  |  |  |  |
|  |  |  | Inverse-variance weighted (fixed) | 12 | 1.10 | 0.95-1.27 | 0.203 | 0.367 |
|  |  |  | MR-Egger | 12 | / | / | 0.294* | / |
|  |  |  | Weighted median | 12 | 1.12 | 0.90-1.39 | 0.303 | / |
|  |  |  | Maximum-likelihood method | 12 | 1.11 | 0.95-1.29 | 0.208 | / |
|  |  |  | MR-PRESSO test | 12 | 1.10 | 0.94-1.28 | 0.248 | 0.352 |
| *Dialister* | 2.16% | 33.65 |  |  |  |  |  |  |
|  |  |  | Inverse-variance weighted (fixed) | 12 | 1.04 | 0.88-1.24 | 0.639 | 0.577 |
|  |  |  | MR-Egger | 12 | / | / | 0.445* | / |
|  |  |  | Weighted median | 12 | 0.90 | 0.71-1.14 | 0.396 | / |
|  |  |  | Maximum-likelihood method | 12 | 1.04 | 0.87-1.25 | 0.63 | / |
|  |  |  | MR-PRESSO test | 12 | 1.04 | 0.89-1.23 | 0.623 | 0.557 |
| *Dorea* | 1.82% | 26.17 |  |  |  |  |  |  |
|  |  |  | Inverse-variance weighted (fixed) | 13 | 0.87 | 0.71-1.06 | 0.16 | 0.865 |
|  |  |  | MR-Egger | 13 | / | / | 0.431* | / |
|  |  |  | Weighted median | 13 | 0.84 | 0.64-1.10 | 0.2 | / |
|  |  |  | Maximum-likelihood method | 13 | 0.87 | 0.71-1.06 | 0.168 | / |
|  |  |  | MR-PRESSO test | 13 | 0.87 | 0.75-1.01 | 0.088 | 0.868 |
| *Eggerthella* | 4.13% | 79.01 |  |  |  |  |  |  |
|  |  |  | Inverse-variance weighted (fixed) | 11 | 0.91 | 0.80-1.03 | 0.137 | 0.175 |
|  |  |  | MR-Egger | 11 | / | / | 0.970* | / |
|  |  |  | Weighted median | 11 | 0.90 | 0.75-1.07 | 0.236 | / |
|  |  |  | Maximum-likelihood method | 11 | 0.91 | 0.78-1.06 | 0.222 | / |
|  |  |  | MR-PRESSO test | 11 | 0.91 | 0.78-1.06 | 0.237 | 0.193 |
| *Eisenbergiella* | 4.52% | 72.37 |  |  |  |  |  |  |
|  |  |  | Inverse-variance weighted (fixed) | 12 | 1.11 | 0.99-1.25 | 0.085 | 0.399 |
|  |  |  | MR-Egger | 12 | / | / | 0.750* | / |
|  |  |  | Weighted median | 12 | 1.08 | 0.92-1.26 | 0.358 | / |
|  |  |  | Maximum-likelihood method | 12 | 1.11 | 0.99-1.26 | 0.082 | / |
|  |  |  | MR-PRESSO test | 12 | 1.11 | 0.98-1.25 | 0.121 | 0.41 |
| *Enterorhabdus* | 3.22% | 67.98 |  |  |  |  |  |  |
|  |  |  | Inverse-variance weighted (fixed) | 10 | 0.95 | 0.83-1.09 | 0.466 | 0.982 |
|  |  |  | MR-Egger | 10 | / | / | 0.135* | / |
|  |  |  | Weighted median | 10 | 0.93 | 0.78-1.10 | 0.397 | / |
|  |  |  | Maximum-likelihood method | 10 | 0.95 | 0.82-1.09 | 0.462 | / |
|  |  |  | MR-PRESSO test | 10 | 0.95 | 0.88-1.02 | 0.197 | 0.969 |
| *Erysipelatoclostridium* | 4.07% | 45.68 |  |  |  |  |  |  |
|  |  |  | Inverse-variance weighted (fixed) | 17 | 0.92 | 0.81-1.05 | 0.231 | 0.707 |
|  |  |  | MR-Egger | 17 | / | / | 0.649* | / |
|  |  |  | Weighted median | 17 | 0.94 | 0.78-1.13 | 0.487 | / |
|  |  |  | Maximum-likelihood method | 17 | 0.92 | 0.81-1.06 | 0.245 | / |
|  |  |  | MR-PRESSO test | 17 | 0.92 | 0.82-1.04 | 0.194 | 0.719 |
| *ErysipelotrichaceaeUCG003* | 3.28% | 34.53 |  |  |  |  |  |  |
|  |  |  | Inverse-variance weighted (fixed) | 18 | 1.02 | 0.88-1.19 | 0.781 | 0.885 |
|  |  |  | MR-Egger | 18 | / | / | 0.904* | / |
|  |  |  | Weighted median | 18 | 1.02 | 0.83-1.25 | 0.859 | / |
|  |  |  | Maximum-likelihood method | 18 | 1.02 | 0.88-1.19 | 0.779 | / |
|  |  |  | MR-PRESSO test | 18 | 1.02 | 0.91-1.15 | 0.727 | 0.889 |
| *Escherichia.Shigella* | 2.97% | 37.39 |  |  |  |  |  |  |
|  |  |  | Inverse-variance weighted (fixed) | 15 | 0.98 | 0.83-1.15 | 0.765 | 0.553 |
|  |  |  | MR-Egger | 15 | / | / | 0.171* | / |
|  |  |  | Weighted median | 15 | 0.96 | 0.76-1.20 | 0.691 | / |
|  |  |  | Maximum-likelihood method | 15 | 0.97 | 0.83-1.15 | 0.763 | / |
|  |  |  | MR-PRESSO test | 15 | 0.98 | 0.84-1.14 | 0.758 | 0.554 |
| *Eubacteriumbrachy* | 5.35% | 103.67 |  |  |  |  |  |  |
|  |  |  | Inverse-variance weighted (fixed) | 11 | 0.91 | 0.82-1.02 | 0.105 | 0.068 |
|  |  |  | MR-Egger | 11 | / | / | 0.940* | / |
|  |  |  | Weighted median | 11 | 0.95 | 0.81-1.11 | 0.517 | / |
|  |  |  | Maximum-likelihood method | 11 | 0.91 | 0.78-1.06 | 0.218 | / |
|  |  |  | MR-PRESSO test | 11 | 0.91 | 0.79-1.06 | 0.246 | 0.082 |
| *Eubacteriumcoprostanoligenes* | 1.95% | 24.32 |  |  |  |  |  |  |
|  |  |  | Inverse-variance weighted (fixed) | 15 | 1.05 | 0.87-1.27 | 0.578 | 0.835 |
|  |  |  | MR-Egger | 15 | / | / | 0.994* | / |
|  |  |  | Weighted median | 15 | 1.06 | 0.82-1.38 | 0.636 | / |
|  |  |  | Maximum-likelihood method | 15 | 1.06 | 0.87-1.28 | 0.572 | / |
|  |  |  | MR-PRESSO test | 15 | 1.05 | 0.91-1.22 | 0.498 | 0.833 |
| *Eubacteriumeligens* | 1.85% | 31.49 |  |  |  |  |  |  |
|  |  |  | Inverse-variance weighted (fixed) | 11 | 1.05 | 0.85-1.30 | 0.63 | 0.014 |
|  |  |  | MR-Egger | 11 | / | / | 0.246* | / |
|  |  |  | Weighted median | 11 | 1.08 | 0.79-1.47 | 0.634 | / |
|  |  |  | Maximum-likelihood method | 11 | 1.06 | 0.76-1.48 | 0.734 | / |
|  |  |  | MR-PRESSO test | 10 | 1.05 | 0.77-1.45 | 0.753 | 0.009 |
| *Eubacteriumfissicatena* | 5.01% | 107.36 |  |  |  |  |  |  |
|  |  |  | Inverse-variance weighted (fixed) | 9 | 0.99 | 0.88-1.11 | 0.895 | 0.409 |
|  |  |  | MR-Egger | 9 | / | / | 0.106* | / |
|  |  |  | Weighted median | 9 | 1.01 | 0.87-1.18 | 0.849 | / |
|  |  |  | Maximum-likelihood method | 9 | 0.99 | 0.88-1.12 | 0.9 | / |
|  |  |  | MR-PRESSO test | 9 | 0.99 | 0.88-1.12 | 0.9 | 0.395 |
| *Eubacteriumhallii* | 2.82% | 33.25 |  |  |  |  |  |  |
|  |  |  | Inverse-variance weighted (fixed) | 16 | 0.99 | 0.83-1.19 | 0.952 | 0.134 |
|  |  |  | MR-Egger | 16 | / | / | 0.806* | / |
|  |  |  | Weighted median | 16 | 0.96 | 0.74-1.25 | 0.763 | / |
|  |  |  | Maximum-likelihood method | 16 | 0.99 | 0.80-1.24 | 0.96 | / |
|  |  |  | MR-PRESSO test | 16 | 0.99 | 0.81-1.23 | 0.961 | 0.147 |
| *Eubacteriumnodatum* | 7.15% | 128.36 |  |  |  |  |  |  |
|  |  |  | Inverse-variance weighted (fixed) | 11 | 0.97 | 0.88-1.07 | 0.565 | 0.546 |
|  |  |  | MR-Egger | 11 | / | / | 0.856* | / |
|  |  |  | Weighted median | 11 | 0.98 | 0.86-1.12 | 0.77 | / |
|  |  |  | Maximum-likelihood method | 11 | 0.97 | 0.88-1.07 | 0.557 | / |
|  |  |  | MR-PRESSO test | 11 | 0.97 | 0.89-1.07 | 0.555 | 0.567 |
| *Eubacteriumoxidoreducens* | 2.20% | 82.61 |  |  |  |  |  |  |
|  |  |  | Inverse-variance weighted (fixed) | 6 | 0.98 | 0.80-1.20 | 0.847 | 0.192 |
|  |  |  | MR-Egger | 6 | / | / | 0.846* | / |
|  |  |  | Weighted median | 6 | 1.08 | 0.84-1.39 | 0.546 | / |
|  |  |  | Maximum-likelihood method | 6 | 0.98 | 0.76-1.26 | 0.872 | / |
|  |  |  | MR-PRESSO test | 6 | 0.98 | 0.77-1.25 | 0.88 | 0.225 |
| *Eubacteriumrectale* | 1.78% | 25.52 |  |  |  |  |  |  |
|  |  |  | Inverse-variance weighted (fixed) | 13 | 0.88 | 0.73-1.06 | 0.185 | 0.076 |
|  |  |  | MR-Egger | 13 | / | / | 0.664* | / |
|  |  |  | Weighted median | 13 | 0.95 | 0.72-1.27 | 0.749 | / |
|  |  |  | Maximum-likelihood method | 13 | 0.88 | 0.69-1.13 | 0.316 | / |
|  |  |  | MR-PRESSO test | 13 | 0.88 | 0.69-1.12 | 0.319 | 0.074 |
| *Eubacteriumruminantium* | 5.88% | 60.26 |  |  |  |  |  |  |
|  |  |  | Inverse-variance weighted (fixed) | 19 | 0.94 | 0.84-1.05 | 0.273 | 0.631 |
|  |  |  | MR-Egger | 19 | / | / | 0.275* | / |
|  |  |  | Weighted median | 19 | 0.86 | 0.74-1.00 | 0.046 | / |
|  |  |  | Maximum-likelihood method | 19 | 0.94 | 0.84-1.05 | 0.286 | / |
|  |  |  | MR-PRESSO test | 19 | 0.94 | 0.85-1.04 | 0.252 | 0.637 |
| *Eubacteriumventriosum* | 2.60% | 28.78 |  |  |  |  |  |  |
|  |  |  | Inverse-variance weighted (fixed) | 17 | 1.06 | 0.89-1.27 | 0.506 | 0.345 |
|  |  |  | MR-Egger | 17 | / | / | 0.533* | / |
|  |  |  | Weighted median | 17 | 1.13 | 0.88-1.45 | 0.346 | / |
|  |  |  | Maximum-likelihood method | 17 | 1.07 | 0.88-1.29 | 0.508 | / |
|  |  |  | MR-PRESSO test | 17 | 1.06 | 0.88-1.28 | 0.535 | 0.341 |
| *Faecalibacterium* | 2.60% | 37.59 |  |  |  |  |  |  |
|  |  |  | Inverse-variance weighted (fixed) | 13 | 1.15 | 0.97-1.37 | 0.111 | 0.546 |
|  |  |  | MR-Egger | 13 | / | / | 0.943* | / |
|  |  |  | Weighted median | 13 | 1.07 | 0.84-1.35 | 0.601 | / |
|  |  |  | Maximum-likelihood method | 13 | 1.16 | 0.97-1.38 | 0.102 | / |
|  |  |  | MR-PRESSO test | 13 | 1.15 | 0.98-1.36 | 0.118 | 0.601 |
| *FamilyXIIIAD3011* | 2.24% | 30.01 |  |  |  |  |  |  |
|  |  |  | Inverse-variance weighted (fixed) | 15 | 1.05 | 0.88-1.26 | 0.577 | 0.369 |
|  |  |  | MR-Egger | 15 | / | / | 0.386* | / |
|  |  |  | Weighted median | 15 | 0.97 | 0.76-1.24 | 0.804 | / |
|  |  |  | Maximum-likelihood method | 15 | 1.06 | 0.87-1.27 | 0.575 | / |
|  |  |  | MR-PRESSO test | 15 | 1.05 | 0.87-1.26 | 0.6 | 0.369 |
| *FamilyXIIIUCG001* | 1.94% | 36.25 |  |  |  |  |  |  |
|  |  |  | Inverse-variance weighted (fixed) | 10 | 1.17 | 0.97-1.42 | 0.105 | 0.187 |
|  |  |  | MR-Egger | 10 | / | / | 0.996* | / |
|  |  |  | Weighted median | 10 | 1.24 | 0.93-1.65 | 0.142 | / |
|  |  |  | Maximum-likelihood method | 10 | 1.18 | 0.93-1.49 | 0.162 | / |
|  |  |  | MR-PRESSO test | 10 | 1.17 | 0.93-1.47 | 0.202 | 0.223 |
| *Flavonifractor* | 2.19% | 40.97 |  |  |  |  |  |  |
|  |  |  | Inverse-variance weighted (fixed) | 10 | 1.04 | 0.87-1.24 | 0.704 | 0.56 |
|  |  |  | MR-Egger | 10 | / | / | 0.658* | / |
|  |  |  | Weighted median | 10 | 1.08 | 0.84-1.38 | 0.547 | / |
|  |  |  | Maximum-likelihood method | 10 | 1.04 | 0.86-1.24 | 0.702 | / |
|  |  |  | MR-PRESSO test | 10 | 1.04 | 0.88-1.22 | 0.691 | 0.545 |
| *Fusicatenibacter* | 2.60% | 24.45 |  |  |  |  |  |  |
|  |  |  | Inverse-variance weighted (fixed) | 20 | 0.94 | 0.79-1.11 | 0.443 | 0.113 |
|  |  |  | MR-Egger | 20 | / | / | 0.050* | / |
|  |  |  | Weighted median | 20 | 0.93 | 0.74-1.18 | 0.567 | / |
|  |  |  | Maximum-likelihood method | 20 | 0.94 | 0.77-1.15 | 0.54 | / |
|  |  |  | MR-PRESSO test | 20 | 0.94 | 0.77-1.14 | 0.525 | 0.103 |
| *Gordonibacter* | 9.26% | 124.67 |  |  |  |  |  |  |
|  |  |  | Inverse-variance weighted (fixed) | 15 | 0.98 | 0.90-1.07 | 0.692 | 0.553 |
|  |  |  | MR-Egger | 15 | / | / | 0.483* | / |
|  |  |  | Weighted median | 15 | 1.02 | 0.91-1.15 | 0.726 | / |
|  |  |  | Maximum-likelihood method | 15 | 0.98 | 0.90-1.07 | 0.704 | / |
|  |  |  | MR-PRESSO test | 15 | 0.98 | 0.91-1.07 | 0.684 | 0.525 |
| *Haemophilus* | 4.10% | 55.89 |  |  |  |  |  |  |
|  |  |  | Inverse-variance weighted (fixed) | 14 | 1.00 | 0.88-1.13 | 0.949 | 0.052 |
|  |  |  | MR-Egger | 14 | / | / | 0.656* | / |
|  |  |  | Weighted median | 14 | 1.10 | 0.91-1.33 | 0.315 | / |
|  |  |  | Maximum-likelihood method | 14 | 1.00 | 0.84-1.18 | 0.96 | / |
|  |  |  | MR-PRESSO test | 14 | 1.00 | 0.85-1.17 | 0.962 | 0.061 |
| *Holdemanella* | 4.79% | 65.87 |  |  |  |  |  |  |
|  |  |  | Inverse-variance weighted (fixed) | 14 | 1.10 | 0.98-1.25 | 0.12 | 0.979 |
|  |  |  | MR-Egger | 14 | / | / | 0.940* | / |
|  |  |  | Weighted median | 14 | 1.12 | 0.96-1.31 | 0.135 | / |
|  |  |  | Maximum-likelihood method | 14 | 1.10 | 0.98-1.25 | 0.114 | / |
|  |  |  | MR-PRESSO test | 14 | 1.10 | 1.02-1.19 | 0.024 | 0.984 |
| *Holdemania* | 4.84% | 51.77 |  |  |  |  |  |  |
|  |  |  | Inverse-variance weighted (fixed) | 18 | 0.95 | 0.84-1.07 | 0.423 | 0.44 |
|  |  |  | MR-Egger | 18 | / | / | 0.225* | / |
|  |  |  | Weighted median | 18 | 0.95 | 0.79-1.13 | 0.531 | / |
|  |  |  | Maximum-likelihood method | 18 | 0.95 | 0.84-1.08 | 0.428 | / |
|  |  |  | MR-PRESSO test | 18 | 0.95 | 0.84-1.07 | 0.437 | 0.429 |
| *Howardella* | 6.28% | 111.64 |  |  |  |  |  |  |
|  |  |  | Inverse-variance weighted (fixed) | 11 | 0.99 | 0.89-1.09 | 0.808 | 0.261 |
|  |  |  | MR-Egger | 11 | / | / | 0.340* | / |
|  |  |  | Weighted median | 11 | 1.01 | 0.87-1.17 | 0.922 | / |
|  |  |  | Maximum-likelihood method | 11 | 0.99 | 0.88-1.11 | 0.825 | / |
|  |  |  | MR-PRESSO test | 11 | 0.99 | 0.88-1.11 | 0.831 | 0.268 |
| *Hungatella* | 2.50% | 93.98 |  |  |  |  |  |  |
|  |  |  | Inverse-variance weighted (fixed) | 5 | 1.05 | 0.89-1.24 | 0.536 | 0.821 |
|  |  |  | MR-Egger | 5 | / | / | 0.903* | / |
|  |  |  | Weighted median | 5 | 1.04 | 0.85-1.27 | 0.7 | / |
|  |  |  | Maximum-likelihood method | 5 | 1.05 | 0.89-1.25 | 0.535 | / |
|  |  |  | MR-PRESSO test | 5 | 1.05 | 0.95-1.17 | 0.374 | 0.853 |
| *Intestinibacter* | 2.71% | 33.98 |  |  |  |  |  |  |
|  |  |  | Inverse-variance weighted (fixed) | 15 | 1.04 | 0.90-1.21 | 0.572 | 0.026 |
|  |  |  | MR-Egger | 15 | / | / | 0.905* | / |
|  |  |  | Weighted median | 15 | 1.01 | 0.81-1.27 | 0.926 | / |
|  |  |  | Maximum-likelihood method | 15 | 1.05 | 0.85-1.30 | 0.653 | / |
|  |  |  | MR-PRESSO test | 14 | 1.04 | 0.85-1.28 | 0.684 | 0.026 |
| *Intestinimonas* | 4.41% | 42.28 |  |  |  |  |  |  |
|  |  |  | Inverse-variance weighted (fixed) | 20 | 1.06 | 0.93-1.21 | 0.37 | 0.772 |
|  |  |  | MR-Egger | 20 | / | / | 0.149* | / |
|  |  |  | Weighted median | 20 | 1.11 | 0.93-1.34 | 0.252 | / |
|  |  |  | Maximum-likelihood method | 20 | 1.07 | 0.93-1.22 | 0.349 | / |
|  |  |  | MR-PRESSO test | 20 | 1.06 | 0.95-1.19 | 0.313 | 0.766 |
| *Lachnoclostridium* | 1.86% | 23.18 |  |  |  |  |  |  |
|  |  |  | Inverse-variance weighted (fixed) | 15 | 0.88 | 0.73-1.07 | 0.199 | 0.622 |
|  |  |  | MR-Egger | 15 | / | / | 0.679* | / |
|  |  |  | Weighted median | 15 | 0.87 | 0.67-1.15 | 0.331 | / |
|  |  |  | Maximum-likelihood method | 15 | 0.88 | 0.73-1.07 | 0.211 | / |
|  |  |  | MR-PRESSO test | 15 | 0.88 | 0.74-1.05 | 0.183 | 0.625 |
| *Lachnospira* | 0.95% | 25.08 |  |  |  |  |  |  |
|  |  |  | Inverse-variance weighted (fixed) | 7 | 1.11 | 0.85-1.44 | 0.462 | 0.742 |
|  |  |  | MR-Egger | 7 | / | / | 0.599* | / |
|  |  |  | Weighted median | 7 | 1.22 | 0.87-1.73 | 0.253 | / |
|  |  |  | Maximum-likelihood method | 7 | 1.11 | 0.85-1.45 | 0.457 | / |
|  |  |  | MR-PRESSO test | 7 | 1.11 | 0.90-1.35 | 0.374 | 0.745 |
| *LachnospiraceaeFCS020* | 3.11% | 34.54 |  |  |  |  |  |  |
|  |  |  | Inverse-variance weighted (fixed) | 17 | 0.94 | 0.81-1.09 | 0.445 | 0.972 |
|  |  |  | MR-Egger | 17 | / | / | 0.972* | / |
|  |  |  | Weighted median | 17 | 0.94 | 0.77-1.14 | 0.531 | / |
|  |  |  | Maximum-likelihood method | 17 | 0.94 | 0.81-1.10 | 0.454 | / |
|  |  |  | MR-PRESSO test | 17 | 0.94 | 0.86-1.04 | 0.267 | 0.981 |
| *LachnospiraceaeNC2004* | 3.49% | 66.37 |  |  |  |  |  |  |
|  |  |  | Inverse-variance weighted (fixed) | 10 | 1.02 | 0.89-1.17 | 0.783 | 0.088 |
|  |  |  | MR-Egger | 10 | / | / | 0.281* | / |
|  |  |  | Weighted median | 10 | 1.08 | 0.88-1.32 | 0.488 | / |
|  |  |  | Maximum-likelihood method | 10 | 1.02 | 0.85-1.23 | 0.819 | / |
|  |  |  | MR-PRESSO test | 10 | 1.02 | 0.86-1.22 | 0.836 | 0.086 |
| *LachnospiraceaeND3007* | 0.57% | 26.17 |  |  |  |  |  |  |
|  |  |  | Inverse-variance weighted (fixed) | 4 | 0.86 | 0.57-1.31 | 0.483 | 0.031 |
|  |  |  | MR-Egger | 4 | / | / | 0.794* | / |
|  |  |  | Weighted median | 4 | 0.80 | 0.46-1.39 | 0.429 | / |
|  |  |  | Maximum-likelihood method | 4 | 0.84 | 0.40-1.80 | 0.661 | / |
|  |  |  | MR-PRESSO test | 3 | 0.86 | 0.42-1.76 | 0.71 | 0.021 |
| *LachnospiraceaeNK4A136* | 2.75% | 32.32 |  |  |  |  |  |  |
|  |  |  | Inverse-variance weighted (fixed) | 16 | 0.87 | 0.74-1.02 | 0.082 | 0.331 |
|  |  |  | MR-Egger | 16 | / | / | 0.507* | / |
|  |  |  | Weighted median | 16 | 0.86 | 0.67-1.10 | 0.236 | / |
|  |  |  | Maximum-likelihood method | 16 | 0.87 | 0.73-1.03 | 0.102 | / |
|  |  |  | MR-PRESSO test | 16 | 0.87 | 0.73-1.03 | 0.121 | 0.32 |
| *LachnospiraceaeUCG001* | 3.38% | 42.75 |  |  |  |  |  |  |
|  |  |  | Inverse-variance weighted (fixed) | 16 | 1.00 | 0.87-1.15 | 0.999 | 0.37 |
|  |  |  | MR-Egger | 16 | / | / | 0.404* | / |
|  |  |  | Weighted median | 16 | 1.05 | 0.86-1.28 | 0.632 | / |
|  |  |  | Maximum-likelihood method | 16 | 1.00 | 0.86-1.16 | 0.999 | / |
|  |  |  | MR-PRESSO test | 16 | 1.00 | 0.86-1.16 | 0.999 | 0.359 |
| *LachnospiraceaeUCG004* | 2.28% | 28.45 |  |  |  |  |  |  |
|  |  |  | Inverse-variance weighted (fixed) | 15 | 0.95 | 0.80-1.13 | 0.554 | 0.526 |
|  |  |  | MR-Egger | 15 | / | / | 0.031* | / |
|  |  |  | Weighted median | 15 | 0.86 | 0.68-1.08 | 0.19 | / |
|  |  |  | Maximum-likelihood method | 15 | 0.95 | 0.80-1.13 | 0.551 | / |
|  |  |  | MR-PRESSO test | 15 | 0.95 | 0.81-1.12 | 0.549 | 0.501 |
| *LachnospiraceaeUCG008* | 4.84% | 66.62 |  |  |  |  |  |  |
|  |  |  | Inverse-variance weighted (fixed) | 14 | 1.16 | 1.03-1.31 | 0.014 | 0.163 |
|  |  |  | MR-Egger | 14 | / | / | 0.661* | / |
|  |  |  | Weighted median | 14 | 1.11 | 0.93-1.33 | 0.235 | / |
|  |  |  | Maximum-likelihood method | 14 | 1.17 | 1.01-1.35 | 0.034 | / |
|  |  |  | MR-PRESSO test | 14 | 1.16 | 1.01-1.34 | 0.055 | 0.188 |
| *LachnospiraceaeUCG010* | 2.52% | 36.40 |  |  |  |  |  |  |
|  |  |  | Inverse-variance weighted (fixed) | 13 | 1.08 | 0.91-1.27 | 0.385 | 0.682 |
|  |  |  | MR-Egger | 13 | / | / | 0.939* | / |
|  |  |  | Weighted median | 13 | 1.11 | 0.88-1.40 | 0.371 | / |
|  |  |  | Maximum-likelihood method | 13 | 1.08 | 0.91-1.28 | 0.375 | / |
|  |  |  | MR-PRESSO test | 13 | 1.08 | 0.93-1.25 | 0.341 | 0.7 |
| *Lactobacillus* | 4.16% | 66.24 |  |  |  |  |  |  |
|  |  |  | Inverse-variance weighted (fixed) | 12 | 1.04 | 0.91-1.19 | 0.546 | 0.192 |
|  |  |  | MR-Egger | 12 | / | / | 0.128* | / |
|  |  |  | Weighted median | 12 | 1.07 | 0.88-1.29 | 0.505 | / |
|  |  |  | Maximum-likelihood method | 12 | 1.04 | 0.89-1.22 | 0.59 | / |
|  |  |  | MR-PRESSO test | 12 | 1.04 | 0.89-1.21 | 0.613 | 0.194 |
| *Lactococcus* | 5.95% | 105.41 |  |  |  |  |  |  |
|  |  |  | Inverse-variance weighted (fixed) | 11 | 1.02 | 0.92-1.13 | 0.753 | 0.433 |
|  |  |  | MR-Egger | 11 | / | / | 0.367* | / |
|  |  |  | Weighted median | 11 | 1.03 | 0.90-1.18 | 0.692 | / |
|  |  |  | Maximum-likelihood method | 11 | 1.02 | 0.92-1.13 | 0.75 | / |
|  |  |  | MR-PRESSO test | 11 | 1.02 | 0.92-1.13 | 0.761 | 0.444 |
| *Marvinbryantia* | 2.43% | 35.13 |  |  |  |  |  |  |
|  |  |  | Inverse-variance weighted (fixed) | 13 | 0.92 | 0.78-1.09 | 0.347 | 0.024 |
|  |  |  | MR-Egger | 13 | / | / | 0.315* | / |
|  |  |  | Weighted median | 13 | 0.80 | 0.63-1.03 | 0.084 | / |
|  |  |  | Maximum-likelihood method | 13 | 0.92 | 0.72-1.18 | 0.512 | / |
|  |  |  | MR-PRESSO test | 12 | 0.92 | 0.73-1.17 | 0.514 | 0.034 |
| *Methanobrevibacter* | 4.61% | 110.62 |  |  |  |  |  |  |
|  |  |  | Inverse-variance weighted (fixed) | 8 | 1.08 | 0.96-1.21 | 0.186 | 0.149 |
|  |  |  | MR-Egger | 8 | / | / | 0.126* | / |
|  |  |  | Weighted median | 8 | 1.04 | 0.89-1.22 | 0.603 | / |
|  |  |  | Maximum-likelihood method | 8 | 1.09 | 0.94-1.26 | 0.268 | / |
|  |  |  | MR-PRESSO test | 8 | 1.08 | 0.94-1.25 | 0.321 | 0.161 |
| *Odoribacter* | 1.45% | 30.01 |  |  |  |  |  |  |
|  |  |  | Inverse-variance weighted (fixed) | 9 | 1.10 | 0.88-1.37 | 0.407 | 0.233 |
|  |  |  | MR-Egger | 9 | / | / | 0.278* | / |
|  |  |  | Weighted median | 9 | 0.93 | 0.68-1.26 | 0.626 | / |
|  |  |  | Maximum-likelihood method | 9 | 1.10 | 0.85-1.43 | 0.456 | / |
|  |  |  | MR-PRESSO test | 9 | 1.10 | 0.85-1.41 | 0.489 | 0.255 |
| *Olsenella* | 6.41% | 114.06 |  |  |  |  |  |  |
|  |  |  | Inverse-variance weighted (fixed) | 11 | 1.01 | 0.91-1.11 | 0.918 | 0.984 |
|  |  |  | MR-Egger | 11 | / | / | 0.928* | / |
|  |  |  | Weighted median | 11 | 0.99 | 0.87-1.12 | 0.842 | / |
|  |  |  | Maximum-likelihood method | 11 | 1.01 | 0.91-1.12 | 0.917 | / |
|  |  |  | MR-PRESSO test | 11 | 1.01 | 0.95-1.06 | 0.852 | 0.983 |
| *Oscillibacter* | 4.29% | 51.30 |  |  |  |  |  |  |
|  |  |  | Inverse-variance weighted (fixed) | 17 | 1.11 | 0.98-1.26 | 0.088 | 0.849 |
|  |  |  | MR-Egger | 17 | / | / | 0.324* | / |
|  |  |  | Weighted median | 17 | 1.14 | 0.96-1.35 | 0.129 | / |
|  |  |  | Maximum-likelihood method | 17 | 1.12 | 0.99-1.27 | 0.082 | / |
|  |  |  | MR-PRESSO test | 17 | 1.11 | 1.01-1.23 | 0.05 | 0.856 |
| *Oscillospira* | 2.17% | 40.72 |  |  |  |  |  |  |
|  |  |  | Inverse-variance weighted (fixed) | 10 | 1.12 | 0.94-1.35 | 0.202 | 0.496 |
|  |  |  | MR-Egger | 10 | / | / | 0.952* | / |
|  |  |  | Weighted median | 10 | 1.13 | 0.88-1.44 | 0.329 | / |
|  |  |  | Maximum-likelihood method | 10 | 1.13 | 0.94-1.36 | 0.196 | / |
|  |  |  | MR-PRESSO test | 10 | 1.12 | 0.95-1.34 | 0.219 | 0.519 |
| *Oxalobacter* | 6.51% | 106.27 |  |  |  |  |  |  |
|  |  |  | Inverse-variance weighted (fixed) | 12 | 1.01 | 0.91-1.12 | 0.865 | 0.212 |
|  |  |  | MR-Egger | 12 | / | / | 0.806* | / |
|  |  |  | Weighted median | 12 | 0.95 | 0.82-1.09 | 0.474 | / |
|  |  |  | Maximum-likelihood method | 12 | 1.01 | 0.90-1.14 | 0.879 | / |
|  |  |  | MR-PRESSO test | 12 | 1.01 | 0.90-1.13 | 0.884 | 0.208 |
| *Parabacteroides* | 1.47% | 27.38 |  |  |  |  |  |  |
|  |  |  | Inverse-variance weighted (fixed) | 10 | 0.90 | 0.73-1.12 | 0.353 | 0.228 |
|  |  |  | MR-Egger | 10 | / | / | 0.010* | / |
|  |  |  | Weighted median | 10 | 0.77 | 0.56-1.06 | 0.114 | / |
|  |  |  | Maximum-likelihood method | 10 | 0.90 | 0.70-1.16 | 0.401 | / |
|  |  |  | MR-PRESSO test | 10 | 0.90 | 0.71-1.15 | 0.437 | 0.233 |
| *Paraprevotella* | 4.40% | 64.84 |  |  |  |  |  |  |
|  |  |  | Inverse-variance weighted (fixed) | 13 | 0.99 | 0.87-1.12 | 0.837 | 0.818 |
|  |  |  | MR-Egger | 13 | / | / | 0.246* | / |
|  |  |  | Weighted median | 13 | 1.06 | 0.91-1.24 | 0.467 | / |
|  |  |  | Maximum-likelihood method | 13 | 0.99 | 0.87-1.12 | 0.834 | / |
|  |  |  | MR-PRESSO test | 13 | 0.99 | 0.90-1.09 | 0.8 | 0.802 |
| *Parasutterella* | 3.69% | 41.32 |  |  |  |  |  |  |
|  |  |  | Inverse-variance weighted (fixed) | 17 | 1.00 | 0.87-1.14 | 0.947 | 0.947 |
|  |  |  | MR-Egger | 17 | / | / | 0.826* | / |
|  |  |  | Weighted median | 17 | 0.98 | 0.83-1.17 | 0.859 | / |
|  |  |  | Maximum-likelihood method | 17 | 1.00 | 0.87-1.14 | 0.948 | / |
|  |  |  | MR-PRESSO test | 17 | 1.00 | 0.91-1.09 | 0.926 | 0.947 |
| *Peptococcus* | 6.62% | 81.14 |  |  |  |  |  |  |
|  |  |  | Inverse-variance weighted (fixed) | 17 | 0.98 | 0.89-1.09 | 0.72 | 0.163 |
|  |  |  | MR-Egger | 17 | / | / | 0.125* | / |
|  |  |  | Weighted median | 17 | 0.95 | 0.82-1.10 | 0.482 | / |
|  |  |  | Maximum-likelihood method | 17 | 0.98 | 0.87-1.11 | 0.761 | / |
|  |  |  | MR-PRESSO test | 17 | 0.98 | 0.87-1.10 | 0.76 | 0.155 |
| *Phascolarctobacterium* | 2.71% | 39.22 |  |  |  |  |  |  |
|  |  |  | Inverse-variance weighted (fixed) | 13 | 0.97 | 0.83-1.14 | 0.717 | 0.189 |
|  |  |  | MR-Egger | 13 | / | / | 0.673* | / |
|  |  |  | Weighted median | 13 | 0.96 | 0.76-1.20 | 0.693 | / |
|  |  |  | Maximum-likelihood method | 13 | 0.97 | 0.81-1.17 | 0.747 | / |
|  |  |  | MR-PRESSO test | 13 | 0.97 | 0.81-1.16 | 0.76 | 0.172 |
| *Prevotella7* | 7.34% | 121.04 |  |  |  |  |  |  |
|  |  |  | Inverse-variance weighted (fixed) | 12 | 1.11 | 1.01-1.23 | 0.032 | 0.374 |
|  |  |  | MR-Egger | 12 | / | / | 0.673* | / |
|  |  |  | Weighted median | 12 | 1.15 | 1.00-1.31 | 0.051 | / |
|  |  |  | Maximum-likelihood method | 12 | 1.12 | 1.01-1.24 | 0.037 | / |
|  |  |  | MR-PRESSO test | 12 | 1.11 | 1.01-1.23 | 0.064 | 0.411 |
| *Prevotella9* | 5.00% | 48.25 |  |  |  |  |  |  |
|  |  |  | Inverse-variance weighted (fixed) | 20 | 1.06 | 0.94-1.20 | 0.354 | 0.006 |
|  |  |  | MR-Egger | 20 | / | / | 0.002* | / |
|  |  |  | Weighted median | 20 | 1.12 | 0.91-1.38 | 0.287 | / |
|  |  |  | Maximum-likelihood method | 20 | 1.06 | 0.89-1.28 | 0.506 | / |
|  |  |  | MR-PRESSO test | 19 | 1.06 | 0.89-1.26 | 0.52 | 0.005 |
| *RikenellaceaeRC9* | 9.72% | 131.46 |  |  |  |  |  |  |
|  |  |  | Inverse-variance weighted (fixed) | 15 | 1.01 | 0.93-1.10 | 0.793 | 0.47 |
|  |  |  | MR-Egger | 15 | / | / | 0.717* | / |
|  |  |  | Weighted median | 15 | 1.02 | 0.90-1.15 | 0.807 | / |
|  |  |  | Maximum-likelihood method | 15 | 1.01 | 0.93-1.11 | 0.793 | / |
|  |  |  | MR-PRESSO test | 15 | 1.01 | 0.93-1.10 | 0.795 | 0.467 |
| *Romboutsia* | 2.87% | 36.08 |  |  |  |  |  |  |
|  |  |  | Inverse-variance weighted (fixed) | 15 | 1.01 | 0.86-1.19 | 0.892 | 0.97 |
|  |  |  | MR-Egger | 15 | / | / | 0.561* | / |
|  |  |  | Weighted median | 15 | 1.02 | 0.83-1.26 | 0.852 | / |
|  |  |  | Maximum-likelihood method | 15 | 1.01 | 0.86-1.20 | 0.892 | / |
|  |  |  | MR-PRESSO test | 15 | 1.01 | 0.91-1.13 | 0.837 | 0.973 |
| *Roseburia* | 2.57% | 26.88 |  |  |  |  |  |  |
|  |  |  | Inverse-variance weighted (fixed) | 18 | 0.93 | 0.78-1.10 | 0.404 | 0.673 |
|  |  |  | MR-Egger | 18 | / | / | 0.688* | / |
|  |  |  | Weighted median | 18 | 0.94 | 0.74-1.19 | 0.621 | / |
|  |  |  | Maximum-likelihood method | 18 | 0.93 | 0.78-1.11 | 0.4 | / |
|  |  |  | MR-PRESSO test | 18 | 0.93 | 0.79-1.09 | 0.369 | 0.712 |
| *Ruminiclostridium5* | 2.06% | 25.73 |  |  |  |  |  |  |
|  |  |  | Inverse-variance weighted (fixed) | 15 | 1.16 | 0.97-1.39 | 0.105 | 0.974 |
|  |  |  | MR-Egger | 15 | / | / | 0.552* | / |
|  |  |  | Weighted median | 15 | 1.14 | 0.90-1.46 | 0.279 | / |
|  |  |  | Maximum-likelihood method | 15 | 1.17 | 0.97-1.40 | 0.099 | / |
|  |  |  | MR-PRESSO test | 15 | 1.16 | 1.04-1.30 | 0.023 | 0.967 |
| *Ruminiclostridium6* | 3.28% | 36.50 |  |  |  |  |  |  |
|  |  |  | Inverse-variance weighted (fixed) | 17 | 0.82 | 0.70-0.95 | 0.009 | 0.598 |
|  |  |  | MR-Egger | 17 | / | / | 0.441* | / |
|  |  |  | Weighted median | 17 | 0.85 | 0.69-1.05 | 0.128 | / |
|  |  |  | Maximum-likelihood method | 17 | 0.82 | 0.70-0.95 | 0.011 | / |
|  |  |  | MR-PRESSO test | 17 | 0.82 | 0.71-0.94 | 0.013 | 0.63 |
| *Ruminiclostridium9* | 2.06% | 25.75 |  |  |  |  |  |  |
|  |  |  | Inverse-variance weighted (fixed) | 16 | 1.12 | 0.93-1.33 | 0.226 | 0.526 |
|  |  |  | MR-Egger | 16 | / | / | 0.297* | / |
|  |  |  | Weighted median | 16 | 1.07 | 0.83-1.36 | 0.611 | / |
|  |  |  | Maximum-likelihood method | 16 | 1.12 | 0.93-1.34 | 0.221 | / |
|  |  |  | MR-PRESSO test | 16 | 1.12 | 0.94-1.32 | 0.229 | 0.58 |
| *RuminococcaceaeNK4A214* | 2.76% | 28.93 |  |  |  |  |  |  |
|  |  |  | Inverse-variance weighted (fixed) | 18 | 1.08 | 0.92-1.27 | 0.369 | 0.367 |
|  |  |  | MR-Egger | 18 | / | / | 0.683* | / |
|  |  |  | Weighted median | 18 | 1.08 | 0.85-1.36 | 0.541 | / |
|  |  |  | Maximum-likelihood method | 18 | 1.08 | 0.91-1.29 | 0.373 | / |
|  |  |  | MR-PRESSO test | 18 | 1.08 | 0.91-1.28 | 0.399 | 0.367 |
| *RuminococcaceaeUCG002* | 3.86% | 28.25 |  |  |  |  |  |  |
|  |  |  | Inverse-variance weighted (fixed) | 26 | 0.92 | 0.81-1.06 | 0.26 | 0.965 |
|  |  |  | MR-Egger | 26 | / | / | 0.848* | / |
|  |  |  | Weighted median | 26 | 0.96 | 0.80-1.16 | 0.696 | / |
|  |  |  | Maximum-likelihood method | 26 | 0.93 | 0.81-1.06 | 0.27 | / |
|  |  |  | MR-PRESSO test | 26 | 0.92 | 0.84-1.02 | 0.142 | 0.968 |
| *RuminococcaceaeUCG003* | 2.28% | 30.60 |  |  |  |  |  |  |
|  |  |  | Inverse-variance weighted (fixed) | 14 | 0.94 | 0.79-1.12 | 0.494 | 0.958 |
|  |  |  | MR-Egger | 14 | / | / | 0.208* | / |
|  |  |  | Weighted median | 14 | 0.93 | 0.75-1.17 | 0.551 | / |
|  |  |  | Maximum-likelihood method | 14 | 0.94 | 0.79-1.12 | 0.505 | / |
|  |  |  | MR-PRESSO test | 14 | 0.94 | 0.84-1.05 | 0.319 | 0.937 |
| *RuminococcaceaeUCG004* | 2.57% | 43.94 |  |  |  |  |  |  |
|  |  |  | Inverse-variance weighted (fixed) | 12 | 1.15 | 1.00-1.34 | 0.056 | 0.735 |
|  |  |  | MR-Egger | 12 | / | / | 0.866* | / |
|  |  |  | Weighted median | 12 | 1.10 | 0.91-1.34 | 0.319 | / |
|  |  |  | Maximum-likelihood method | 12 | 1.16 | 1.00-1.34 | 0.056 | / |
|  |  |  | MR-PRESSO test | 12 | 1.15 | 1.02-1.30 | 0.044 | 0.766 |
| *RuminococcaceaeUCG005* | 2.73% | 30.20 |  |  |  |  |  |  |
|  |  |  | Inverse-variance weighted (fixed) | 17 | 0.87 | 0.74-1.01 | 0.071 | 0.247 |
|  |  |  | MR-Egger | 17 | / | / | 0.024* | / |
|  |  |  | Weighted median | 17 | 0.85 | 0.68-1.06 | 0.142 | / |
|  |  |  | Maximum-likelihood method | 17 | 0.87 | 0.73-1.04 | 0.116 | / |
|  |  |  | MR-PRESSO test | 17 | 0.87 | 0.73-1.03 | 0.121 | 0.245 |
| *RuminococcaceaeUCG009* | 4.30% | 59.42 |  |  |  |  |  |  |
|  |  |  | Inverse-variance weighted (fixed) | 14 | 0.99 | 0.87-1.13 | 0.862 | 0.139 |
|  |  |  | MR-Egger | 14 | / | / | 0.920* | / |
|  |  |  | Weighted median | 14 | 0.89 | 0.74-1.08 | 0.242 | / |
|  |  |  | Maximum-likelihood method | 14 | 0.99 | 0.84-1.16 | 0.881 | / |
|  |  |  | MR-PRESSO test | 14 | 0.99 | 0.85-1.16 | 0.886 | 0.128 |
| *RuminococcaceaeUCG010* | 1.51% | 35.03 |  |  |  |  |  |  |
|  |  |  | Inverse-variance weighted (fixed) | 8 | 1.01 | 0.81-1.26 | 0.9 | 0.142 |
|  |  |  | MR-Egger | 8 | / | / | 0.905* | / |
|  |  |  | Weighted median | 8 | 1.07 | 0.79-1.45 | 0.653 | / |
|  |  |  | Maximum-likelihood method | 8 | 1.02 | 0.76-1.35 | 0.918 | / |
|  |  |  | MR-PRESSO test | 8 | 1.01 | 0.77-1.34 | 0.923 | 0.143 |
| *RuminococcaceaeUCG011* | 4.90% | 118.09 |  |  |  |  |  |  |
|  |  |  | Inverse-variance weighted (fixed) | 8 | 1.00 | 0.89-1.12 | 0.991 | 0.395 |
|  |  |  | MR-Egger | 8 | / | / | 0.816* | / |
|  |  |  | Weighted median | 8 | 1.02 | 0.88-1.19 | 0.784 | / |
|  |  |  | Maximum-likelihood method | 8 | 1.00 | 0.89-1.13 | 0.992 | / |
|  |  |  | MR-PRESSO test | 8 | 1.00 | 0.89-1.12 | 0.992 | 0.376 |
| *RuminococcaceaeUCG013* | 2.09% | 26.01 |  |  |  |  |  |  |
|  |  |  | Inverse-variance weighted (fixed) | 15 | 0.99 | 0.83-1.17 | 0.873 | 0.022 |
|  |  |  | MR-Egger | 15 | / | / | 0.047* | / |
|  |  |  | Weighted median | 15 | 1.19 | 0.91-1.55 | 0.211 | / |
|  |  |  | Maximum-likelihood method | 15 | 0.99 | 0.77-1.26 | 0.907 | / |
|  |  |  | MR-PRESSO test | 14 | 0.99 | 0.78-1.25 | 0.909 | 0.019 |
| *RuminococcaceaeUCG014* | 2.88% | 31.95 |  |  |  |  |  |  |
|  |  |  | Inverse-variance weighted (fixed) | 18 | 0.95 | 0.81-1.10 | 0.471 | 0.08 |
|  |  |  | MR-Egger | 18 | / | / | 0.836* | / |
|  |  |  | Weighted median | 18 | 0.97 | 0.78-1.22 | 0.818 | / |
|  |  |  | Maximum-likelihood method | 18 | 0.95 | 0.78-1.15 | 0.58 | / |
|  |  |  | MR-PRESSO test | 18 | 0.95 | 0.79-1.14 | 0.565 | 0.081 |
| *Ruminococcus1* | 2.14% | 28.60 |  |  |  |  |  |  |
|  |  |  | Inverse-variance weighted (fixed) | 14 | 1.04 | 0.86-1.27 | 0.665 | 0.33 |
|  |  |  | MR-Egger | 14 | / | / | 0.957* | / |
|  |  |  | Weighted median | 14 | 1.13 | 0.86-1.48 | 0.396 | / |
|  |  |  | Maximum-likelihood method | 14 | 1.05 | 0.85-1.29 | 0.671 | / |
|  |  |  | MR-PRESSO test | 14 | 1.04 | 0.85-1.28 | 0.69 | 0.337 |
| *Ruminococcus2* | 2.76% | 34.63 |  |  |  |  |  |  |
|  |  |  | Inverse-variance weighted (fixed) | 15 | 0.91 | 0.77-1.08 | 0.296 | 0.628 |
|  |  |  | MR-Egger | 15 | / | / | 0.259* | / |
|  |  |  | Weighted median | 15 | 0.89 | 0.70-1.12 | 0.302 | / |
|  |  |  | Maximum-likelihood method | 15 | 0.91 | 0.77-1.09 | 0.322 | / |
|  |  |  | MR-PRESSO test | 15 | 0.91 | 0.78-1.07 | 0.273 | 0.638 |
| *Ruminococcusgauvreauii* | 2.15% | 31.00 |  |  |  |  |  |  |
|  |  |  | Inverse-variance weighted (fixed) | 13 | 0.91 | 0.76-1.09 | 0.313 | 0.041 |
|  |  |  | MR-Egger | 13 | / | / | 0.649* | / |
|  |  |  | Weighted median | 13 | 0.89 | 0.68-1.17 | 0.408 | / |
|  |  |  | Maximum-likelihood method | 13 | 0.91 | 0.71-1.17 | 0.464 | / |
|  |  |  | MR-PRESSO test | 12 | 0.91 | 0.72-1.16 | 0.468 | 0.045 |
| *Ruminococcusgnavus* | 4.08% | 64.94 |  |  |  |  |  |  |
|  |  |  | Inverse-variance weighted (fixed) | 12 | 1.02 | 0.90-1.16 | 0.725 | 0.359 |
|  |  |  | MR-Egger | 12 | / | / | 0.580* | / |
|  |  |  | Weighted median | 12 | 1.01 | 0.85-1.20 | 0.907 | / |
|  |  |  | Maximum-likelihood method | 12 | 1.02 | 0.90-1.17 | 0.722 | / |
|  |  |  | MR-PRESSO test | 12 | 1.02 | 0.90-1.17 | 0.743 | 0.348 |
| *Ruminococcustorques* | 2.08% | 26.00 |  |  |  |  |  |  |
|  |  |  | Inverse-variance weighted (fixed) | 15 | 0.92 | 0.77-1.11 | 0.391 | 0.525 |
|  |  |  | MR-Egger | 15 | / | / | 0.168* | / |
|  |  |  | Weighted median | 15 | 0.96 | 0.75-1.22 | 0.737 | / |
|  |  |  | Maximum-likelihood method | 15 | 0.93 | 0.77-1.11 | 0.41 | / |
|  |  |  | MR-PRESSO test | 15 | 0.92 | 0.78-1.10 | 0.389 | 0.527 |
| *Sellimonas* | 9.32% | 144.84 |  |  |  |  |  |  |
|  |  |  | Inverse-variance weighted (fixed) | 13 | 0.99 | 0.90-1.08 | 0.761 | 0.922 |
|  |  |  | MR-Egger | 13 | / | / | 0.802* | / |
|  |  |  | Weighted median | 13 | 1.00 | 0.89-1.13 | 0.967 | / |
|  |  |  | Maximum-likelihood method | 13 | 0.99 | 0.90-1.08 | 0.758 | / |
|  |  |  | MR-PRESSO test | 13 | 0.99 | 0.93-1.05 | 0.671 | 0.906 |
| *Senegalimassilia* | 2.58% | 60.59 |  |  |  |  |  |  |
|  |  |  | Inverse-variance weighted (fixed) | 8 | 1.01 | 0.86-1.18 | 0.934 | 0.452 |
|  |  |  | MR-Egger | 8 | / | / | 0.820* | / |
|  |  |  | Weighted median | 8 | 1.05 | 0.85-1.29 | 0.653 | / |
|  |  |  | Maximum-likelihood method | 8 | 1.01 | 0.86-1.18 | 0.934 | / |
|  |  |  | MR-PRESSO test | 8 | 1.01 | 0.86-1.18 | 0.936 | 0.462 |
| *Slackia* | 2.81% | 66.15 |  |  |  |  |  |  |
|  |  |  | Inverse-variance weighted (fixed) | 9 | 1.05 | 0.91-1.21 | 0.543 | 0.165 |
|  |  |  | MR-Egger | 9 | / | / | 0.708* | / |
|  |  |  | Weighted median | 9 | 1.10 | 0.89-1.35 | 0.381 | / |
|  |  |  | Maximum-likelihood method | 9 | 1.05 | 0.88-1.26 | 0.597 | / |
|  |  |  | MR-PRESSO test | 9 | 1.05 | 0.88-1.24 | 0.629 | 0.168 |
| *Streptococcus* | 2.68% | 26.56 |  |  |  |  |  |  |
|  |  |  | Inverse-variance weighted (fixed) | 19 | 0.90 | 0.77-1.06 | 0.216 | 0.31 |
|  |  |  | MR-Egger | 19 | / | / | 0.654* | / |
|  |  |  | Weighted median | 19 | 0.98 | 0.77-1.24 | 0.852 | / |
|  |  |  | Maximum-likelihood method | 19 | 0.90 | 0.76-1.08 | 0.258 | / |
|  |  |  | MR-PRESSO test | 19 | 0.90 | 0.76-1.07 | 0.261 | 0.329 |
| *Subdoligranulum* | 1.86% | 24.81 |  |  |  |  |  |  |
|  |  |  | Inverse-variance weighted (fixed) | 14 | 0.83 | 0.69-1.00 | 0.052 | 0.308 |
|  |  |  | MR-Egger | 14 | / | / | 0.534* | / |
|  |  |  | Weighted median | 14 | 0.96 | 0.74-1.25 | 0.785 | / |
|  |  |  | Maximum-likelihood method | 14 | 0.83 | 0.68-1.01 | 0.065 | / |
|  |  |  | MR-PRESSO test | 14 | 0.83 | 0.68-1.02 | 0.094 | 0.307 |
| *Sutterella* | 1.97% | 30.70 |  |  |  |  |  |  |
|  |  |  | Inverse-variance weighted (fixed) | 12 | 0.99 | 0.83-1.18 | 0.909 | 0.805 |
|  |  |  | MR-Egger | 12 | / | / | 0.913* | / |
|  |  |  | Weighted median | 12 | 1.00 | 0.79-1.27 | 1 | / |
|  |  |  | Maximum-likelihood method | 12 | 0.99 | 0.83-1.18 | 0.909 | / |
|  |  |  | MR-PRESSO test | 12 | 0.99 | 0.86-1.14 | 0.889 | 0.812 |
| *Terrisporobacter* | 2.14% | 66.94 |  |  |  |  |  |  |
|  |  |  | Inverse-variance weighted (fixed) | 6 | 0.94 | 0.79-1.12 | 0.469 | 0.044 |
|  |  |  | MR-Egger | 6 | / | / | 0.923* | / |
|  |  |  | Weighted median | 6 | 0.91 | 0.72-1.14 | 0.412 | / |
|  |  |  | Maximum-likelihood method | 6 | 0.93 | 0.71-1.23 | 0.629 | / |
|  |  |  | MR-PRESSO test | 6 | 0.94 | 0.72-1.22 | 0.652 | 0.067 |
| *Turicibacter* | 3.35% | 45.43 |  |  |  |  |  |  |
|  |  |  | Inverse-variance weighted (fixed) | 14 | 1.03 | 0.90-1.19 | 0.639 | 0.792 |
|  |  |  | MR-Egger | 14 | / | / | 0.202* | / |
|  |  |  | Weighted median | 14 | 1.11 | 0.93-1.32 | 0.258 | / |
|  |  |  | Maximum-likelihood method | 14 | 1.03 | 0.90-1.19 | 0.633 | / |
|  |  |  | MR-PRESSO test | 14 | 1.03 | 0.92-1.16 | 0.577 | 0.818 |
| *Tyzzerella3* | 6.44% | 90.12 |  |  |  |  |  |  |
|  |  |  | Inverse-variance weighted (fixed) | 14 | 0.99 | 0.89-1.10 | 0.786 | 0.154 |
|  |  |  | MR-Egger | 14 | / | / | 0.481* | / |
|  |  |  | Weighted median | 14 | 1.05 | 0.91-1.22 | 0.506 | / |
|  |  |  | Maximum-likelihood method | 14 | 0.98 | 0.87-1.12 | 0.818 | / |
|  |  |  | MR-PRESSO test | 14 | 0.99 | 0.87-1.12 | 0.821 | 0.158 |
| *Veillonella* | 2.77% | 47.44 |  |  |  |  |  |  |
|  |  |  | Inverse-variance weighted (fixed) | 11 | 1.13 | 0.95-1.34 | 0.159 | 0.108 |
|  |  |  | MR-Egger | 11 | / | / | 0.008* | / |
|  |  |  | Weighted median | 11 | 1.08 | 0.86-1.36 | 0.489 | / |
|  |  |  | Maximum-likelihood method | 11 | 1.14 | 0.91-1.41 | 0.256 | / |
|  |  |  | MR-PRESSO test | 11 | 1.13 | 0.91-1.40 | 0.288 | 0.109 |
| *Victivallis* | 7.29% | 130.96 |  |  |  |  |  |  |
|  |  |  | Inverse-variance weighted (fixed) | 13 | 1.03 | 0.93-1.13 | 0.594 | 0.668 |
|  |  |  | MR-Egger | 13 | / | / | 0.435* | / |
|  |  |  | Weighted median | 13 | 1.02 | 0.90-1.16 | 0.717 | / |
|  |  |  | Maximum-likelihood method | 13 | 1.03 | 0.93-1.13 | 0.59 | / |
|  |  |  | MR-PRESSO test | 13 | 1.03 | 0.94-1.11 | 0.558 | 0.665 |
| **Order** |  |  |  |  |  |  |  |  |
| *Actinomycetales* | 1.75% | 65.35 |  |  |  |  |  |  |
|  |  |  | Inverse-variance weighted (fixed) | 5 | 1.10 | 0.88-1.37 | 0.404 | 0.898 |
|  |  |  | MR-Egger | 5 | / | / | 0.857* | / |
|  |  |  | Weighted median | 5 | 1.13 | 0.87-1.47 | 0.361 | / |
|  |  |  | Maximum-likelihood method | 5 | 1.10 | 0.88-1.37 | 0.403 | / |
|  |  |  | MR-PRESSO test | 5 | 1.10 | 0.98-1.23 | 0.183 | 0.898 |
| *Bacillales* | 7.14% | 128.04 |  |  |  |  |  |  |
|  |  |  | Inverse-variance weighted (fixed) | 11 | 0.96 | 0.87-1.05 | 0.375 | 0.145 |
|  |  |  | MR-Egger | 11 | / | / | 0.877* | / |
|  |  |  | Weighted median | 11 | 0.95 | 0.82-1.08 | 0.421 | / |
|  |  |  | Maximum-likelihood method | 11 | 0.96 | 0.85-1.08 | 0.468 | / |
|  |  |  | MR-PRESSO test | 11 | 0.96 | 0.85-1.08 | 0.48 | 0.144 |
| *Bacteroidales* | 2.28% | 28.54 |  |  |  |  |  |  |
|  |  |  | Inverse-variance weighted (fixed) | 16 | 1.16 | 0.98-1.37 | 0.093 | 0.665 |
|  |  |  | MR-Egger | 16 | / | / | 0.896* | / |
|  |  |  | Weighted median | 16 | 1.16 | 0.92-1.47 | 0.203 | / |
|  |  |  | Maximum-likelihood method | 16 | 1.16 | 0.98-1.38 | 0.084 | / |
|  |  |  | MR-PRESSO test | 16 | 1.16 | 0.99-1.35 | 0.082 | 0.683 |
| *Bifidobacteriales* | 4.44% | 34.00 |  |  |  |  |  |  |
|  |  |  | Inverse-variance weighted (fixed) | 25 | 0.93 | 0.82-1.06 | 0.298 | 0.953 |
|  |  |  | MR-Egger | 25 | / | / | 0.206* | / |
|  |  |  | Weighted median | 25 | 0.91 | 0.76-1.10 | 0.32 | / |
|  |  |  | Maximum-likelihood method | 25 | 0.93 | 0.82-1.07 | 0.316 | / |
|  |  |  | MR-PRESSO test | 25 | 0.93 | 0.84-1.03 | 0.181 | 0.96 |
| *Burkholderiales* | 1.76% | 25.27 |  |  |  |  |  |  |
|  |  |  | Inverse-variance weighted (fixed) | 13 | 1.18 | 0.97-1.43 | 0.094 | 0.411 |
|  |  |  | MR-Egger | 13 | / | / | 0.393* | / |
|  |  |  | Weighted median | 13 | 1.19 | 0.92-1.55 | 0.191 | / |
|  |  |  | Maximum-likelihood method | 13 | 1.19 | 0.97-1.46 | 0.091 | / |
|  |  |  | MR-PRESSO test | 13 | 1.18 | 0.97-1.44 | 0.126 | 0.426 |
| *Clostridiales* | 1.97% | 21.66 |  |  |  |  |  |  |
|  |  |  | Inverse-variance weighted (fixed) | 17 | 0.92 | 0.76-1.11 | 0.404 | 0.276 |
|  |  |  | MR-Egger | 17 | / | / | 0.730* | / |
|  |  |  | Weighted median | 17 | 0.98 | 0.75-1.29 | 0.896 | / |
|  |  |  | Maximum-likelihood method | 17 | 0.92 | 0.75-1.14 | 0.462 | / |
|  |  |  | MR-PRESSO test | 17 | 0.92 | 0.75-1.13 | 0.453 | 0.266 |
| *Coriobacteriales* | 2.99% | 26.93 |  |  |  |  |  |  |
|  |  |  | Inverse-variance weighted (fixed) | 21 | 1.03 | 0.88-1.21 | 0.689 | 0.746 |
|  |  |  | MR-Egger | 21 | / | / | 0.851* | / |
|  |  |  | Weighted median | 21 | 0.98 | 0.78-1.23 | 0.85 | / |
|  |  |  | Maximum-likelihood method | 21 | 1.03 | 0.88-1.22 | 0.686 | / |
|  |  |  | MR-PRESSO test | 21 | 1.03 | 0.90-1.19 | 0.655 | 0.74 |
| *Desulfovibrionales* | 2.13% | 30.69 |  |  |  |  |  |  |
|  |  |  | Inverse-variance weighted (fixed) | 13 | 1.05 | 0.88-1.25 | 0.593 | 0.594 |
|  |  |  | MR-Egger | 13 | / | / | 0.306* | / |
|  |  |  | Weighted median | 13 | 1.18 | 0.92-1.51 | 0.188 | / |
|  |  |  | Maximum-likelihood method | 13 | 1.05 | 0.88-1.25 | 0.584 | / |
|  |  |  | MR-PRESSO test | 13 | 1.05 | 0.89-1.23 | 0.574 | 0.629 |
| *Enterobacteriales* | 1.84% | 31.17 |  |  |  |  |  |  |
|  |  |  | Inverse-variance weighted (fixed) | 11 | 1.35 | 1.09-1.66 | 0.005 | 0.721 |
|  |  |  | MR-Egger | 11 | / | / | 0.657* | / |
|  |  |  | Weighted median | 11 | 1.35 | 1.03-1.77 | 0.03 | / |
|  |  |  | Maximum-likelihood method | 11 | 1.36 | 1.10-1.69 | 0.005 | / |
|  |  |  | MR-PRESSO test | 11 | 1.35 | 1.13-1.60 | 0.008 | 0.732 |
| *Erysipelotrichales* | 1.65% | 23.71 |  |  |  |  |  |  |
|  |  |  | Inverse-variance weighted (fixed) | 13 | 0.96 | 0.78-1.19 | 0.734 | 0.913 |
|  |  |  | MR-Egger | 13 | / | / | 0.189* | / |
|  |  |  | Weighted median | 13 | 0.94 | 0.72-1.22 | 0.63 | / |
|  |  |  | Maximum-likelihood method | 13 | 0.96 | 0.78-1.19 | 0.737 | / |
|  |  |  | MR-PRESSO test | 13 | 0.96 | 0.83-1.12 | 0.642 | 0.908 |
| *Gastranaerophilales* | 4.90% | 78.66 |  |  |  |  |  |  |
|  |  |  | Inverse-variance weighted (fixed) | 12 | 1.06 | 0.95-1.19 | 0.279 | 0.27 |
|  |  |  | MR-Egger | 12 | / | / | 0.941* | / |
|  |  |  | Weighted median | 12 | 1.14 | 0.97-1.32 | 0.103 | / |
|  |  |  | Maximum-likelihood method | 12 | 1.07 | 0.94-1.21 | 0.316 | / |
|  |  |  | MR-PRESSO test | 12 | 1.06 | 0.94-1.21 | 0.347 | 0.275 |
| *Lactobacillales* | 3.12% | 31.02 |  |  |  |  |  |  |
|  |  |  | Inverse-variance weighted (fixed) | 19 | 0.96 | 0.82-1.13 | 0.627 | 0.752 |
|  |  |  | MR-Egger | 19 | / | / | 0.767* | / |
|  |  |  | Weighted median | 19 | 0.93 | 0.75-1.16 | 0.528 | / |
|  |  |  | Maximum-likelihood method | 19 | 0.96 | 0.81-1.13 | 0.626 | / |
|  |  |  | MR-PRESSO test | 19 | 0.96 | 0.83-1.11 | 0.584 | 0.776 |
| *Methanobacteriales* | 7.23% | 119.05 |  |  |  |  |  |  |
|  |  |  | Inverse-variance weighted (fixed) | 12 | 1.05 | 0.96-1.16 | 0.299 | 0.399 |
|  |  |  | MR-Egger | 12 | / | / | 0.583* | / |
|  |  |  | Weighted median | 12 | 1.06 | 0.93-1.21 | 0.358 | / |
|  |  |  | Maximum-likelihood method | 12 | 1.05 | 0.96-1.16 | 0.29 | / |
|  |  |  | MR-PRESSO test | 12 | 1.05 | 0.95-1.16 | 0.332 | 0.399 |
| *MollicutesRF9* | 3.80% | 42.57 |  |  |  |  |  |  |
|  |  |  | Inverse-variance weighted (fixed) | 17 | 1.06 | 0.93-1.21 | 0.415 | 0.794 |
|  |  |  | MR-Egger | 17 | / | / | 0.608* | / |
|  |  |  | Weighted median | 17 | 1.04 | 0.87-1.25 | 0.659 | / |
|  |  |  | Maximum-likelihood method | 17 | 1.06 | 0.93-1.21 | 0.395 | / |
|  |  |  | MR-PRESSO test | 17 | 1.06 | 0.95-1.18 | 0.346 | 0.804 |
| *NB1n* | 7.59% | 88.52 |  |  |  |  |  |  |
|  |  |  | Inverse-variance weighted (fixed) | 17 | 1.06 | 0.96-1.16 | 0.234 | 0.439 |
|  |  |  | MR-Egger | 17 | / | / | 0.979* | / |
|  |  |  | Weighted median | 17 | 1.03 | 0.91-1.17 | 0.661 | / |
|  |  |  | Maximum-likelihood method | 17 | 1.06 | 0.97-1.17 | 0.216 | / |
|  |  |  | MR-PRESSO test | 17 | 1.06 | 0.96-1.16 | 0.254 | 0.454 |
| *Pasteurellales* | 5.52% | 56.29 |  |  |  |  |  |  |
|  |  |  | Inverse-variance weighted (fixed) | 19 | 1.12 | 1.00-1.25 | 0.047 | 0.363 |
|  |  |  | MR-Egger | 19 | / | / | 0.666* | / |
|  |  |  | Weighted median | 19 | 1.17 | 0.99-1.37 | 0.065 | / |
|  |  |  | Maximum-likelihood method | 19 | 1.12 | 1.00-1.26 | 0.05 | / |
|  |  |  | MR-PRESSO test | 19 | 1.12 | 1.00-1.25 | 0.073 | 0.369 |
| *Rhodospirillales* | 3.39% | 45.92 |  |  |  |  |  |  |
|  |  |  | Inverse-variance weighted (fixed) | 15 | 0.98 | 0.86-1.12 | 0.802 | 0.351 |
|  |  |  | MR-Egger | 15 | / | / | 0.241* | / |
|  |  |  | Weighted median | 15 | 0.87 | 0.73-1.04 | 0.133 | / |
|  |  |  | Maximum-likelihood method | 15 | 0.98 | 0.85-1.13 | 0.807 | / |
|  |  |  | MR-PRESSO test | 15 | 0.98 | 0.86-1.13 | 0.814 | 0.367 |
| *Selenomonadales* | 1.67% | 23.92 |  |  |  |  |  |  |
|  |  |  | Inverse-variance weighted (fixed) | 13 | 0.89 | 0.73-1.09 | 0.278 | 0.397 |
|  |  |  | MR-Egger | 13 | / | / | 0.086* | / |
|  |  |  | Weighted median | 13 | 0.97 | 0.74-1.26 | 0.808 | / |
|  |  |  | Maximum-likelihood method | 13 | 0.90 | 0.73-1.10 | 0.303 | / |
|  |  |  | MR-PRESSO test | 13 | 0.89 | 0.73-1.10 | 0.311 | 0.421 |
| *Verrucomicrobiales* | 2.63% | 38.04 |  |  |  |  |  |  |
|  |  |  | Inverse-variance weighted (fixed) | 13 | 1.02 | 0.87-1.21 | 0.778 | 0.33 |
|  |  |  | MR-Egger | 13 | / | / | 0.102* | / |
|  |  |  | Weighted median | 13 | 1.05 | 0.85-1.31 | 0.646 | / |
|  |  |  | Maximum-likelihood method | 13 | 1.02 | 0.86-1.22 | 0.786 | / |
|  |  |  | MR-PRESSO test | 13 | 1.02 | 0.86-1.22 | 0.795 | 0.356 |
| *Victivallales* | 4.81% | 92.58 |  |  |  |  |  |  |
|  |  |  | Inverse-variance weighted (fixed) | 10 | 1.04 | 0.92-1.18 | 0.488 | 0.735 |
|  |  |  | MR-Egger | 10 | / | / | 0.428* | / |
|  |  |  | Weighted median | 10 | 1.04 | 0.89-1.22 | 0.622 | / |
|  |  |  | Maximum-likelihood method | 10 | 1.05 | 0.92-1.18 | 0.481 | / |
|  |  |  | MR-PRESSO test | 10 | 1.04 | 0.94-1.15 | 0.419 | 0.711 |
| **Phylum** |  |  |  |  |  |  |  |  |
| *Actinobacteria* | 2.80% | 26.43 |  |  |  |  |  |  |
|  |  |  | Inverse-variance weighted (fixed) | 20 | 0.91 | 0.77-1.07 | 0.256 | 0.934 |
|  |  |  | MR-Egger | 20 | / | / | 0.960* | / |
|  |  |  | Weighted median | 20 | 0.93 | 0.74-1.16 | 0.521 | / |
|  |  |  | Maximum-likelihood method | 20 | 0.91 | 0.78-1.08 | 0.275 | / |
|  |  |  | MR-PRESSO test | 20 | 0.91 | 0.81-1.03 | 0.146 | 0.933 |
| *Bacteroidetes* | 2.21% | 29.61 |  | 0 |  |  |  |  |
|  |  |  | Inverse-variance weighted (fixed) | 14 | 1.15 | 0.96-1.37 | 0.131 | 0.933 |
|  |  |  | MR-Egger | 14 | / | / | 0.600* | / |
|  |  |  | Weighted median | 14 | 1.13 | 0.88-1.45 | 0.324 | / |
|  |  |  | Maximum-likelihood method | 14 | 1.15 | 0.96-1.38 | 0.121 | / |
|  |  |  | MR-PRESSO test | 14 | 1.15 | 1.01-1.30 | 0.05 | 0.932 |
| *Cyanobacteria* | 3.53% | 67.16 |  |  |  |  |  |  |
|  |  |  | Inverse-variance weighted (fixed) | 10 | 0.96 | 0.84-1.10 | 0.594 | 0.294 |
|  |  |  | MR-Egger | 10 | / | / | 0.038* | / |
|  |  |  | Weighted median | 10 | 0.93 | 0.76-1.13 | 0.444 | / |
|  |  |  | Maximum-likelihood method | 10 | 0.96 | 0.83-1.12 | 0.617 | / |
|  |  |  | MR-PRESSO test | 10 | 0.96 | 0.83-1.12 | 0.637 | 0.28 |
| *Euryarchaeota* | 7.39% | 112.50 |  |  |  |  |  |  |
|  |  |  | Inverse-variance weighted (fixed) | 13 | 1.00 | 0.91-1.10 | 0.927 | 0.632 |
|  |  |  | MR-Egger | 13 | / | / | 0.758* | / |
|  |  |  | Weighted median | 13 | 0.97 | 0.85-1.10 | 0.624 | / |
|  |  |  | Maximum-likelihood method | 13 | 1.00 | 0.91-1.11 | 0.925 | / |
|  |  |  | MR-PRESSO test | 13 | 1.00 | 0.92-1.09 | 0.92 | 0.623 |
| *Firmicutes* | 2.49% | 24.65 |  |  |  |  |  |  |
|  |  |  | Inverse-variance weighted (fixed) | 20 | 1.00 | 0.85-1.18 | 0.975 | 0.054 |
|  |  |  | MR-Egger | 20 | / | / | 0.194* | / |
|  |  |  | Weighted median | 20 | 0.99 | 0.77-1.27 | 0.953 | / |
|  |  |  | Maximum-likelihood method | 20 | 1.00 | 0.81-1.25 | 0.978 | / |
|  |  |  | MR-PRESSO test | 20 | 1.00 | 0.81-1.24 | 0.98 | 0.057 |
| *Lentisphaerae* | 5.25% | 92.39 |  |  |  |  |  |  |
|  |  |  | Inverse-variance weighted (fixed) | 11 | 1.05 | 0.94-1.18 | 0.382 | 0.792 |
|  |  |  | MR-Egger | 11 | / | / | 0.382* | / |
|  |  |  | Weighted median | 11 | 1.06 | 0.92-1.23 | 0.421 | / |
|  |  |  | Maximum-likelihood method | 11 | 1.05 | 0.94-1.19 | 0.374 | / |
|  |  |  | MR-PRESSO test | 11 | 1.05 | 0.96-1.15 | 0.296 | 0.765 |
| *Proteobacteria* | 1.39% | 28.61 |  |  |  |  |  |  |
|  |  |  | Inverse-variance weighted (fixed) | 14 | 1.03 | 0.84-1.26 | 0.768 | 0.556 |
|  |  |  | MR-Egger | 14 | / | / | 0.631* | / |
|  |  |  | Weighted median | 14 | 1.04 | 0.79-1.37 | 0.769 | / |
|  |  |  | Maximum-likelihood method | 14 | 1.03 | 0.84-1.27 | 0.757 | / |
|  |  |  | MR-PRESSO test | 14 | 1.03 | 0.85-1.25 | 0.76 | 0.553 |
| *Tenericutes* | 2.60% | 40.77 |  |  |  |  |  |  |
|  |  |  | Inverse-variance weighted (fixed) | 12 | 0.95 | 0.81-1.12 | 0.549 | 0.341 |
|  |  |  | MR-Egger | 12 | / | / | 0.036* | / |
|  |  |  | Weighted median | 12 | 0.98 | 0.78-1.23 | 0.837 | / |
|  |  |  | Maximum-likelihood method | 12 | 0.95 | 0.80-1.13 | 0.574 | / |
|  |  |  | MR-PRESSO test | 12 | 0.95 | 0.81-1.13 | 0.582 | 0.372 |
| *Verrucomicrobia* | 2.42% | 37.94 |  |  |  |  |  |  |
|  |  |  | Inverse-variance weighted (fixed) | 12 | 0.99 | 0.84-1.17 | 0.884 | 0.235 |
|  |  |  | MR-Egger | 12 | / | / | 0.037* | / |
|  |  |  | Weighted median | 12 | 1.01 | 0.80-1.27 | 0.954 | / |
|  |  |  | Maximum-likelihood method | 12 | 0.99 | 0.82-1.20 | 0.899 | / |
|  |  |  | MR-PRESSO test | 12 | 0.99 | 0.82-1.19 | 0.899 | 0.263 |

**Abbreviations:** *CI*, confidence interval; *MR*, Mendelian randomization; *MR-PRESSO test*, MR Pleiotropy RESidual Sum and Outlier test; *OR*, odds ratio; *SNP*, single nucleotide polymorphism.

**Note:** **p*-value of the intercept from MR-Egger regression analysis.

# Table S5. The results of relevant confounding factors for included SNPs obtained from the PhenoScanner database.

| **SNP** | **Association trait** | **Beta** | ***p*-value** | **N** | **Trait type** | **PMID** |
| --- | --- | --- | --- | --- | --- | --- |
| **rs12124567** | Diastolic blood pressure | 0.012 | 6.09E-06 | 317756 | Cardiovascular disease | UKBB |
| **rs57404562** | Self-reported uterine fibroids | -0.002 | 1.4E-06 | 337159 | Tumor or cancer | UKBB |
| **rs9608249** | Time spent using computer | -0.014 | 1.8E-06 | 261987 | Life style | UKBB |
| **rs2548459** | Self-reported high cholesterol | 0.006 | 2.4E-15 | 337159 | Metabolic feature | UKBB |
|  | Alkaline phosphatase | -0.010 | 9.96E-13 | 9961 | Metabolic feature | 28887542 |
|  | Cholelithiasis | 0.002 | 7.53E-11 | 337199 | Digestive disease | UKBB |
|  | Mean platelet volume | -0.023 | 8.1E-10 | 173480 | Blood routine | 27863252 |
|  | Sodium in urine | -0.014 | 6.86E-09 | 326831 | Metabolic feature | UKBB |
|  | Alcohol intake frequency | -0.021 | 8E-09 | 336965 | Life style | UKBB |
|  | Treatment with simvastatin | 0.004 | 1.02E-08 | 337159 | Metabolic feature | UKBB |
|  | Bring up phlegm or sputum/mucus on most days | 0.008 | 1.87E-08 | 83529 | Respiratory disease | UKBB |
|  | Crohn’s disease | 0.132 | 3.38E-08 | 20883 | Digestive disease | 26192919 |
|  | Comparative height size at age 10 | -0.009 | 6.31E-08 | 332021 | Physical feature | UKBB |
|  | Self-reported hypertension | 0.006 | 8.18E-08 | 337159 | Cardiovascular disease | UKBB |
|  | Medication for cholesterol, blood pressure or diabetes:  cholesterol lowering medication | 0.008 | 1.17E-07 | 154702 | Cardiovascular disease | UKBB |
|  | Mouth or teeth dental problems: dentures | 0.005 | 1.18E-07 | 336138 | Stomatology disease | UKBB |
|  | Peak expiratory flow | -0.011 | 2.6E-07 | 307638 | Respiratory disease | UKBB |
|  | Comparative body size at age 10 | -0.008 | 5.02E-07 | 331693 | Physical feature | UKBB |
|  | Self-reported cholelithiasis or gall stones | 0.002 | 6.46E-07 | 337159 | Digestive disease | UKBB |
|  | No treatment with medication for cholesterol, blood pressure, diabetes, or take exogenous hormones | -0.008 | 6.94E-07 | 180203 | Cardiovascular disease | UKBB |
|  | Treatment with cholesterol lowering medication | 0.005 | 8.86E-07 | 180203 | Metabolic feature | UKBB |
|  | Alcohol intake versus 10 years previously | -0.009 | 1.21E-06 | 313248 | Life style | UKBB |
|  | Vascular or heart problems diagnosed by doctor: none of the above | -0.005 | 1.24E-06 | 336683 | Cardiovascular disease | UKBB |
|  | Mouth or teeth dental problems: none of the above | -0.006 | 3.36E-06 | 336138 | Stomatology disease | UKBB |
|  | Number of self-reported non-cancer illnesses | 0.009 | 3.99E-06 | 337159 | Tumor or cancer | UKBB |
|  | Vascular or heart problems diagnosed by doctor: high blood pressure | 0.005 | 4.85E-06 | 336683 | Cardiovascular disease | UKBB |
|  | Medication for cholesterol, blood pressure or diabetes: none of the above | -0.008 | 6.99E-06 | 154702 | Cardiovascular disease | UKBB |
|  | Sitting height | -0.008 | 7.38E-06 | 336172 | Physical feature | UKBB |
| **rs67479537** | Cause of death: cerebrovascular disease, unspecified | -0.005 | 9.85E-06 | 7637 | Cardiovascular disease | UKBB |
| **rs11992182** | Lymphocyte count | 0.020 | 5.5E-07 | 173480 | Blood routine | 27863252 |
| **rs10751237** | Sitting height | 0.012 | 1.42E-09 | 336172 | Physical feature | UKBB |
|  | Height | 0.009 | 2.22E-07 | 336474 | Physical feature | UKBB |
| **rs57091572** | Height | 0.026 | 4.56E-28 | 336474 | Physical feature | UKBB |
|  | Sitting height | 0.028 | 2.22E-27 | 336172 | Physical feature | UKBB |
|  | Comparative height size at age 10 | 0.016 | 1.2E-12 | 332021 | Physical feature | UKBB |
|  | Trunk fat-free mass | 0.010 | 1.58E-06 | 331030 | Physical feature | UKBB |
|  | Trunk predicted mass | 0.010 | 2.63E-06 | 330995 | Physical feature | UKBB |
|  | Forced expiratory volume in 1-second, predicted | 0.017 | 9.66E-06 | 110423 | Respiratory disease | UKBB |
| **rs61508842** | Treatment with epilim 100 mg crushable tablet | 0.001 | 8.63E-06 | 337159 | Nerve disease | UKBB |
| **rs4792380** | Self-reported Sjogren syndrome or sicca syndrome | 0.001 | 2.95E-06 | 337159 | Immunological disease | UKBB |
| **rs2976950** | Red cell distribution width | -0.025 | 3.15E-12 | 173480 | Blood routine | 27863252 |
|  | Heel bone mineral density | -0.022 | 6.94E-12 | 194398 | Orthopedic disease | UKBB |
|  | Neutrophil count | 0.022 | 9.4E-10 | 173480 | Blood routine | 27863252 |
|  | Sum basophil neutrophil counts | 0.022 | 1.12E-09 | 173480 | Blood routine | 27863252 |
|  | Myeloid white cell count | 0.022 | 1.56E-09 | 173480 | Blood routine | 27863252 |
|  | Sum neutrophil eosinophil counts | 0.022 | 1.74E-09 | 173480 | Blood routine | 27863252 |
|  | Granulocyte count | 0.022 | 1.81E-09 | 173480 | Blood routine | 27863252 |
|  | Heel bone mineral density right | -0.025 | 2.15E-09 | 106251 | Orthopedic disease | UKBB |
|  | Impedance of whole body | 0.011 | 2.73E-09 | 331284 | Nerve disease | UKBB |
|  | Worrier or anxious feelings | 0.007 | 2.78E-09 | 328717 | Psychological feature | UKBB |
|  | Neuroticism score | 0.052 | 3.29E-09 | 274108 | Psychological feature | UKBB |
|  | Systolic blood pressure | -0.015 | 3.36E-09 | 317754 | Cardiovascular disease | UKBB |
|  | Impedance of arm right | 0.010 | 6.79E-09 | 331279 | Nerve disease | UKBB |
|  | White blood cell count | 0.021 | 1.09E-08 | 173480 | Blood routine | 27863252 |
|  | Impedance of leg right | 0.013 | 1.38E-08 | 331301 | Nerve disease | UKBB |
|  | Impedance of leg left | 0.013 | 2.16E-08 | 331296 | Nerve disease | UKBB |
|  | Platelet distribution width | -0.020 | 6E-08 | 173480 | Blood routine | 27863252 |
|  | Diastolic blood pressure | -0.014 | 6.02E-08 | 317756 | Cardiovascular disease | UKBB |
|  | Impedance of arm left | 0.009 | 1.38E-07 | 331292 | Nerve disease | UKBB |
|  | Heel bone mineral density left | -0.022 | 3.52E-07 | 106254 | Orthopedic disease | UKBB |
|  | Basophil count | 0.018 | 8.09E-07 | 173480 | Blood routine | 27863252 |
|  | Irritability | 0.006 | 1.25E-06 | 322668 | Psychological feature | UKBB |
|  | Vascular or heart problems diagnosed by doctor: high blood pressure | -0.005 | 1.65E-06 | 336683 | Cardiovascular disease | UKBB |
|  | Nervous feelings | 0.005 | 2.88E-06 | 328725 | Psychological feature | UKBB |
|  | Body mass index | -0.011 | 4.12E-06 | 336107 | Physical feature | UKBB |
|  | Neuroticism | 0.011 | 6.72E-06 | 168105 | Psychological feature | 29292387 |
| **rs10840326** | Platelet distribution width | -0.017 | 2.76E-06 | 173480 | Blood routine | 27863252 |
| **rs12050685** | Pulse rate | 0.031 | 3.1E-33 | 317756 | Physical feature | UKBB |
|  | Leg fat percentage right | 0.008 | 1.59E-07 | 331296 | Physical feature | UKBB |
|  | Leg fat percentage left | 0.008 | 4.02E-07 | 331278 | Physical feature | UKBB |
|  | Heart rate | 0.287 | 6.04E-07 | 84398 | Physical feature | 23583979 |
|  | Heart rate variability RMSSD | -0.025 | 1.65E-06 | 26366 | Physical feature | 28613276 |
|  | Heart rate variability SDNN | -0.017 | 3.55E-06 | 27256 | Physical feature | 28613276 |
|  | Body fat percentage | 0.008 | 9.55E-06 | 331117 | Physical feature | UKBB |

**Abbreviations:** *Chr*, chromosome; *SE*, standard error; *SNP*, single nucleotide polymorphism.

#

# **Table S6.** Effect estimates of the associations between periodontitis and genetically predicted gut microbiome traits in the reverse MR analysis.

| **Gut microbiota** | **Method** | **nSNP** | **OR** | **95% CI** | ***p*-value** | **Qrs** |
| --- | --- | --- | --- | --- | --- | --- |
| **Class** |  |  |  |  |  |  |
| *Actinobacteria* |  |  |  |  |  |  |
|  | Inverse-variance weighted (fixed) | 4 | 1.01 | 0.90-1.13 | 0.869 | 0.550 |
|  | MR-Egger | 4 | / | / | 0.479* | / |
|  | Weighted median | 4 | 1.00 | 0.87-1.15 | 0.965 | / |
|  | Maximum-likelihood method | 4 | 1.01 | 0.90-1.13 | 0.868 | / |
|  | MR-PRESSO test | 4 | 1.01 | 0.92-1.11 | 0.857 | 0.536 |
| *Alphaproteobacteria* |  |  |  |  |  |  |
|  | Inverse-variance weighted (fixed) | 4 | 1.05 | 0.91-1.22 | 0.503 | 0.834 |
|  | MR-Egger | 4 | / | / | 0.687* | / |
|  | Weighted median | 4 | 1.06 | 0.89-1.26 | 0.513 | / |
|  | Maximum-likelihood method | 4 | 1.05 | 0.91-1.22 | 0.502 | / |
|  | MR-PRESSO test | 4 | 1.05 | 0.97-1.14 | 0.301 | 0.816 |
| *Alphaproteobacteria* |  |  |  |  |  |  |
|  | Inverse-variance weighted (fixed) | 4 | 1.11 | 0.99-1.23 | 0.075 | 0.088 |
|  | MR-Egger | 4 | / | / | 0.329* | / |
|  | Weighted median | 4 | 1.12 | 0.96-1.29 | 0.140 | / |
|  | Maximum-likelihood method | 4 | 1.11 | 0.94-1.32 | 0.217 | / |
|  | MR-PRESSO test | 4 | 1.11 | 0.94-1.30 | 0.315 | 0.119 |
| *Bacteroidia* |  |  |  |  |  |  |
|  | Inverse-variance weighted (fixed) | 4 | 0.99 | 0.89-1.11 | 0.898 | 0.247 |
|  | MR-Egger | 4 | / | / | 0.781* | / |
|  | Weighted median | 4 | 1.02 | 0.90-1.17 | 0.727 | / |
|  | Maximum-likelihood method | 4 | 0.99 | 0.87-1.13 | 0.912 | / |
|  | MR-PRESSO test | 4 | 0.99 | 0.88-1.13 | 0.920 | 0.311 |
| *Betaproteobacteria* |  |  |  |  |  |  |
|  | Inverse-variance weighted (fixed) | 4 | 0.94 | 0.84-1.05 | 0.267 | 0.960 |
|  | MR-Egger | 4 | / | / | 0.677* | / |
|  | Weighted median | 4 | 0.95 | 0.83-1.08 | 0.395 | / |
|  | Maximum-likelihood method | 4 | 0.94 | 0.84-1.05 | 0.270 | / |
|  | MR-PRESSO test | 4 | 0.94 | 0.91-0.97 | 0.039 | 0.959 |
| *Clostridia* |  |  |  |  |  |  |
|  | Inverse-variance weighted (fixed) | 4 | 1.01 | 0.90-1.12 | 0.918 | 0.131 |
|  | MR-Egger | 4 | / | / | 0.706* | / |
|  | Weighted median | 4 | 1.00 | 0.87-1.15 | 0.982 | / |
|  | Maximum-likelihood method | 4 | 1.01 | 0.87-1.17 | 0.938 | / |
|  | MR-PRESSO test | 4 | 1.01 | 0.87-1.16 | 0.945 | 0.189 |
| *Coriobacteriia* |  |  |  |  |  |  |
|  | Inverse-variance weighted (fixed) | 4 | 0.99 | 0.88-1.10 | 0.796 | 0.373 |
|  | MR-Egger | 4 | / | / | 0.922* | / |
|  | Weighted median | 4 | 0.96 | 0.83-1.10 | 0.524 | / |
|  | Maximum-likelihood method | 4 | 0.99 | 0.88-1.10 | 0.797 | / |
|  | MR-PRESSO test | 4 | 0.99 | 0.88-1.10 | 0.816 | 0.417 |
| *Deltaproteobacteria* |  |  |  |  |  |  |
|  | Inverse-variance weighted (fixed) | 4 | 1.01 | 0.90-1.13 | 0.903 | 0.812 |
|  | MR-Egger | 4 | / | / | 0.927* | / |
|  | Weighted median | 4 | 1.02 | 0.89-1.17 | 0.785 | / |
|  | Maximum-likelihood method | 4 | 1.01 | 0.90-1.13 | 0.902 | / |
|  | MR-PRESSO test | 4 | 1.01 | 0.94-1.08 | 0.842 | 0.847 |
| *Erysipelotrichia* |  |  |  |  |  |  |
|  | Inverse-variance weighted (fixed) | 4 | 0.96 | 0.86-1.06 | 0.408 | 0.428 |
|  | MR-Egger | 4 | / | / | 0.141* | / |
|  | Weighted median | 4 | 0.91 | 0.79-1.05 | 0.190 | / |
|  | Maximum-likelihood method | 4 | 0.95 | 0.86-1.06 | 0.402 | / |
|  | MR-PRESSO test | 4 | 0.96 | 0.86-1.06 | 0.453 | 0.428 |
| *Gammaproteobacteria* |  |  |  |  |  |  |
|  | Inverse-variance weighted (fixed) | 4 | 0.95 | 0.85-1.07 | 0.428 | 0.415 |
|  | MR-Egger | 4 | / | / | 0.678* | / |
|  | Weighted median | 4 | 0.96 | 0.83-1.10 | 0.532 | / |
|  | Maximum-likelihood method | 4 | 0.95 | 0.85-1.07 | 0.424 | / |
|  | MR-PRESSO test | 4 | 0.95 | 0.85-1.07 | 0.476 | 0.487 |
| *Lentisphaeria* |  |  |  |  |  |  |
|  | Inverse-variance weighted (fixed) | 4 | 1.12 | 0.91-1.38 | 0.271 | 0.287 |
|  | MR-Egger | 4 | / | / | 0.647* | / |
|  | Weighted median | 4 | 1.07 | 0.82-1.39 | 0.640 | / |
|  | Maximum-likelihood method | 4 | 1.13 | 0.89-1.43 | 0.319 | / |
|  | MR-PRESSO test | 4 | 1.12 | 0.89-1.42 | 0.399 | 0.331 |
| *Melainabacteria* |  |  |  |  |  |  |
|  | Inverse-variance weighted (fixed) | 4 | 0.93 | 0.77-1.12 | 0.435 | 0.552 |
|  | MR-Egger | 4 | / | / | 0.288* | / |
|  | Weighted median | 4 | 0.88 | 0.70-1.11 | 0.294 | / |
|  | Maximum-likelihood method | 4 | 0.93 | 0.76-1.12 | 0.431 | / |
|  | MR-PRESSO test | 4 | 0.93 | 0.79-1.09 | 0.419 | 0.498 |
| *Methanobacteria* |  |  |  |  |  |  |
|  | Inverse-variance weighted (fixed) | 3 | 0.82 | 0.60-1.10 | 0.185 | 0.127 |
|  | MR-Egger | 3 | 0.50 | / | 0.764* | / |
|  | Weighted median | 3 | 0.86 | 0.57-1.30 | 0.471 | / |
|  | Maximum-likelihood method | 3 | 0.81 | 0.59-1.10 | 0.178 | / |
| *Mollicutes* |  |  |  |  |  |  |
|  | Inverse-variance weighted (fixed) | 4 | 1.14 | 1.00-1.31 | 0.059 | 0.348 |
|  | MR-Egger | 4 | / | / | 0.112* | / |
|  | Weighted median | 4 | 1.22 | 1.02-1.46 | 0.028 | / |
|  | Maximum-likelihood method | 4 | 1.15 | 0.99-1.32 | 0.067 | / |
|  | MR-PRESSO test | 4 | 1.14 | 0.99-1.32 | 0.170 | 0.351 |
| *Negativicutes* |  |  |  |  |  |  |
|  | Inverse-variance weighted (fixed) | 4 | 0.99 | 0.89-1.11 | 0.894 | 0.899 |
|  | MR-Egger | 4 | / | / | 0.669* | / |
|  | Weighted median | 4 | 1.00 | 0.88-1.13 | 0.951 | / |
|  | Maximum-likelihood method | 4 | 0.99 | 0.89-1.11 | 0.894 | / |
|  | MR-PRESSO test | 4 | 0.99 | 0.95-1.04 | 0.784 | 0.886 |
| *Verrucomicrobiae* |  |  |  |  |  |  |
|  | Inverse-variance weighted (fixed) | 4 | 0.97 | 0.85-1.11 | 0.703 | 0.266 |
|  | MR-Egger | 4 | / | / | 0.549* | / |
|  | Weighted median | 4 | 0.97 | 0.82-1.15 | 0.763 | / |
|  | Maximum-likelihood method | 4 | 0.97 | 0.83-1.14 | 0.735 | / |
|  | MR-PRESSO test | 4 | 0.97 | 0.84-1.14 | 0.762 | 0.307 |
| **Family** |  |  |  |  |  |  |
| *Acidaminococcaceae* |  |  |  |  |  |  |
|  | Inverse-variance weighted (fixed) | 4 | 0.98 | 0.86-1.11 | 0.716 | 0.998 |
|  | MR-Egger | 4 | / | / | 0.984* | / |
|  | Weighted median | 4 | 0.98 | 0.84-1.14 | 0.790 | / |
|  | Maximum-likelihood method | 4 | 0.98 | 0.86-1.11 | 0.716 | / |
|  | MR-PRESSO test | 4 | 0.98 | 0.96-0.99 | 0.049 | 0.999 |
| *Actinomycetaceae* |  |  |  |  |  |  |
|  | Inverse-variance weighted (fixed) | 4 | 1.02 | 0.86-1.21 | 0.792 | 0.608 |
|  | MR-Egger | 4 | / | / | 0.792* | / |
|  | Weighted median | 4 | 1.06 | 0.86-1.29 | 0.601 | / |
|  | Maximum-likelihood method | 4 | 1.02 | 0.86-1.21 | 0.790 | / |
|  | MR-PRESSO test | 4 | 1.02 | 0.90-1.17 | 0.758 | 0.651 |
| *Alcaligenaceae* |  |  |  |  |  |  |
|  | Inverse-variance weighted (fixed) | 4 | 0.95 | 0.85-1.06 | 0.360 | 0.974 |
|  | MR-Egger | 4 | / | / | 0.675* | / |
|  | Weighted median | 4 | 0.95 | 0.83-1.08 | 0.437 | / |
|  | Maximum-likelihood method | 4 | 0.95 | 0.85-1.06 | 0.362 | / |
|  | MR-PRESSO test | 4 | 0.95 | 0.92-0.98 | 0.043 | 0.967 |
| *Bacteroidaceae* |  |  |  |  |  |  |
|  | Inverse-variance weighted (fixed) | 4 | 0.93 | 0.84-1.04 | 0.218 | 0.150 |
|  | MR-Egger | 4 | / | / | 0.054* | / |
|  | Weighted median | 4 | 0.91 | 0.79-1.05 | 0.199 | / |
|  | Maximum-likelihood method | 4 | 0.93 | 0.81-1.08 | 0.340 | / |
|  | MR-PRESSO test | 4 | 0.93 | 0.81-1.08 | 0.423 | 0.133 |
| *BacteroidalesS24* |  |  |  |  |  |  |
|  | Inverse-variance weighted (fixed) | 4 | 0.97 | 0.83-1.15 | 0.759 | 0.423 |
|  | MR-Egger | 4 | / | / | 0.891* | / |
|  | Weighted median | 4 | 0.92 | 0.76-1.13 | 0.437 | / |
|  | Maximum-likelihood method | 4 | 0.97 | 0.83-1.15 | 0.757 | / |
|  | MR-PRESSO test | 4 | 0.97 | 0.83-1.14 | 0.772 | 0.474 |
| *Bifidobacteriaceae* |  |  |  |  |  |  |
|  | Inverse-variance weighted (fixed) | 4 | 1.02 | 0.90-1.15 | 0.783 | 0.387 |
|  | MR-Egger | 4 | / | / | 0.302* | / |
|  | Weighted median | 4 | 1.03 | 0.89-1.20 | 0.681 | / |
|  | Maximum-likelihood method | 4 | 1.02 | 0.90-1.15 | 0.781 | / |
|  | MR-PRESSO test | 4 | 1.02 | 0.90-1.15 | 0.802 | 0.343 |
| *Christensenellaceae* |  |  |  |  |  |  |
|  | Inverse-variance weighted (fixed) | 4 | 1.02 | 0.91-1.14 | 0.751 | 0.208 |
|  | MR-Egger | 4 | / | / | 0.061* | / |
|  | Weighted median | 4 | 1.09 | 0.94-1.26 | 0.246 | / |
|  | Maximum-likelihood method | 4 | 1.02 | 0.88-1.17 | 0.791 | / |
|  | MR-PRESSO test | 4 | 1.02 | 0.89-1.17 | 0.813 | 0.206 |
| *Clostridiaceae1* |  |  |  |  |  |  |
|  | Inverse-variance weighted (fixed) | 4 | 1.00 | 0.88-1.13 | 0.956 | 0.621 |
|  | MR-Egger | 4 | / | / | 0.964* | / |
|  | Weighted median | 4 | 0.97 | 0.84-1.12 | 0.659 | / |
|  | Maximum-likelihood method | 4 | 1.00 | 0.88-1.13 | 0.956 | / |
|  | MR-PRESSO test | 4 | 1.00 | 0.91-1.09 | 0.947 | 0.673 |
| *ClostridialesvadinBB60group* |  |  |  |  |  |  |
|  | Inverse-variance weighted (fixed) | 4 | 1.10 | 0.95-1.26 | 0.192 | 0.264 |
|  | MR-Egger | 4 | / | / | 0.583* | / |
|  | Weighted median | 4 | 1.05 | 0.87-1.26 | 0.607 | / |
|  | Maximum-likelihood method | 4 | 1.10 | 0.93-1.31 | 0.249 | / |
|  | MR-PRESSO test | 4 | 1.10 | 0.93-1.29 | 0.339 | 0.255 |
| *Coriobacteriaceae* |  |  |  |  |  |  |
|  | Inverse-variance weighted (fixed) | 4 | 0.99 | 0.88-1.10 | 0.796 | 0.373 |
|  | MR-Egger | 4 | / | / | 0.922* | / |
|  | Weighted median | 4 | 0.96 | 0.83-1.10 | 0.524 | / |
|  | Maximum-likelihood method | 4 | 0.99 | 0.88-1.10 | 0.797 | / |
|  | MR-PRESSO test | 4 | 0.99 | 0.88-1.10 | 0.816 | 0.431 |
| *Defluviitaleaceae* |  |  |  |  |  |  |
|  | Inverse-variance weighted (fixed) | 4 | 1.05 | 0.89-1.23 | 0.583 | 0.190 |
|  | MR-Egger | 4 | / | / | 0.483* | / |
|  | Weighted median | 4 | 1.04 | 0.84-1.29 | 0.687 | / |
|  | Maximum-likelihood method | 4 | 1.05 | 0.85-1.29 | 0.656 | / |
|  | MR-PRESSO test | 4 | 1.05 | 0.85-1.28 | 0.693 | 0.216 |
| *Desulfovibrionaceae* |  |  |  |  |  |  |
|  | Inverse-variance weighted (fixed) | 4 | 1.01 | 0.90-1.13 | 0.892 | 0.766 |
|  | MR-Egger | 4 | / | / | 0.963* | / |
|  | Weighted median | 4 | 1.02 | 0.89-1.17 | 0.765 | / |
|  | Maximum-likelihood method | 4 | 1.01 | 0.90-1.14 | 0.892 | / |
|  | MR-PRESSO test | 4 | 1.01 | 0.94-1.08 | 0.841 | 0.798 |
| *Enterobacteriaceae* |  |  |  |  |  |  |
|  | Inverse-variance weighted (fixed) | 4 | 0.92 | 0.81-1.04 | 0.187 | 0.638 |
|  | MR-Egger | 4 | / | / | 0.789* | / |
|  | Weighted median | 4 | 0.91 | 0.78-1.05 | 0.199 | / |
|  | Maximum-likelihood method | 4 | 0.92 | 0.81-1.04 | 0.187 | / |
|  | MR-PRESSO test | 4 | 0.92 | 0.84-1.01 | 0.177 | 0.689 |
| *Erysipelotrichaceae* |  |  |  |  |  |  |
|  | Inverse-variance weighted (fixed) | 4 | 0.96 | 0.86-1.06 | 0.408 | 0.428 |
|  | MR-Egger | 4 | / | / | 0.141* | / |
|  | Weighted median | 4 | 0.91 | 0.79-1.05 | 0.190 | / |
|  | Maximum-likelihood method | 4 | 0.95 | 0.86-1.06 | 0.402 | / |
|  | MR-PRESSO test | 4 | 0.96 | 0.86-1.06 | 0.453 | 0.415 |
| *FamilyXI* |  |  |  |  |  |  |
|  | Inverse-variance weighted (fixed) | 4 | 1.00 | 0.76-1.32 | 0.976 | 0.410 |
|  | MR-Egger | 4 | / | / | 0.188* | / |
|  | Weighted median | 4 | 1.12 | 0.81-1.55 | 0.493 | / |
|  | Maximum-likelihood method | 4 | 1.00 | 0.76-1.33 | 0.975 | / |
|  | MR-PRESSO test | 4 | 1.00 | 0.77-1.31 | 0.977 | 0.439 |
| *FamilyXIII* |  |  |  |  |  |  |
|  | Inverse-variance weighted (fixed) | 4 | 0.98 | 0.87-1.10 | 0.731 | 0.312 |
|  | MR-Egger | 4 | / | / | 0.062* | / |
|  | Weighted median | 4 | 1.06 | 0.91-1.24 | 0.476 | / |
|  | Maximum-likelihood method | 4 | 0.98 | 0.86-1.11 | 0.747 | / |
|  | MR-PRESSO test | 4 | 0.98 | 0.86-1.11 | 0.773 | 0.228 |
| *Lactobacillaceae* |  |  |  |  |  |  |
|  | Inverse-variance weighted (fixed) | 5 | 0.92 | 0.79-1.06 | 0.258 | 0.001 |
|  | Weighted median | 5 | 0.88 | 0.72-1.08 | 0.229 | / |
|  | Maximum-likelihood method | 5 | 0.91 | 0.65-1.27 | 0.560 | / |
|  | MR-PRESSO test | 4 | 0.92 | 0.67-1.26 | 0.622 | 0.002 |
| *Methanobacteriaceae* |  |  |  |  |  |  |
|  | Inverse-variance weighted (fixed) | 4 | 0.89 | 0.68-1.16 | 0.372 | 0.144 |
|  | MR-Egger | 4 | / | / | 0.888* | / |
|  | Weighted median | 4 | 0.98 | 0.70-1.38 | 0.922 | / |
|  | Maximum-likelihood method | 4 | 0.88 | 0.61-1.27 | 0.492 | / |
|  | MR-PRESSO test | 4 | 0.89 | 0.62-1.27 | 0.554 | 0.157 |
| *Pasteurellaceae* |  |  |  |  |  |  |
|  | Inverse-variance weighted (fixed) | 4 | 0.92 | 0.79-1.07 | 0.276 | 0.575 |
|  | MR-Egger | 4 | / | / | 0.173* | / |
|  | Weighted median | 4 | 0.92 | 0.77-1.11 | 0.408 | / |
|  | Maximum-likelihood method | 4 | 0.92 | 0.79-1.07 | 0.276 | / |
|  | MR-PRESSO test | 4 | 0.92 | 0.82-1.04 | 0.273 | 0.519 |
| *Peptococcaceae* |  |  |  |  |  |  |
|  | Inverse-variance weighted (fixed) | 4 | 1.02 | 0.89-1.18 | 0.748 | 0.013 |
|  | MR-Egger | 4 | / | / | 0.583* | / |
|  | Weighted median | 4 | 1.00 | 0.82-1.21 | 0.982 | / |
|  | Maximum-likelihood method | 4 | 1.03 | 0.77-1.36 | 0.858 | / |
|  | MR-PRESSO test | 3 | 1.02 | 0.78-1.34 | 0.876 | 0.028 |
| *Peptostreptococcaceae* |  |  |  |  |  |  |
|  | Inverse-variance weighted (fixed) | 5 | 1.02 | 0.92-1.13 | 0.721 | 0.971 |
|  | MR-Egger | 5 | / | / | 0.876* | / |
|  | Weighted median | 5 | 1.04 | 0.92-1.17 | 0.521 | / |
|  | Maximum-likelihood method | 5 | 1.02 | 0.92-1.13 | 0.720 | / |
|  | MR-PRESSO test | 5 | 1.02 | 0.98-1.06 | 0.381 | 0.973 |
| *Porphyromonadaceae* |  |  |  |  |  |  |
|  | Inverse-variance weighted (fixed) | 4 | 0.91 | 0.82-1.02 | 0.107 | 0.263 |
|  | MR-Egger | 4 | / | / | 0.298* | / |
|  | Weighted median | 4 | 0.91 | 0.79-1.05 | 0.185 | / |
|  | Maximum-likelihood method | 4 | 0.91 | 0.80-1.03 | 0.152 | / |
|  | MR-PRESSO test | 4 | 0.91 | 0.81-1.04 | 0.256 | 0.256 |
| *Rhodospirillaceae* |  |  |  |  |  |  |
|  | Inverse-variance weighted (fixed) | 5 | 1.10 | 0.96-1.26 | 0.159 | 0.845 |
|  | MR-Egger | 5 | / | / | 0.532* | / |
|  | Weighted median | 5 | 1.09 | 0.93-1.29 | 0.294 | / |
|  | Maximum-likelihood method | 5 | 1.10 | 0.96-1.27 | 0.160 | / |
|  | MR-PRESSO test | 5 | 1.10 | 1.02-1.19 | 0.075 | 0.865 |
| *Rikenellaceae* |  |  |  |  |  |  |
|  | Inverse-variance weighted (fixed) | 4 | 0.86 | 0.76-0.97 | 0.013 | 0.301 |
|  | MR-Egger | 4 | / | / | 0.668* | / |
|  | Weighted median | 4 | 0.81 | 0.70-0.95 | 0.008 | / |
|  | Maximum-likelihood method | 4 | 0.85 | 0.74-0.98 | 0.022 | / |
|  | MR-PRESSO test | 4 | 0.86 | 0.75-0.98 | 0.109 | 0.353 |
| *Streptococcaceae* |  |  |  |  |  |  |
|  | Inverse-variance weighted (fixed) | 4 | 1.12 | 1.00-1.25 | 0.058 | 0.246 |
|  | MR-Egger | 4 | / | / | 0.141* | / |
|  | Weighted median | 4 | 1.08 | 0.94-1.26 | 0.281 | / |
|  | Maximum-likelihood method | 4 | 1.12 | 0.98-1.28 | 0.102 | / |
|  | MR-PRESSO test | 4 | 1.12 | 0.98-1.27 | 0.206 | 0.277 |
| *Verrucomicrobiaceae* |  |  |  |  |  |  |
|  | Inverse-variance weighted (fixed) | 5 | 0.97 | 0.86-1.09 | 0.578 | 0.410 |
|  | MR-Egger | 5 | / | / | 0.602* | / |
|  | Weighted median | 5 | 0.95 | 0.82-1.10 | 0.499 | / |
|  | Maximum-likelihood method | 5 | 0.97 | 0.86-1.09 | 0.573 | / |
|  | MR-PRESSO test | 5 | 0.97 | 0.86-1.09 | 0.606 | 0.470 |
| *Victivallaceae* |  |  |  |  |  |  |
|  | Inverse-variance weighted (fixed) | 4 | 1.28 | 1.00-1.65 | 0.054 | 0.871 |
|  | MR-Egger | 4 | / | / | 0.607* | / |
|  | Weighted median | 4 | 1.22 | 0.91-1.65 | 0.187 | / |
|  | Maximum-likelihood method | 4 | 1.28 | 0.99-1.66 | 0.058 | / |
|  | MR-PRESSO test | 4 | 1.28 | 1.13-1.45 | 0.029 | 0.866 |
| *Lachnospiraceae* |  |  |  |  |  |  |
|  | Inverse-variance weighted (fixed) | 3 | 1.07 | 0.94-1.20 | 0.301 | 0.482 |
|  | MR-Egger | 3 | / | / | 0.349* | / |
|  | Weighted median | 3 | 1.07 | 0.93-1.23 | 0.324 | / |
|  | Maximum-likelihood method | 3 | 1.07 | 0.94-1.21 | 0.302 | / |
| *Oxalobacteraceae* |  |  |  |  |  |  |
|  | Inverse-variance weighted (fixed) | 3 | 0.70 | 0.56-0.88 | 0.002 | 0.600 |
|  | MR-Egger | 3 | / | / | 0.404* | / |
|  | Weighted median | 3 | 0.68 | 0.52-0.89 | 0.006 | / |
|  | Maximum-likelihood method | 3 | 0.70 | 0.55-0.89 | 0.004 | / |
| *Prevotellaceae* |  |  |  |  |  |  |
|  | Inverse-variance weighted (fixed) | 3 | 1.06 | 0.93-1.21 | 0.381 | 0.190 |
|  | MR-Egger | 3 | / | / | 0.090* | / |
|  | Weighted median | 3 | 1.01 | 0.86-1.19 | 0.916 | / |
|  | Maximum-likelihood method | 3 | 1.06 | 0.93-1.22 | 0.369 | / |
| *Ruminococcaceae* |  |  |  |  |  |  |
|  | Inverse-variance weighted (fixed) | 3 | 0.96 | 0.85-1.08 | 0.523 | 0.397 |
|  | MR-Egger | 3 | / | / | 0.196* | / |
|  | Weighted median | 3 | 1.00 | 0.87-1.16 | 0.975 | / |
|  | Maximum-likelihood method | 3 | 0.96 | 0.85-1.08 | 0.519 | / |
| *Veillonellaceae* |  |  |  |  |  |  |
|  | Inverse-variance weighted (fixed) | 3 | 0.96 | 0.84-1.09 | 0.528 | 0.585 |
|  | MR-Egger | 3 | / | / | 0.312* | / |
|  | Weighted median | 3 | 0.98 | 0.85-1.14 | 0.802 | / |
|  | Maximum-likelihood method | 3 | 0.96 | 0.84-1.09 | 0.527 | / |
| **Genus** |  |  |  |  |  |  |
| *Actinomyces* |  |  |  |  |  |  |
|  | Inverse-variance weighted (fixed) | 4 | 1.05 | 0.89-1.25 | 0.556 | 0.552 |
|  | MR-Egger | 4 | / | / | 0.905* | / |
|  | Weighted median | 4 | 1.06 | 0.86-1.30 | 0.581 | / |
|  | Maximum-likelihood method | 4 | 1.05 | 0.89-1.25 | 0.553 | / |
|  | MR-PRESSO test | 4 | 1.05 | 0.91-1.22 | 0.532 | 0.607 |
| *Adlercreutzia* |  |  |  |  |  |  |
|  | Inverse-variance weighted (fixed) | 4 | 0.85 | 0.72-1.00 | 0.056 | 0.474 |
|  | MR-Egger | 4 | / | / | 0.626* | / |
|  | Weighted median | 4 | 0.87 | 0.71-1.07 | 0.187 | / |
|  | Maximum-likelihood method | 4 | 0.85 | 0.72-1.01 | 0.058 | / |
|  | MR-PRESSO test | 4 | 0.85 | 0.73-0.99 | 0.128 | 0.535 |
| *Akkermansia* |  |  |  |  |  |  |
|  | Inverse-variance weighted (fixed) | 4 | 0.98 | 0.85-1.12 | 0.712 | 0.256 |
|  | MR-Egger | 4 | / | / | 0.562* | / |
|  | Weighted median | 4 | 0.97 | 0.82-1.15 | 0.755 | / |
|  | Maximum-likelihood method | 4 | 0.97 | 0.83-1.14 | 0.746 | / |
|  | MR-PRESSO test | 4 | 0.98 | 0.83-1.14 | 0.772 | 0.281 |
| *Alistipes* |  |  |  |  |  |  |
|  | Inverse-variance weighted (fixed) | 4 | 0.85 | 0.76-0.95 | 0.005 | 0.667 |
|  | MR-Egger | 4 | / | / | 0.659* | / |
|  | Weighted median | 4 | 0.84 | 0.73-0.96 | 0.012 | / |
|  | Maximum-likelihood method | 4 | 0.85 | 0.76-0.96 | 0.006 | / |
|  | MR-PRESSO test | 4 | 0.85 | 0.79-0.92 | 0.03 | 0.683 |
| *Allisonella* |  |  |  |  |  |  |
|  | Inverse-variance weighted (fixed) | 3 | 1.08 | 0.78-1.49 | 0.649 | 0.143 |
|  | MR-Egger | / | / | 0.03-1.13 | 0.053* | / |
|  | Weighted median | 3 | 0.99 | 0.64-1.55 | 0.977 | / |
|  | Maximum-likelihood method | 3 | 1.08 | 0.77-1.52 | 0.64 | / |
| *Alloprevotella* |  |  |  |  |  |  |
|  | Inverse-variance weighted (fixed) | 3 | 0.97 | 0.70-1.35 | 0.862 | 0.026 |
|  | MR-Egger | 3 | / | / | 0.968* | / |
|  | Weighted median | 3 | 0.9 | 0.56-1.45 | 0.658 | / |
|  | Maximum-likelihood method | 3 | 0.97 | 0.68-1.37 | 0.854 | / |
| *Anaerofilum* |  |  |  |  |  |  |
|  | Inverse-variance weighted (fixed) | 4 | 0.89 | 0.72-1.09 | 0.246 | 0.975 |
|  | MR-Egger | 4 | / | / | 0.795* | / |
|  | Weighted median | 4 | 0.89 | 0.70-1.13 | 0.34 | / |
|  | Maximum-likelihood method | 4 | 0.88 | 0.72-1.09 | 0.249 | / |
|  | MR-PRESSO test | 4 | 0.89 | 0.84-0.94 | 0.023 | 0.971 |
| *Anaerostipes* |  |  |  |  |  |  |
|  | Inverse-variance weighted (fixed) | 4 | 1.07 | 0.96-1.20 | 0.237 | 0.436 |
|  | MR-Egger | 4 | / | / | 0.113* | / |
|  | Weighted median | 4 | 1.03 | 0.90-1.19 | 0.658 | / |
|  | Maximum-likelihood method | 4 | 1.07 | 0.96-1.20 | 0.235 | / |
|  | MR-PRESSO test | 4 | 1.07 | 0.96-1.19 | 0.303 | 0.396 |
| *Anaerotruncus* |  |  |  |  |  |  |
|  | Inverse-variance weighted (fixed) | 4 | 1.02 | 0.91-1.14 | 0.754 | 0.612 |
|  | MR-Egger | 4 | / | / | 0.95* | / |
|  | Weighted median | 4 | 0.98 | 0.86-1.13 | 0.812 | / |
|  | Maximum-likelihood method | 4 | 1.02 | 0.91-1.14 | 0.753 | / |
|  | MR-PRESSO test | 4 | 1.02 | 0.93-1.11 | 0.714 | 0.645 |
| *Bacteroides* |  |  |  |  |  |  |
|  | Inverse-variance weighted (fixed) | 4 | 0.93 | 0.84-1.04 | 0.218 | 0.15 |
|  | MR-Egger | 4 | / | / | 0.054* | / |
|  | Weighted median | 4 | 0.91 | 0.79-1.05 | 0.199 | / |
|  | Maximum-likelihood method | 4 | 0.93 | 0.81-1.08 | 0.34 | / |
|  | MR-PRESSO test | 4 | 0.93 | 0.81-1.08 | 0.423 | 0.152 |
| *Barnesiella* |  |  |  |  |  |  |
|  | Inverse-variance weighted (fixed) | 4 | 0.89 | 0.79-1.01 | 0.066 | 0.483 |
|  | MR-Egger | 4 | / | / | 0.755* | / |
|  | Weighted median | 4 | 0.91 | 0.78-1.06 | 0.223 | / |
|  | Maximum-likelihood method | 4 | 0.89 | 0.78-1.01 | 0.069 | / |
|  | MR-PRESSO test | 4 | 0.89 | 0.80-1.00 | 0.135 | 0.515 |
| *Bifidobacterium* |  |  |  |  |  |  |
|  | Inverse-variance weighted (fixed) | 4 | 1.01 | 0.90-1.14 | 0.836 | 0.459 |
|  | MR-Egger | 4 | / | / | 0.405* | / |
|  | Weighted median | 4 | 1.01 | 0.87-1.17 | 0.888 | / |
|  | Maximum-likelihood method | 4 | 1.01 | 0.90-1.14 | 0.834 | / |
|  | MR-PRESSO test | 4 | 1.01 | 0.91-1.13 | 0.838 | 0.4 |
| *Bilophila* |  |  |  |  |  |  |
|  | Inverse-variance weighted (fixed) | 4 | 0.96 | 0.84-1.09 | 0.496 | 0.186 |
|  | MR-Egger | 4 | / | / | 0.784* | / |
|  | Weighted median | 4 | 0.97 | 0.83-1.15 | 0.763 | / |
|  | Maximum-likelihood method | 4 | 0.95 | 0.81-1.13 | 0.583 | / |
|  | MR-PRESSO test | 4 | 0.96 | 0.81-1.12 | 0.628 | 0.239 |
| *Blautia* |  |  |  |  |  |  |
|  | Inverse-variance weighted (fixed) | 4 | 0.96 | 0.86-1.07 | 0.443 | 0.403 |
|  | MR-Egger | 4 | / | / | 0.181* | / |
|  | Weighted median | 4 | 0.93 | 0.81-1.07 | 0.301 | / |
|  | Maximum-likelihood method | 4 | 0.96 | 0.86-1.07 | 0.437 | / |
|  | MR-PRESSO test | 4 | 0.96 | 0.86-1.07 | 0.494 | 0.404 |
| *Butyricicoccus* |  |  |  |  |  |  |
|  | Inverse-variance weighted (fixed) | 4 | 1.03 | 0.92-1.16 | 0.56 | 0.842 |
|  | MR-Egger | 4 | / | / | 0.835* | / |
|  | Weighted median | 4 | 1.05 | 0.92-1.19 | 0.474 | / |
|  | Maximum-likelihood method | 4 | 1.03 | 0.92-1.16 | 0.559 | / |
|  | MR-PRESSO test | 4 | 1.03 | 0.97-1.10 | 0.348 | 0.866 |
| *Butyricimonas* |  |  |  |  |  |  |
|  | Inverse-variance weighted (fixed) | 4 | 0.92 | 0.80-1.06 | 0.238 | 0.01 |
|  | MR-Egger | 4 | / | / | 0.095* | / |
|  | Weighted median | 4 | 0.82 | 0.66-1.03 | 0.086 | / |
|  | Maximum-likelihood method | 4 | 0.91 | 0.67-1.23 | 0.52 | / |
|  | MR-PRESSO test | 3 | 0.92 | 0.70-1.21 | 0.588 | 0.009 |
| *Butyrivibrio* |  |  |  |  |  |  |
|  | Inverse-variance weighted (fixed) | 4 | 1.09 | 0.85-1.39 | 0.506 | 0.015 |
|  | MR-Egger | 4 | / | / | 0.004* | / |
|  | Weighted median | 4 | 1.08 | 0.74-1.56 | 0.686 | / |
|  | Maximum-likelihood method | 4 | 1.1 | 0.67-1.81 | 0.705 | / |
|  | MR-PRESSO test | 3 | 1.09 | 0.69-1.72 | 0.745 | 0.01 |
| *CandidatusSoleaferrea* |  |  |  |  |  |  |
|  | Inverse-variance weighted (fixed) | 4 | 0.93 | 0.77-1.12 | 0.426 | 0.074 |
|  | MR-Egger | 4 | / | / | 0.222* | / |
|  | Weighted median | 4 | 1 | 0.78-1.29 | 0.983 | / |
|  | Maximum-likelihood method | 4 | 0.92 | 0.69-1.24 | 0.588 | / |
|  | MR-PRESSO test | 4 | 0.93 | 0.70-1.23 | 0.637 | 0.11 |
| *Catenibacterium* |  |  |  |  |  |  |
|  | Inverse-variance weighted (fixed) | 3 | 0.96 | 0.70-1.32 | 0.794 | 0.289 |
|  | MR-Egger | 3 | / | / | 0.843* | / |
|  | Weighted median | 3 | 0.96 | 0.64-1.44 | 0.832 | / |
|  | Maximum-likelihood method | 3 | 0.96 | 0.69-1.32 | 0.79 | / |
| *ChristensenellaceaeR* |  |  |  |  |  |  |
|  | Inverse-variance weighted (fixed) | 4 | 1.03 | 0.92-1.15 | 0.615 | 0.385 |
|  | MR-Egger | 4 | / | / | 0.203* | / |
|  | Weighted median | 4 | 1.08 | 0.93-1.24 | 0.317 | / |
|  | Maximum-likelihood method | 4 | 1.03 | 0.92-1.16 | 0.612 | / |
|  | MR-PRESSO test | 4 | 1.03 | 0.92-1.15 | 0.652 | 0.434 |
| *Clostridiuminnocuumgroup* |  |  |  |  |  |  |
|  | Inverse-variance weighted (fixed) | 3 | 0.97 | 0.76-1.25 | 0.812 | 0.051 |
|  | MR-Egger | 3 | / | / | 0.038* | / |
|  | Weighted median | 3 | 0.83 | 0.59-1.16 | 0.264 | / |
|  | Maximum-likelihood method | 3 | 0.97 | 0.75-1.25 | 0.804 | / |
| *Clostridiumsensustricto1* |  |  |  |  |  |  |
|  | Inverse-variance weighted (fixed) | 4 | 0.99 | 0.88-1.12 | 0.903 | 0.681 |
|  | MR-Egger | 4 | / | / | 0.938* | / |
|  | Weighted median | 4 | 0.96 | 0.83-1.11 | 0.606 | / |
|  | Maximum-likelihood method | 4 | 0.99 | 0.88-1.12 | 0.902 | / |
|  | MR-PRESSO test | 4 | 0.99 | 0.91-1.08 | 0.874 | 0.719 |
| *Collinsella* |  |  |  |  |  |  |
|  | Inverse-variance weighted (fixed) | 4 | 0.97 | 0.86-1.09 | 0.593 | 0.488 |
|  | MR-Egger | 4 | / | / | 0.493* | / |
|  | Weighted median | 4 | 0.99 | 0.86-1.14 | 0.897 | / |
|  | Maximum-likelihood method | 4 | 0.97 | 0.85-1.09 | 0.589 | / |
|  | MR-PRESSO test | 4 | 0.97 | 0.87-1.08 | 0.594 | 0.493 |
| *Coprobacter* |  |  |  |  |  |  |
|  | Inverse-variance weighted (fixed) | 4 | 0.92 | 0.77-1.09 | 0.328 | 0.634 |
|  | MR-Egger | 4 | / | / | 0.272* | / |
|  | Weighted median | 4 | 0.88 | 0.71-1.09 | 0.249 | / |
|  | Maximum-likelihood method | 4 | 0.91 | 0.76-1.09 | 0.327 | / |
|  | MR-PRESSO test | 4 | 0.92 | 0.80-1.05 | 0.286 | 0.575 |
| *Coprococcus1* |  |  |  |  |  |  |
|  | Inverse-variance weighted (fixed) | 4 | 1.03 | 0.92-1.15 | 0.59 | 0.407 |
|  | MR-Egger | 4 | / | / | 0.541* | / |
|  | Weighted median | 4 | 1.01 | 0.87-1.16 | 0.911 | / |
|  | Maximum-likelihood method | 4 | 1.03 | 0.92-1.16 | 0.585 | / |
|  | MR-PRESSO test | 4 | 1.03 | 0.92-1.15 | 0.622 | 0.38 |
| *Coprococcus2* |  |  |  |  |  |  |
|  | Inverse-variance weighted (fixed) | 4 | 1.13 | 0.99-1.30 | 0.075 | 0.871 |
|  | MR-Egger | 4 | / | / | 0.771* | / |
|  | Weighted median | 4 | 1.12 | 0.95-1.32 | 0.166 | / |
|  | Maximum-likelihood method | 4 | 1.13 | 0.99-1.30 | 0.079 | / |
|  | MR-PRESSO test | 4 | 1.13 | 1.06-1.21 | 0.035 | 0.89 |
| *Coprococcus3* |  |  |  |  |  |  |
|  | Inverse-variance weighted (fixed) | 4 | 1.02 | 0.91-1.15 | 0.706 | 0.53 |
|  | MR-Egger | 4 | / | / | 0.833* | / |
|  | Weighted median | 4 | 1.05 | 0.91-1.21 | 0.493 | / |
|  | Maximum-likelihood method | 4 | 1.02 | 0.91-1.15 | 0.703 | / |
|  | MR-PRESSO test | 4 | 1.02 | 0.93-1.13 | 0.691 | 0.554 |
| *DefluviitaleaceaeUCG011* |  |  |  |  |  |  |
|  | Inverse-variance weighted (fixed) | 4 | 1.04 | 0.89-1.23 | 0.613 | 0.201 |
|  | MR-Egger | 4 | / | / | 0.525* | / |
|  | Weighted median | 4 | 1.04 | 0.84-1.29 | 0.691 | / |
|  | Maximum-likelihood method | 4 | 1.04 | 0.85-1.28 | 0.677 | / |
|  | MR-PRESSO test | 4 | 1.04 | 0.85-1.28 | 0.711 | 0.243 |
| *Desulfovibrio* |  |  |  |  |  |  |
|  | Inverse-variance weighted (fixed) | 4 | 1.08 | 0.93-1.26 | 0.32 | 0.568 |
|  | MR-Egger | 4 | / | / | 0.767* | / |
|  | Weighted median | 4 | 1.02 | 0.84-1.23 | 0.863 | / |
|  | Maximum-likelihood method | 4 | 1.08 | 0.93-1.26 | 0.318 | / |
|  | MR-PRESSO test | 4 | 1.08 | 0.95-1.22 | 0.313 | 0.576 |
| *Dialister* |  |  |  |  |  |  |
|  | Inverse-variance weighted (fixed) | 4 | 1.04 | 0.91-1.18 | 0.59 | 0.354 |
|  | MR-Egger | 4 | / | / | 0.430* | / |
|  | Weighted median | 4 | 1.05 | 0.89-1.24 | 0.583 | / |
|  | Maximum-likelihood method | 4 | 1.04 | 0.90-1.20 | 0.599 | / |
|  | MR-PRESSO test | 4 | 1.04 | 0.90-1.19 | 0.64 | 0.374 |
| *Dorea* |  |  |  |  |  |  |
|  | Inverse-variance weighted (fixed) | 4 | 0.99 | 0.88-1.10 | 0.806 | 0.283 |
|  | MR-Egger | 4 | / | / | 0.372* | / |
|  | Weighted median | 4 | 0.97 | 0.84-1.12 | 0.703 | / |
|  | Maximum-likelihood method | 4 | 0.99 | 0.87-1.12 | 0.824 | / |
|  | MR-PRESSO test | 4 | 0.99 | 0.87-1.12 | 0.841 | 0.32 |
| *Eggerthella* |  |  |  |  |  |  |
|  | Inverse-variance weighted (fixed) | 4 | 0.82 | 0.67-1.01 | 0.057 | 0.808 |
|  | MR-Egger | 4 | / | / | 0.626* | / |
|  | Weighted median | 4 | 0.82 | 0.63-1.05 | 0.116 | / |
|  | Maximum-likelihood method | 4 | 0.82 | 0.66-1.01 | 0.061 | / |
|  | MR-PRESSO test | 4 | 0.82 | 0.73-0.92 | 0.044 | 0.826 |
| *Eisenbergiella* |  |  |  |  |  |  |
|  | Inverse-variance weighted (fixed) | 4 | 0.93 | 0.76-1.12 | 0.433 | 0.341 |
|  | MR-Egger | 4 | / | / | 0.878* | / |
|  | Weighted median | 4 | 0.87 | 0.68-1.10 | 0.237 | / |
|  | Maximum-likelihood method | 4 | 0.92 | 0.75-1.14 | 0.45 | / |
|  | MR-PRESSO test | 4 | 0.93 | 0.75-1.14 | 0.512 | 0.386 |
| *Enterorhabdus* |  |  |  |  |  |  |
|  | Inverse-variance weighted (fixed) | 4 | 1.03 | 0.87-1.22 | 0.721 | 0.964 |
|  | MR-Egger | 4 | / | / | 0.780* | / |
|  | Weighted median | 4 | 1.04 | 0.86-1.27 | 0.673 | / |
|  | Maximum-likelihood method | 4 | 1.03 | 0.87-1.22 | 0.721 | / |
|  | MR-PRESSO test | 4 | 1.03 | 0.98-1.09 | 0.324 | 0.964 |
| *Erysipelatoclostridium* |  |  |  |  |  |  |
|  | Inverse-variance weighted (fixed) | 4 | 0.93 | 0.80-1.07 | 0.312 | 0.129 |
|  | MR-Egger | 4 | / | / | 0.025* | / |
|  | Weighted median | 4 | 0.88 | 0.72-1.08 | 0.227 | / |
|  | Maximum-likelihood method | 4 | 0.92 | 0.75-1.14 | 0.445 | / |
|  | MR-PRESSO test | 4 | 0.93 | 0.76-1.14 | 0.515 | 0.106 |
| *ErysipelotrichaceaeUCG003* |  |  |  |  |  |  |
|  | Inverse-variance weighted (fixed) | 4 | 0.95 | 0.84-1.07 | 0.38 | 0.119 |
|  | MR-Egger | 4 | / | / | 0.351* | / |
|  | Weighted median | 4 | 0.92 | 0.79-1.09 | 0.333 | / |
|  | Maximum-likelihood method | 4 | 0.94 | 0.80-1.12 | 0.517 | / |
|  | MR-PRESSO test | 4 | 0.95 | 0.80-1.12 | 0.574 | 0.15 |
| *Escherichia.Shigella* |  |  |  |  |  |  |
|  | Inverse-variance weighted (fixed) | 4 | 0.94 | 0.83-1.07 | 0.374 | 0.343 |
|  | MR-Egger | 4 | / | / | 0.178* | / |
|  | Weighted median | 4 | 0.99 | 0.83-1.17 | 0.909 | / |
|  | Maximum-likelihood method | 4 | 0.94 | 0.81-1.08 | 0.393 | / |
|  | MR-PRESSO test | 4 | 0.94 | 0.82-1.08 | 0.461 | 0.344 |
| *Eubacteriumbrachygroup* |  |  |  |  |  |  |
|  | Inverse-variance weighted (fixed) | 4 | 1.04 | 0.82-1.31 | 0.764 | 0.189 |
|  | MR-Egger | 4 | / | / | 0.842* | / |
|  | Weighted median | 4 | 0.97 | 0.73-1.30 | 0.857 | / |
|  | Maximum-likelihood method | 4 | 1.04 | 0.77-1.41 | 0.808 | / |
|  | MR-PRESSO test | 4 | 1.04 | 0.77-1.39 | 0.827 | 0.248 |
| *Eubacteriumcoprostanoligenesgroup* |  |  |  |  |  |  |
|  | Inverse-variance weighted (fixed) | 4 | 0.98 | 0.88-1.10 | 0.742 | 0.815 |
|  | MR-Egger | 4 | / | / | 0.878* | / |
|  | Weighted median | 4 | 0.97 | 0.85-1.11 | 0.679 | / |
|  | Maximum-likelihood method | 4 | 0.98 | 0.88-1.10 | 0.741 | / |
|  | MR-PRESSO test | 4 | 0.98 | 0.92-1.04 | 0.599 | 0.822 |
| *Eubacteriumeligensgroup* |  |  |  |  |  |  |
|  | Inverse-variance weighted (fixed) | 4 | 0.97 | 0.86-1.09 | 0.607 | 0.393 |
|  | MR-Egger | 4 | / | / | 0.095* | / |
|  | Weighted median | 4 | 0.99 | 0.85-1.16 | 0.923 | / |
|  | Maximum-likelihood method | 4 | 0.97 | 0.86-1.09 | 0.602 | / |
|  | MR-PRESSO test | 4 | 0.97 | 0.86-1.09 | 0.642 | 0.35 |
| *Eubacteriumfissicatenagroup* |  |  |  |  |  |  |
|  | Inverse-variance weighted (fixed) | 4 | 1 | 0.79-1.26 | 0.99 | 0.192 |
|  | MR-Egger | 4 | / | / | 0.615* | / |
|  | Weighted median | 4 | 1.01 | 0.74-1.37 | 0.964 | / |
|  | Maximum-likelihood method | 4 | 1 | 0.74-1.35 | 0.992 | / |
|  | MR-PRESSO test | 4 | 1 | 0.75-1.34 | 0.992 | 0.235 |
| *Eubacteriumhalliigroup* |  | 0 |  |  |  |  |
|  | Inverse-variance weighted (fixed) | 4 | 0.99 | 0.89-1.11 | 0.904 | 0.052 |
|  | MR-Egger | 4 | / | / | 0.028* | / |
|  | Weighted median | 4 | 0.95 | 0.81-1.12 | 0.567 | / |
|  | Maximum-likelihood method | 4 | 0.99 | 0.82-1.20 | 0.937 | / |
|  | MR-PRESSO test | 3 | 0.99 | 0.83-1.19 | 0.945 | 0.048 |
| *Eubacteriumnodatumgroup* |  |  |  |  |  |  |
|  | Inverse-variance weighted (fixed) | 3 | 0.9 | 0.65-1.24 | 0.509 | 0.733 |
|  | MR-Egger | 3 | / | / | 0.649* | / |
|  | Weighted median | 3 | 0.83 | 0.57-1.22 | 0.353 | / |
|  | Maximum-likelihood method | 3 | 0.9 | 0.65-1.24 | 0.509 | / |
| *Eubacteriumoxidoreducensgroup* |  |  |  |  |  |  |
|  | Inverse-variance weighted (fixed) | 4 | 1.11 | 0.91-1.35 | 0.308 | 0.033 |
|  | MR-Egger | 4 | / | / | 0.768* | / |
|  | Weighted median | 4 | 1.22 | 0.94-1.59 | 0.129 | / |
|  | Maximum-likelihood method | 4 | 1.12 | 0.79-1.60 | 0.532 | / |
|  | MR-PRESSO test | 3 | 1.11 | 0.79-1.56 | 0.593 | 0.05 |
| *Eubacteriumrectalegroup* |  |  |  |  |  |  |
|  | Inverse-variance weighted (fixed) | 4 | 1 | 0.89-1.11 | 0.929 | 0.153 |
|  | MR-Egger | 4 | / | / | 0.922* | / |
|  | Weighted median | 4 | 1.06 | 0.92-1.21 | 0.439 | / |
|  | Maximum-likelihood method | 4 | 0.99 | 0.86-1.16 | 0.945 | / |
|  | MR-PRESSO test | 4 | 1 | 0.86-1.15 | 0.951 | 0.176 |
| *Eubacteriumruminantiumgroup* |  |  |  |  |  |  |
|  | Inverse-variance weighted (fixed) | 4 | 0.98 | 0.83-1.15 | 0.777 | 0.266 |
|  | MR-Egger | 4 | / | / | 0.564* | / |
|  | Weighted median | 4 | 1.01 | 0.82-1.24 | 0.96 | / |
|  | Maximum-likelihood method | 4 | 0.98 | 0.80-1.18 | 0.801 | / |
|  | MR-PRESSO test | 4 | 0.98 | 0.81-1.18 | 0.821 | 0.239 |
| *Eubacteriumventriosumgroup* |  |  |  |  |  |  |
|  | Inverse-variance weighted (fixed) | 4 | 0.95 | 0.84-1.07 | 0.387 | 0.28 |
|  | MR-Egger | 4 | / | / | 0.057* | / |
|  | Weighted median | 4 | 0.93 | 0.80-1.09 | 0.358 | / |
|  | Maximum-likelihood method | 4 | 0.95 | 0.83-1.08 | 0.432 | / |
|  | MR-PRESSO test | 4 | 0.95 | 0.83-1.08 | 0.5 | 0.245 |
| *Eubacteriumxylanophilumgroup* |  |  |  |  |  |  |
|  | Inverse-variance weighted (fixed) | 4 | 1.05 | 0.93-1.19 | 0.437 | 0.783 |
|  | MR-Egger | 4 | / | / | 0.696* | / |
|  | Weighted median | 4 | 1.03 | 0.89-1.20 | 0.688 | / |
|  | Maximum-likelihood method | 4 | 1.05 | 0.93-1.20 | 0.437 | / |
|  | MR-PRESSO test | 4 | 1.05 | 0.97-1.13 | 0.286 | 0.781 |
| *Faecalibacterium* |  |  |  |  |  |  |
|  | Inverse-variance weighted (fixed) | 4 | 0.98 | 0.88-1.09 | 0.657 | 0.464 |
|  | MR-Egger | 4 | / | / | 0.322* | / |
|  | Weighted median | 4 | 0.96 | 0.84-1.10 | 0.563 | / |
|  | Maximum-likelihood method | 4 | 0.98 | 0.87-1.09 | 0.654 | / |
|  | MR-PRESSO test | 4 | 0.98 | 0.88-1.08 | 0.664 | 0.393 |
| *FamilyXIIIAD3011group* |  |  |  |  |  |  |
|  | Inverse-variance weighted (fixed) | 4 | 0.98 | 0.86-1.10 | 0.691 | 0.996 |
|  | MR-Egger | 4 | / | / | 0.938* | / |
|  | Weighted median | 4 | 0.97 | 0.84-1.12 | 0.687 | / |
|  | Maximum-likelihood method | 4 | 0.98 | 0.86-1.10 | 0.691 | / |
|  | MR-PRESSO test | 4 | 0.98 | 0.96-0.99 | 0.068 | 0.995 |
| *FamilyXIIIUCG001* |  |  |  |  |  |  |
|  | Inverse-variance weighted (fixed) | 4 | 1 | 0.88-1.14 | 0.979 | 0.65 |
|  | MR-Egger | 4 | / | / | 0.791* | / |
|  | Weighted median | 4 | 1.04 | 0.89-1.22 | 0.591 | / |
|  | Maximum-likelihood method | 4 | 1 | 0.88-1.14 | 0.979 | / |
|  | MR-PRESSO test | 4 | 1 | 0.91-1.10 | 0.974 | 0.64 |
| *Flavonifractor* |  |  |  |  |  |  |
|  | Inverse-variance weighted (fixed) | 4 | 0.94 | 0.81-1.08 | 0.357 | 0.049 |
|  | MR-Egger | 4 | / | / | 0.264* | / |
|  | Weighted median | 4 | 0.91 | 0.75-1.10 | 0.314 | / |
|  | Maximum-likelihood method | 4 | 0.93 | 0.74-1.18 | 0.554 | / |
|  | MR-PRESSO test | 4 | 0.94 | 0.75-1.17 | 0.609 | 0.075 |
| *Fusicatenibacter* |  |  |  |  |  |  |
|  | Inverse-variance weighted (fixed) | 4 | 1.03 | 0.92-1.15 | 0.637 | 0.668 |
|  | MR-Egger | 4 | / | / | 0.865* | / |
|  | Weighted median | 4 | 1.04 | 0.91-1.19 | 0.578 | / |
|  | Maximum-likelihood method | 4 | 1.03 | 0.92-1.15 | 0.635 | / |
|  | MR-PRESSO test | 4 | 1.03 | 0.95-1.11 | 0.56 | 0.699 |
| *Gordonibacter* |  |  |  |  |  |  |
|  | Inverse-variance weighted (fixed) | 4 | 0.95 | 0.75-1.21 | 0.685 | 0.463 |
|  | MR-Egger | 4 | / | / | 0.824* | / |
|  | Weighted median | 4 | 0.93 | 0.69-1.25 | 0.641 | / |
|  | Maximum-likelihood method | 4 | 0.95 | 0.74-1.21 | 0.682 | / |
|  | MR-PRESSO test | 4 | 0.95 | 0.76-1.19 | 0.691 | 0.489 |
| *Haemophilus* |  |  |  |  |  |  |
|  | Inverse-variance weighted (fixed) | 4 | 0.92 | 0.79-1.07 | 0.279 | 0.466 |
|  | MR-Egger | 4 | / | / | 0.191* | / |
|  | Weighted median | 4 | 0.93 | 0.77-1.13 | 0.485 | / |
|  | Maximum-likelihood method | 4 | 0.92 | 0.79-1.07 | 0.277 | / |
|  | MR-PRESSO test | 4 | 0.92 | 0.80-1.06 | 0.325 | 0.434 |
| *Holdemanella* |  |  |  |  |  |  |
|  | Inverse-variance weighted (fixed) | 4 | 1.04 | 0.88-1.23 | 0.618 | 0.475 |
|  | MR-Egger | 4 | / | / | 0.893* | / |
|  | Weighted median | 4 | 1 | 0.81-1.23 | 0.989 | / |
|  | Maximum-likelihood method | 4 | 1.04 | 0.88-1.24 | 0.615 | / |
|  | MR-PRESSO test | 4 | 1.04 | 0.90-1.21 | 0.623 | 0.478 |
| *Holdemania* |  |  |  |  |  |  |
|  | Inverse-variance weighted (fixed) | 4 | 1.07 | 0.92-1.25 | 0.355 | 0.392 |
|  | MR-Egger | 4 | / | / | 0.691* | / |
|  | Weighted median | 4 | 1.13 | 0.93-1.37 | 0.222 | / |
|  | Maximum-likelihood method | 4 | 1.08 | 0.92-1.26 | 0.348 | / |
|  | MR-PRESSO test | 4 | 1.07 | 0.92-1.25 | 0.423 | 0.38 |
| *Howardella* |  |  |  |  |  |  |
|  | Inverse-variance weighted (fixed) | 4 | 0.81 | 0.64-1.03 | 0.088 | 0.628 |
|  | MR-Egger | 4 | / | / | 0.505* | / |
|  | Weighted median | 4 | 0.76 | 0.57-1.02 | 0.064 | / |
|  | Maximum-likelihood method | 4 | 0.81 | 0.64-1.03 | 0.089 | / |
|  | MR-PRESSO test | 4 | 0.81 | 0.68-0.97 | 0.111 | 0.66 |
| *Hungatella* |  |  |  |  |  |  |
|  | Inverse-variance weighted (fixed) | 4 | 1.07 | 0.86-1.34 | 0.531 | 0.357 |
|  | MR-Egger | 4 | / | / | 0.152* | / |
|  | Weighted median | 4 | 1.16 | 0.87-1.55 | 0.309 | / |
|  | Maximum-likelihood method | 4 | 1.08 | 0.85-1.36 | 0.538 | / |
|  | MR-PRESSO test | 4 | 1.07 | 0.85-1.35 | 0.589 | 0.324 |
| *Intestinibacter* |  |  |  |  |  |  |
|  | Inverse-variance weighted (fixed) | 4 | 1 | 0.88-1.14 | 0.995 | 0.762 |
|  | MR-Egger | 4 | / | / | 0.533* | / |
|  | Weighted median | 4 | 1 | 0.85-1.17 | 0.961 | / |
|  | Maximum-likelihood method | 4 | 1 | 0.87-1.14 | 0.995 | / |
|  | MR-PRESSO test | 4 | 1 | 0.92-1.09 | 0.992 | 0.777 |
| *Intestinimonas* |  |  |  |  |  |  |
|  | Inverse-variance weighted (fixed) | 4 | 0.9 | 0.79-1.02 | 0.108 | 0.33 |
|  | MR-Egger | 4 | / | / | 0.170* | / |
|  | Weighted median | 4 | 0.85 | 0.71-1.01 | 0.062 | / |
|  | Maximum-likelihood method | 4 | 0.89 | 0.77-1.03 | 0.128 | / |
|  | MR-PRESSO test | 4 | 0.9 | 0.78-1.03 | 0.23 | 0.262 |
| *Lachnoclostridium* |  |  |  |  |  |  |
|  | Inverse-variance weighted (fixed) | 4 | 0.95 | 0.85-1.05 | 0.31 | 0.806 |
|  | MR-Egger | 4 | / | / | 0.533* | / |
|  | Weighted median | 4 | 0.95 | 0.83-1.08 | 0.422 | / |
|  | Maximum-likelihood method | 4 | 0.95 | 0.85-1.05 | 0.311 | / |
|  | MR-PRESSO test | 4 | 0.95 | 0.89-1.01 | 0.174 | 0.818 |
| *Lachnospira* |  |  |  |  |  |  |
|  | Inverse-variance weighted (fixed) | 4 | 0.93 | 0.83-1.04 | 0.194 | 0.704 |
|  | MR-Egger | 4 | / | / | 0.505* | / |
|  | Weighted median | 4 | 0.92 | 0.8-1.05 | 0.22 | / |
|  | Maximum-likelihood method | 4 | 0.93 | 0.83-1.04 | 0.196 | / |
|  | MR-PRESSO test | 4 | 0.93 | 0.86-1.00 | 0.154 | 0.746 |
| *LachnospiraceaeFCS020group* |  |  |  |  |  |  |
|  | Inverse-variance weighted (fixed) | 4 | 1.05 | 0.93-1.19 | 0.46 | 0.319 |
|  | MR-Egger | 4 | / | / | 0.766* | / |
|  | Weighted median | 4 | 1.02 | 0.87-1.19 | 0.842 | / |
|  | Maximum-likelihood method | 4 | 1.05 | 0.91-1.21 | 0.489 | / |
|  | MR-PRESSO test | 4 | 1.05 | 0.92-1.20 | 0.543 | 0.378 |
| *LachnospiraceaeNC2004group* |  |  |  |  |  |  |
|  | Inverse-variance weighted (fixed) | 4 | 1.06 | 0.88-1.27 | 0.533 | 0.744 |
|  | MR-Egger | 4 | / | / | 0.897* | / |
|  | Weighted median | 4 | 1.05 | 0.85-1.31 | 0.644 | / |
|  | Maximum-likelihood method | 4 | 1.06 | 0.88-1.27 | 0.532 | / |
|  | MR-PRESSO test | 4 | 1.06 | 0.94-1.19 | 0.403 | 0.785 |
| *LachnospiraceaeND3007group* |  |  |  |  |  |  |
|  | Inverse-variance weighted (fixed) | 4 | 1 | 0.89-1.13 | 0.943 | 0.061 |
|  | MR-Egger | 4 | / | / | 0.052* | / |
|  | Weighted median | 4 | 0.95 | 0.81-1.12 | 0.567 | / |
|  | Maximum-likelihood method | 4 | 1 | 0.83-1.22 | 0.962 | / |
|  | MR-PRESSO test | 4 | 1 | 0.84-1.21 | 0.966 | 0.069 |
| *LachnospiraceaeNK4A136group* |  |  |  |  |  |  |
|  | Inverse-variance weighted (fixed) | 4 | 1.01 | 0.90-1.12 | 0.924 | 0.408 |
|  | MR-Egger | 4 | / | / | 0.294* | / |
|  | Weighted median | 4 | 0.99 | 0.86-1.14 | 0.914 | / |
|  | Maximum-likelihood method | 4 | 1.01 | 0.90-1.13 | 0.923 | / |
|  | MR-PRESSO test | 4 | 1.01 | 0.90-1.12 | 0.929 | 0.348 |
| *LachnospiraceaeUCG001* |  |  |  |  |  |  |
|  | Inverse-variance weighted (fixed) | 4 | 0.99 | 0.85-1.14 | 0.844 | 0.455 |
|  | MR-Egger | 4 | / | / | 0.369* | / |
|  | Weighted median | 4 | 1 | 0.84-1.20 | 0.972 | / |
|  | Maximum-likelihood method | 4 | 0.99 | 0.85-1.14 | 0.842 | / |
|  | MR-PRESSO test | 4 | 0.99 | 0.86-1.13 | 0.847 | 0.503 |
| *LachnospiraceaeUCG004* |  |  |  |  |  |  |
|  | Inverse-variance weighted (fixed) | 4 | 1.04 | 0.93-1.18 | 0.483 | 0.874 |
|  | MR-Egger | 4 | / | / | 0.668* | / |
|  | Weighted median | 4 | 1.04 | 0.90-1.20 | 0.593 | / |
|  | Maximum-likelihood method | 4 | 1.04 | 0.93-1.18 | 0.483 | / |
|  | MR-PRESSO test | 4 | 1.04 | 0.99-1.11 | 0.241 | 0.894 |
| *LachnospiraceaeUCG008* |  |  |  |  |  |  |
|  | Inverse-variance weighted (fixed) | 4 | 0.85 | 0.72-1.02 | 0.079 | 0.331 |
|  | MR-Egger | 4 | / | / | 0.522* | / |
|  | Weighted median | 4 | 0.81 | 0.65-1.01 | 0.06 | / |
|  | Maximum-likelihood method | 4 | 0.85 | 0.70-1.03 | 0.094 | / |
|  | MR-PRESSO test | 4 | 0.85 | 0.71-1.03 | 0.198 | 0.395 |
| *LachnospiraceaeUCG010* |  |  |  |  |  |  |
|  | Inverse-variance weighted (fixed) | 4 | 0.95 | 0.83-1.07 | 0.389 | 0.689 |
|  | MR-Egger | 4 | / | / | 0.468* | / |
|  | Weighted median | 4 | 0.95 | 0.81-1.10 | 0.475 | / |
|  | Maximum-likelihood method | 4 | 0.95 | 0.83-1.07 | 0.387 | / |
|  | MR-PRESSO test | 4 | 0.95 | 0.87-1.03 | 0.306 | 0.706 |
| *Lactobacillus* |  |  |  |  |  |  |
|  | Inverse-variance weighted (fixed) | 4 | 1.02 | 0.86-1.21 | 0.826 | 0.006 |
|  | MR-Egger | 4 | / | / | 0.001* | / |
|  | Weighted median | 4 | 1.16 | 0.88-1.53 | 0.285 | / |
|  | Maximum-likelihood method | 4 | 1.02 | 0.69-1.51 | 0.907 | / |
|  | MR-PRESSO test | 3 | 1.02 | 0.71-1.46 | 0.921 | 0.004 |
| *Lactococcus* |  |  |  |  |  |  |
|  | Inverse-variance weighted (fixed) | 4 | 0.86 | 0.68-1.08 | 0.191 | 0.755 |
|  | MR-Egger | 4 | / | / | 0.580* | / |
|  | Weighted median | 4 | 0.83 | 0.63-1.10 | 0.2 | / |
|  | Maximum-likelihood method | 4 | 0.86 | 0.68-1.08 | 0.193 | / |
|  | MR-PRESSO test | 4 | 0.86 | 0.74-0.99 | 0.13 | 0.777 |
| *Marvinbryantia* |  |  |  |  |  |  |
|  | Inverse-variance weighted (fixed) | 4 | 1 | 0.87-1.14 | 0.948 | 0.598 |
|  | MR-Egger | 4 | / | / | 0.956* | / |
|  | Weighted median | 4 | 0.96 | 0.81-1.12 | 0.585 | / |
|  | Maximum-likelihood method | 4 | 1 | 0.87-1.14 | 0.947 | / |
|  | MR-PRESSO test | 4 | 1 | 0.89-1.11 | 0.939 | 0.646 |
| *Methanobrevibacter* |  |  |  |  |  |  |
|  | Inverse-variance weighted (fixed) | 3 | 0.75 | 0.55-1.02 | 0.065 | 0.115 |
|  | MR-Egger | 3 | / | / | 0.785* | / |
|  | Weighted median | 3 | 0.78 | 0.51-1.19 | 0.254 | / |
|  | Maximum-likelihood method | 3 | 0.74 | 0.53-1.02 | 0.064 | / |
| *Odoribacter* |  |  |  |  |  |  |
|  | Inverse-variance weighted (fixed) | 4 | 0.9 | 0.80-1.02 | 0.094 | 0.798 |
|  | MR-Egger | 4 | / | / | 0.918* | / |
|  | Weighted median | 4 | 0.94 | 0.81-1.08 | 0.378 | / |
|  | Maximum-likelihood method | 4 | 0.9 | 0.80-1.02 | 0.098 | / |
|  | MR-PRESSO test | 4 | 0.9 | 0.84-0.97 | 0.063 | 0.791 |
| *Olsenella* |  |  |  |  |  |  |
|  | Inverse-variance weighted (fixed) | 4 | 0.98 | 0.77-1.25 | 0.877 | 0.553 |
|  | MR-Egger | 4 | / | / | 0.530* | / |
|  | Weighted median | 4 | 0.98 | 0.73-1.33 | 0.918 | / |
|  | Maximum-likelihood method | 4 | 0.98 | 0.77-1.25 | 0.876 | / |
|  | MR-PRESSO test | 4 | 0.98 | 0.80-1.20 | 0.865 | 0.603 |
| *Oscillibacter* |  |  |  |  |  |  |
|  | Inverse-variance weighted (fixed) | 4 | 1.02 | 0.87-1.19 | 0.816 | 0.579 |
|  | MR-Egger | 4 | / | / | 0.224* | / |
|  | Weighted median | 4 | 0.96 | 0.79-1.16 | 0.669 | / |
|  | Maximum-likelihood method | 4 | 1.02 | 0.87-1.19 | 0.815 | / |
|  | MR-PRESSO test | 4 | 1.02 | 0.90-1.16 | 0.793 | 0.576 |
| *Oscillospira* |  |  |  |  |  |  |
|  | Inverse-variance weighted (fixed) | 4 | 1.05 | 0.91-1.22 | 0.472 | 0.343 |
|  | MR-Egger | 4 | / | / | 0.410* | / |
|  | Weighted median | 4 | 1.01 | 0.84-1.21 | 0.947 | / |
|  | Maximum-likelihood method | 4 | 1.06 | 0.90-1.23 | 0.489 | / |
|  | MR-PRESSO test | 4 | 1.05 | 0.91-1.23 | 0.544 | 0.376 |
| *Oxalobacter* |  |  |  |  |  |  |
|  | Inverse-variance weighted (fixed) | 4 | 0.69 | 0.55-0.85 | 0.001 | 0.79 |
|  | MR-Egger | 4 | / | / | 0.999* | / |
|  | Weighted median | 4 | 0.69 | 0.53-0.90 | 0.007 | / |
|  | Maximum-likelihood method | 4 | 0.68 | 0.54-0.86 | 0.001 | / |
|  | MR-PRESSO test | 4 | 0.69 | 0.60-0.78 | 0.01 | 0.851 |
| *Parabacteroides* |  |  |  |  |  |  |
|  | Inverse-variance weighted (fixed) | 4 | 0.9 | 0.80-1.00 | 0.052 | 0.598 |
|  | MR-Egger | 4 | / | / | 0.945* | / |
|  | Weighted median | 4 | 0.88 | 0.77-1.01 | 0.065 | / |
|  | Maximum-likelihood method | 4 | 0.89 | 0.80-1.00 | 0.055 | / |
|  | MR-PRESSO test | 4 | 0.9 | 0.82-0.98 | 0.091 | 0.655 |
| *Paraprevotella* |  |  |  |  |  |  |
|  | Inverse-variance weighted (fixed) | 4 | 1.07 | 0.90-1.27 | 0.428 | 0.02 |
|  | MR-Egger | 4 | / | / | 0.258* | / |
|  | Weighted median | 4 | 1.01 | 0.77-1.31 | 0.955 | / |
|  | Maximum-likelihood method | 4 | 1.08 | 0.78-1.49 | 0.64 | / |
|  | MR-PRESSO test | 3 | 1.07 | 0.79-1.46 | 0.691 | 0.015 |
| *Parasutterella* |  |  |  |  |  |  |
|  | Inverse-variance weighted (fixed) | 4 | 0.93 | 0.81-1.07 | 0.294 | 0.505 |
|  | MR-Egger | 4 | / | / | 0.906* | / |
|  | Weighted median | 4 | 0.95 | 0.81-1.12 | 0.551 | / |
|  | Maximum-likelihood method | 4 | 0.93 | 0.81-1.07 | 0.291 | / |
|  | MR-PRESSO test | 4 | 0.93 | 0.82-1.05 | 0.32 | 0.552 |
| *Peptococcus* |  |  |  |  |  |  |
|  | Inverse-variance weighted (fixed) | 4 | 1.13 | 0.93-1.38 | 0.218 | 0.026 |
|  | MR-Egger | 4 | / | / | 0.865* | / |
|  | Weighted median | 4 | 1.06 | 0.82-1.37 | 0.64 | / |
|  | Maximum-likelihood method | 4 | 1.14 | 0.80-1.64 | 0.466 | / |
|  | MR-PRESSO test | 3 | 1.13 | 0.80-1.6 | 0.533 | 0.041 |
| *Phascolarctobacterium* |  |  |  |  |  |  |
|  | Inverse-variance weighted (fixed) | 4 | 0.95 | 0.83-1.09 | 0.503 | 0.853 |
|  | MR-Egger | 4 | / | / | 0.752* | / |
|  | Weighted median | 4 | 0.97 | 0.82-1.14 | 0.678 | / |
|  | Maximum-likelihood method | 4 | 0.95 | 0.83-1.10 | 0.502 | / |
|  | MR-PRESSO test | 4 | 0.95 | 0.89-1.02 | 0.282 | 0.834 |
| *Prevotella7* |  |  |  |  |  |  |
|  | Inverse-variance weighted (fixed) | 4 | 1.08 | 0.85-1.39 | 0.522 | 0.597 |
|  | MR-Egger | 4 | / | / | 0.180* | / |
|  | Weighted median | 4 | 1.03 | 0.76-1.40 | 0.845 | / |
|  | Maximum-likelihood method | 4 | 1.09 | 0.85-1.39 | 0.518 | / |
|  | MR-PRESSO test | 4 | 1.08 | 0.89-1.32 | 0.478 | 0.545 |
| *Prevotella9* |  |  |  |  |  |  |
|  | Inverse-variance weighted (fixed) | 4 | 1 | 0.87-1.15 | 0.998 | 0.576 |
|  | MR-Egger | 4 | / | / | 0.928* | / |
|  | Weighted median | 4 | 1 | 0.84-1.18 | 0.964 | / |
|  | Maximum-likelihood method | 4 | 1 | 0.87-1.15 | 0.998 | / |
|  | MR-PRESSO test | 4 | 1 | 0.89-1.12 | 0.998 | 0.632 |
| *RikenellaceaeRC9gutgroup* |  |  |  |  |  |  |
|  | Inverse-variance weighted (fixed) | 3 | 1.1 | 0.80-1.52 | 0.541 | 0.373 |
|  | MR-Egger | 3 | / | / | 0.166* | / |
|  | Weighted median | 3 | 1.17 | 0.79-1.74 | 0.426 | / |
|  | Maximum-likelihood method | 3 | 1.11 | 0.80-1.53 | 0.536 | / |
| *Romboutsia* |  |  |  |  |  |  |
|  | Inverse-variance weighted (fixed) | 4 | 0.93 | 0.83-1.05 | 0.267 | 0.957 |
|  | MR-Egger | 4 | / | / | 0.811* | / |
|  | Weighted median | 4 | 0.93 | 0.80-1.07 | 0.315 | / |
|  | Maximum-likelihood method | 4 | 0.93 | 0.82-1.06 | 0.27 | / |
|  | MR-PRESSO test | 4 | 0.93 | 0.90-0.97 | 0.041 | 0.959 |
| *Roseburia* |  |  |  |  |  |  |
|  | Inverse-variance weighted (fixed) | 4 | 0.96 | 0.86-1.07 | 0.45 | 0.509 |
|  | MR-Egger | 4 | / | / | 0.345* | / |
|  | Weighted median | 4 | 0.97 | 0.85-1.11 | 0.635 | / |
|  | Maximum-likelihood method | 4 | 0.96 | 0.86-1.07 | 0.445 | / |
|  | MR-PRESSO test | 4 | 0.96 | 0.87-1.06 | 0.453 | 0.553 |
| *Ruminiclostridium5* |  |  |  |  |  |  |
|  | Inverse-variance weighted (fixed) | 4 | 0.99 | 0.89-1.11 | 0.87 | 0.428 |
|  | MR-Egger | 4 | / | / | 0.120* | / |
|  | Weighted median | 4 | 1.03 | 0.89-1.18 | 0.692 | / |
|  | Maximum-likelihood method | 4 | 0.99 | 0.89-1.11 | 0.867 | / |
|  | MR-PRESSO test | 4 | 0.99 | 0.89-1.10 | 0.875 | 0.335 |
| *Ruminiclostridium6* |  |  |  |  |  |  |
|  | Inverse-variance weighted (fixed) | 4 | 0.95 | 0.84-1.08 | 0.46 | 0.013 |
|  | MR-Egger | 4 | / | / | 0.081* | / |
|  | Weighted median | 4 | 1.01 | 0.85-1.20 | 0.925 | / |
|  | Maximum-likelihood method | 4 | 0.95 | 0.73-1.22 | 0.681 | / |
|  | MR-PRESSO test | 3 | 0.95 | 0.75-1.21 | 0.724 | 0.015 |
| *Ruminiclostridium9* |  |  |  |  |  |  |
|  | Inverse-variance weighted (fixed) | 4 | 0.94 | 0.84-1.05 | 0.297 | 0.773 |
|  | MR-Egger | 4 | / | / | 0.465* | / |
|  | Weighted median | 4 | 0.94 | 0.82-1.08 | 0.376 | / |
|  | Maximum-likelihood method | 4 | 0.94 | 0.84-1.05 | 0.297 | / |
|  | MR-PRESSO test | 4 | 0.94 | 0.88-1.01 | 0.186 | 0.799 |
| *RuminococcaceaeNK4A214group* |  |  |  |  |  |  |
|  | Inverse-variance weighted (fixed) | 4 | 1 | 0.89-1.13 | 0.942 | 0.911 |
|  | MR-Egger | 4 | / | / | 0.511* | / |
|  | Weighted median | 4 | 0.98 | 0.85-1.13 | 0.776 | / |
|  | Maximum-likelihood method | 4 | 1 | 0.89-1.13 | 0.941 | / |
|  | MR-PRESSO test | 4 | 1 | 0.96-1.06 | 0.874 | 0.884 |
| *RuminococcaceaeUCG002* |  |  |  |  |  |  |
|  | Inverse-variance weighted (fixed) | 4 | 0.98 | 0.88-1.10 | 0.744 | 0.523 |
|  | MR-Egger | 4 | / | / | 0.278* | / |
|  | Weighted median | 4 | 1.02 | 0.89-1.16 | 0.817 | / |
|  | Maximum-likelihood method | 4 | 0.98 | 0.88-1.10 | 0.742 | / |
|  | MR-PRESSO test | 4 | 0.98 | 0.89-1.08 | 0.731 | 0.527 |
| *RuminococcaceaeUCG003* |  |  |  |  |  |  |
|  | Inverse-variance weighted (fixed) | 4 | 0.95 | 0.84-1.07 | 0.428 | 0.798 |
|  | MR-Egger | 4 | / | / | 0.564* | / |
|  | Weighted median | 4 | 0.95 | 0.82-1.09 | 0.457 | / |
|  | Maximum-likelihood method | 4 | 0.95 | 0.84-1.08 | 0.428 | / |
|  | MR-PRESSO test | 4 | 0.95 | 0.89-1.02 | 0.267 | 0.766 |
| *RuminococcaceaeUCG004* |  |  |  |  |  |  |
|  | Inverse-variance weighted (fixed) | 4 | 0.9 | 0.77-1.04 | 0.152 | 0.842 |
|  | MR-Egger | 4 | / | / | 0.867* | / |
|  | Weighted median | 4 | 0.88 | 0.73-1.05 | 0.153 | / |
|  | Maximum-likelihood method | 4 | 0.9 | 0.77-1.04 | 0.155 | / |
|  | MR-PRESSO test | 4 | 0.9 | 0.83-0.97 | 0.073 | 0.853 |
| *RuminococcaceaeUCG005* |  |  |  |  |  |  |
|  | Inverse-variance weighted (fixed) | 4 | 1.06 | 0.94-1.19 | 0.335 | 0.103 |
|  | MR-Egger | 4 | / | / | 0.570* | / |
|  | Weighted median | 4 | 1.07 | 0.91-1.24 | 0.409 | / |
|  | Maximum-likelihood method | 4 | 1.06 | 0.90-1.26 | 0.49 | / |
|  | MR-PRESSO test | 4 | 1.06 | 0.90-1.25 | 0.55 | 0.12 |
| *RuminococcaceaeUCG009* |  |  |  |  |  |  |
|  | Inverse-variance weighted (fixed) | 4 | 0.93 | 0.79-1.10 | 0.406 | 0.068 |
|  | MR-Egger | 4 | / | / | 0.998* | / |
|  | Weighted median | 4 | 1.01 | 0.81-1.26 | 0.946 | / |
|  | Maximum-likelihood method | 4 | 0.93 | 0.70-1.22 | 0.577 | / |
|  | MR-PRESSO test | 4 | 0.93 | 0.72-1.21 | 0.627 | 0.086 |
| *RuminococcaceaeUCG010* |  |  |  |  |  |  |
|  | Inverse-variance weighted (fixed) | 4 | 1.01 | 0.89-1.15 | 0.885 | 0.275 |
|  | MR-Egger | 4 | / | / | 0.335* | / |
|  | Weighted median | 4 | 1.04 | 0.88-1.23 | 0.618 | / |
|  | Maximum-likelihood method | 4 | 1.01 | 0.87-1.17 | 0.897 | / |
|  | MR-PRESSO test | 4 | 1.01 | 0.87-1.17 | 0.907 | 0.298 |
| *RuminococcaceaeUCG011* |  |  |  |  |  |  |
|  | Inverse-variance weighted (fixed) | 4 | 1 | 0.78-1.27 | 0.977 | 0.444 |
|  | MR-Egger | 4 | / | / | 0.963* | / |
|  | Weighted median | 4 | 1.11 | 0.82-1.50 | 0.499 | / |
|  | Maximum-likelihood method | 4 | 1 | 0.78-1.28 | 0.977 | / |
|  | MR-PRESSO test | 4 | 1 | 0.79-1.26 | 0.978 | 0.468 |
| *RuminococcaceaeUCG013* |  |  |  |  |  |  |
|  | Inverse-variance weighted (fixed) | 4 | 1.14 | 1.02-1.27 | 0.024 | 0.164 |
|  | MR-Egger | 4 | / | / | 0.061* | / |
|  | Weighted median | 4 | 1.06 | 0.91-1.24 | 0.425 | / |
|  | Maximum-likelihood method | 4 | 1.15 | 0.98-1.34 | 0.079 | / |
|  | MR-PRESSO test | 4 | 1.14 | 0.98-1.32 | 0.183 | 0.171 |
| *RuminococcaceaeUCG014* |  |  |  |  |  |  |
|  | Inverse-variance weighted (fixed) | 4 | 0.97 | 0.86-1.10 | 0.649 | 0.296 |
|  | MR-Egger | 4 | / | / | 0.980* | / |
|  | Weighted median | 4 | 0.94 | 0.81-1.09 | 0.419 | / |
|  | Maximum-likelihood method | 4 | 0.97 | 0.85-1.11 | 0.676 | / |
|  | MR-PRESSO test | 4 | 0.97 | 0.85-1.11 | 0.71 | 0.318 |
| *Ruminococcus1* |  |  |  |  |  |  |
|  | Inverse-variance weighted (fixed) | 4 | 1.12 | 1.00-1.26 | 0.046 | 0.268 |
|  | MR-Egger | 4 | / | / | 0.323* | / |
|  | Weighted median | 4 | 1.18 | 1.02-1.36 | 0.026 | / |
|  | Maximum-likelihood method | 4 | 1.13 | 0.99-1.28 | 0.075 | / |
|  | MR-PRESSO test | 4 | 1.12 | 0.99-1.28 | 0.18 | 0.256 |
| *Ruminococcus2* |  |  |  |  |  |  |
|  | Inverse-variance weighted (fixed) | 4 | 0.96 | 0.85-1.08 | 0.454 | 0.153 |
|  | MR-Egger | 4 | / | / | 0.192* | / |
|  | Weighted median | 4 | 1 | 0.85-1.18 | 0.993 | / |
|  | Maximum-likelihood method | 4 | 0.95 | 0.81-1.12 | 0.557 | / |
|  | MR-PRESSO test | 4 | 0.96 | 0.82-1.12 | 0.611 | 0.113 |
| *Ruminococcusgauvreauiigroup* |  |  |  |  |  |  |
|  | Inverse-variance weighted (fixed) | 4 | 1.03 | 0.901.16 | 0.688 | 0.086 |
|  | MR-Egger | 4 | / | / | 0.548* | / |
|  | Weighted median | 4 | 1.04 | 0.88-1.23 | 0.619 | / |
|  | Maximum-likelihood method | 4 | 1.03 | 0.85-1.25 | 0.78 | / |
|  | MR-PRESSO test | 4 | 1.03 | 0.85-1.24 | 0.804 | 0.12 |
| *Ruminococcusgnavusgroup* |  |  |  |  |  |  |
|  | Inverse-variance weighted (fixed) | 4 | 0.93 | 0.77-1.11 | 0.404 | 0.716 |
|  | MR-Egger | 4 | / | / | 0.667* | / |
|  | Weighted median | 4 | 0.95 | 0.77-1.19 | 0.665 | / |
|  | Maximum-likelihood method | 4 | 0.93 | 0.77-1.11 | 0.403 | / |
|  | MR-PRESSO test | 4 | 0.93 | 0.82-1.05 | 0.303 | 0.764 |
| *Ruminococcustorquesgroup* |  |  |  |  |  |  |
|  | Inverse-variance weighted (fixed) | 4 | 0.94 | 0.84-1.05 | 0.278 | 0.935 |
|  | MR-Egger | 4 | / | / | 0.873* | / |
|  | Weighted median | 4 | 0.95 | 0.83-1.08 | 0.39 | / |
|  | Maximum-likelihood method | 4 | 0.94 | 0.84-1.05 | 0.281 | / |
|  | MR-PRESSO test | 4 | 0.94 | 0.90-0.98 | 0.063 | 0.937 |
| *Sellimonas* |  |  |  |  |  |  |
|  | Inverse-variance weighted (fixed) | 3 | 0.91 | 0.66-1.26 | 0.562 | 0.563 |
|  | MR-Egger | 3 | / | / | 0.450* | / |
|  | Weighted median | 3 | 0.95 | 0.64-1.42 | 0.817 | / |
|  | Maximum-likelihood method | 3 | 0.91 | 0.65-1.26 | 0.56 | / |
| *Senegalimassilia* |  |  |  |  |  |  |
|  | Inverse-variance weighted (fixed) | 4 | 0.98 | 0.82-1.17 | 0.817 | 0.296 |
|  | MR-Egger | 4 | / | / | 0.484* | / |
|  | Weighted median | 4 | 0.97 | 0.78-1.21 | 0.794 | / |
|  | Maximum-likelihood method | 4 | 0.98 | 0.80-1.20 | 0.832 | / |
|  | MR-PRESSO test | 4 | 0.98 | 0.80-1.19 | 0.848 | 0.309 |
| *Slackia* |  |  |  |  |  |  |
|  | Inverse-variance weighted (fixed) | 4 | 0.87 | 0.72-1.05 | 0.145 | 0.034 |
|  | MR-Egger | 4 | / | / | 0.305* | / |
|  | Weighted median | 4 | 0.83 | 0.65-1.07 | 0.154 | / |
|  | Maximum-likelihood method | 4 | 0.86 | 0.61-1.20 | 0.372 | / |
|  | MR-PRESSO test | 4 | 0.87 | 0.63-1.20 | 0.454 | 0.055 |
| *Streptococcus* |  |  |  |  |  |  |
|  | Inverse-variance weighted (fixed) | 4 | 1.1 | 0.98-1.23 | 0.098 | 0.316 |
|  | MR-Egger | 4 | / | / | 0.121* | / |
|  | Weighted median | 4 | 1.07 | 0.92-1.23 | 0.397 | / |
|  | Maximum-likelihood method | 4 | 1.11 | 0.97-1.25 | 0.122 | / |
|  | MR-PRESSO test | 4 | 1.1 | 0.97-1.25 | 0.225 | 0.332 |
| *Subdoligranulum* |  |  |  |  |  |  |
|  | Inverse-variance weighted (fixed) | 4 | 0.98 | 0.88-1.09 | 0.673 | 0.982 |
|  | MR-Egger | 4 | / | / | 0.862* | / |
|  | Weighted median | 4 | 0.97 | 0.86-1.11 | 0.676 | / |
|  | Maximum-likelihood method | 4 | 0.98 | 0.88-1.09 | 0.673 | / |
|  | MR-PRESSO test | 4 | 0.98 | 0.95-1.00 | 0.176 | 0.981 |
| *Sutterella* |  |  |  |  |  |  |
|  | Inverse-variance weighted (fixed) | 4 | 0.99 | 0.87-1.12 | 0.833 | 0.726 |
|  | MR-Egger | 4 | / | / | 0.788* | / |
|  | Weighted median | 4 | 0.98 | 0.84-1.14 | 0.782 | / |
|  | Maximum-likelihood method | 4 | 0.99 | 0.87-1.12 | 0.832 | / |
|  | MR-PRESSO test | 4 | 0.99 | 0.91-1.07 | 0.772 | 0.75 |
| *Terrisporobacter* |  |  |  |  |  |  |
|  | Inverse-variance weighted (fixed) | 4 | 1.02 | 0.86-1.23 | 0.791 | 0.492 |
|  | MR-Egger | 4 | / | / | 0.348* | / |
|  | Weighted median | 4 | 1.06 | 0.85-1.33 | 0.609 | / |
|  | Maximum-likelihood method | 4 | 1.03 | 0.85-1.23 | 0.788 | / |
|  | MR-PRESSO test | 4 | 1.02 | 0.87-1.20 | 0.786 | 0.528 |
| *Turicibacter* |  |  |  |  |  |  |
|  | Inverse-variance weighted (fixed) | 4 | 1.01 | 0.87-1.18 | 0.892 | 0.482 |
|  | MR-Egger | 4 | / | / | 0.880* | / |
|  | Weighted median | 4 | 0.99 | 0.82-1.20 | 0.956 | / |
|  | Maximum-likelihood method | 4 | 1.01 | 0.86-1.18 | 0.891 | / |
|  | MR-PRESSO test | 4 | 1.01 | 0.88-1.16 | 0.891 | 0.54 |
| *Tyzzerella3* |  |  |  |  |  |  |
|  | Inverse-variance weighted (fixed) | 4 | 1.08 | 0.89-1.31 | 0.454 | 0.799 |
|  | MR-Egger | 4 | / | / | 0.477* | / |
|  | Weighted median | 4 | 1.04 | 0.82-1.33 | 0.741 | / |
|  | Maximum-likelihood method | 4 | 1.08 | 0.88-1.32 | 0.453 | / |
|  | MR-PRESSO test | 4 | 1.08 | 0.96-1.21 | 0.287 | 0.824 |
| *Veillonella* |  |  |  |  |  |  |
|  | Inverse-variance weighted (fixed) | 4 | 1.05 | 0.90-1.22 | 0.545 | 0.877 |
|  | MR-Egger | 4 | / | / | 0.537* | / |
|  | Weighted median | 4 | 1.02 | 0.85-1.21 | 0.864 | / |
|  | Maximum-likelihood method | 4 | 1.05 | 0.90-1.22 | 0.544 | / |
|  | MR-PRESSO test | 4 | 1.05 | 0.98-1.12 | 0.295 | 0.838 |
| *Victivallis* |  |  |  |  |  |  |
|  | Inverse-variance weighted (fixed) | 4 | 1.02 | 0.79-1.31 | 0.887 | 0.006 |
|  | MR-Egger | 4 | / | / | 0.147* | / |
|  | Weighted median | 4 | 1.14 | 0.77-1.69 | 0.502 | / |
|  | Maximum-likelihood method | 4 | 1.02 | 0.59-1.76 | 0.939 | / |
|  | MR-PRESSO test | 3 | 1.02 | 0.62-1.68 | 0.948 | 0.007 |
| **Order** |  |  |  |  |  |  |
| *Actinomycetales* |  |  |  |  |  |  |
|  | Inverse-variance weighted (fixed) | 4 | 1.02 | 0.86-1.21 | 0.799 | 0.612 |
|  | MR-Egger | 4 | / | / | 0.804* | / |
|  | Weighted median | 4 | 1.05 | 0.86-1.29 | 0.615 | / |
|  | Maximum-likelihood method | 4 | 1.02 | 0.86-1.21 | 0.797 | / |
|  | MR-PRESSO test | 4 | 1.02 | 0.90-1.17 | 0.764 | 0.670 |
| *Bacillales* |  |  |  |  |  |  |
|  | Inverse-variance weighted (fixed) | 3 | 0.91 | 0.66-1.25 | 0.560 | 0.521 |
|  | MR-Egger | 3 | / | / | 0.476* | / |
|  | Weighted median | 3 | 1.02 | 0.69-1.52 | 0.915 | / |
|  | Maximum-likelihood method | 3 | 0.91 | 0.65-1.26 | 0.557 | / |
| *Bacteroidales* |  |  |  |  |  |  |
|  | Inverse-variance weighted (fixed) | 4 | 0.99 | 0.89-1.11 | 0.898 | 0.247 |
|  | MR-Egger | 4 | / | / | 0.781* | / |
|  | Weighted median | 4 | 1.02 | 0.90-1.17 | 0.727 | / |
|  | Maximum-likelihood method | 4 | 0.99 | 0.87-1.13 | 0.912 | / |
|  | MR-PRESSO test | 4 | 0.99 | 0.88-1.13 | 0.920 | 0.318 |
| *Bifidobacteriales* |  |  |  |  |  |  |
|  | Inverse-variance weighted (fixed) | 4 | 1.02 | 0.90-1.15 | 0.783 | 0.387 |
|  | MR-Egger | 4 | / | / | 0.302* | / |
|  | Weighted median | 4 | 1.03 | 0.89-1.2 | 0.681 | / |
|  | Maximum-likelihood method | 4 | 1.02 | 0.90-1.15 | 0.781 | / |
|  | MR-PRESSO test | 4 | 1.02 | 0.90-1.15 | 0.802 | 0.335 |
| *Burkholderiales* |  |  |  |  |  |  |
|  | Inverse-variance weighted (fixed) | 4 | 0.93 | 0.83-1.04 | 0.204 | 0.945 |
|  | MR-Egger | 4 | / | / | 0.649* | / |
|  | Weighted median | 4 | 0.93 | 0.82-1.06 | 0.306 | / |
|  | Maximum-likelihood method | 4 | 0.93 | 0.83-1.04 | 0.208 | / |
|  | MR-PRESSO test | 4 | 0.93 | 0.89-0.97 | 0.037 | 0.939 |
| *Clostridiales* |  |  |  |  |  |  |
|  | Inverse-variance weighted (fixed) | 4 | 1.01 | 0.90-1.12 | 0.920 | 0.136 |
|  | MR-Egger | 4 | / | / | 0.712* | / |
|  | Weighted median | 4 | 1.00 | 0.87-1.15 | 0.976 | / |
|  | Maximum-likelihood method | 4 | 1.01 | 0.87-1.17 | 0.940 | / |
|  | MR-PRESSO test | 4 | 1.01 | 0.87-1.16 | 0.946 | 0.166 |
| *Coriobacteriales* |  |  |  |  |  |  |
|  | Inverse-variance weighted (fixed) | 4 | 0.99 | 0.88-1.1 | 0.796 | 0.373 |
|  | MR-Egger | 4 | / | / | 0.922* | / |
|  | Weighted median | 4 | 0.96 | 0.83-1.1 | 0.524 | / |
|  | Maximum-likelihood method | 4 | 0.99 | 0.88-1.1 | 0.797 | / |
|  | MR-PRESSO test | 4 | 0.99 | 0.88-1.1 | 0.816 | 0.424 |
| *Desulfovibrionales* |  |  |  |  |  |  |
|  | Inverse-variance weighted (fixed) | 4 | 1.01 | 0.90-1.13 | 0.897 | 0.755 |
|  | MR-Egger | 4 | / | / | 0.950* | / |
|  | Weighted median | 4 | 1.02 | 0.89-1.17 | 0.762 | / |
|  | Maximum-likelihood method | 4 | 1.01 | 0.90-1.13 | 0.896 | / |
|  | MR-PRESSO test | 4 | 1.01 | 0.94-1.09 | 0.850 | 0.766 |
| *Enterobacteriales* |  |  |  |  |  |  |
|  | Inverse-variance weighted (fixed) | 4 | 0.92 | 0.81-1.04 | 0.187 | 0.638 |
|  | MR-Egger | 4 | / | / | 0.789* | / |
|  | Weighted median | 4 | 0.91 | 0.78-1.05 | 0.199 | / |
|  | Maximum-likelihood method | 4 | 0.92 | 0.81-1.04 | 0.187 | / |
|  | MR-PRESSO test | 4 | 0.92 | 0.84-1.01 | 0.177 | 0.685 |
| *Erysipelotrichales* |  |  |  |  |  |  |
|  | Inverse-variance weighted (fixed) | 4 | 0.96 | 0.86-1.06 | 0.408 | 0.428 |
|  | MR-Egger | 4 | / | / | 0.141* | / |
|  | Weighted median | 4 | 0.91 | 0.79-1.05 | 0.190 | / |
|  | Maximum-likelihood method | 4 | 0.95 | 0.86-1.06 | 0.402 | / |
|  | MR-PRESSO test | 4 | 0.96 | 0.86-1.06 | 0.453 | 0.430 |
| *Gastranaerophilales* |  |  |  |  |  |  |
|  | Inverse-variance weighted (fixed) | 4 | 0.93 | 0.77-1.12 | 0.429 | 0.549 |
|  | MR-Egger | 4 | / | / | 0.282* | / |
|  | Weighted median | 4 | 0.87 | 0.69-1.11 | 0.264 | / |
|  | Maximum-likelihood method | 4 | 0.92 | 0.76-1.12 | 0.426 | / |
|  | MR-PRESSO test | 4 | 0.93 | 0.79-1.09 | 0.416 | 0.489 |
| *Lactobacillales* |  |  |  |  |  |  |
|  | Inverse-variance weighted (fixed) | 4 | 1.10 | 0.99-1.23 | 0.080 | 0.083 |
|  | MR-Egger | 4 | / | / | 0.309* | / |
|  | Weighted median | 4 | 1.11 | 0.96-1.29 | 0.162 | / |
|  | Maximum-likelihood method | 4 | 1.11 | 0.94-1.32 | 0.228 | / |
|  | MR-PRESSO test | 4 | 1.10 | 0.94-1.30 | 0.325 | 0.119 |
| *Methanobacteriales* |  |  |  |  |  |  |
|  | Inverse-variance weighted (fixed) | 3 | 0.82 | 0.60-1.10 | 0.185 | 0.127 |
|  | MR-Egger | 3 | / | / | 0.764* | / |
|  | Weighted median | 3 | 0.86 | 0.57-1.30 | 0.471 | / |
|  | Maximum-likelihood method | 3 | 0.81 | 0.59-1.10 | 0.178 | / |
| *MollicutesRF9* |  |  |  |  |  |  |
|  | Inverse-variance weighted (fixed) | 4 | 1.10 | 0.95-1.27 | 0.203 | 0.922 |
|  | MR-Egger | 4 | / | / | 0.789* | / |
|  | Weighted median | 4 | 1.09 | 0.92-1.29 | 0.310 | / |
|  | Maximum-likelihood method | 4 | 1.10 | 0.95-1.27 | 0.206 | / |
|  | MR-PRESSO test | 4 | 1.10 | 1.04-1.16 | 0.051 | 0.936 |
| *NB1n* |  |  |  |  |  |  |
|  | Inverse-variance weighted (fixed) | 4 | 0.84 | 0.68-1.03 | 0.087 | 0.091 |
|  | MR-Egger | 4 | / | / | 0.022* | / |
|  | Weighted median | 4 | 0.95 | 0.73-1.24 | 0.708 | / |
|  | Maximum-likelihood method | 4 | 0.83 | 0.60-1.13 | 0.230 | / |
|  | MR-PRESSO test | 4 | 0.84 | 0.62-1.13 | 0.328 | 0.097 |
| *Pasteurellales* |  |  |  |  |  |  |
|  | Inverse-variance weighted (fixed) | 4 | 0.92 | 0.79-1.07 | 0.276 | 0.575 |
|  | MR-Egger | 4 | / | / | 0.173* | / |
|  | Weighted median | 4 | 0.92 | 0.77-1.11 | 0.408 | / |
|  | Maximum-likelihood method | 4 | 0.92 | 0.79-1.07 | 0.276 | / |
|  | MR-PRESSO test | 4 | 0.92 | 0.82-1.04 | 0.273 | 0.499 |
| *Rhodospirillales* |  |  |  |  |  |  |
|  | Inverse-variance weighted (fixed) | 4 | 1.10 | 0.94-1.28 | 0.250 | 0.711 |
|  | MR-Egger | 4 | / | / | 0.615* | / |
|  | Weighted median | 4 | 1.08 | 0.89-1.31 | 0.425 | / |
|  | Maximum-likelihood method | 4 | 1.10 | 0.94-1.29 | 0.250 | / |
|  | MR-PRESSO test | 4 | 1.10 | 0.99-1.22 | 0.188 | 0.748 |
| *Selenomonadales* |  |  |  |  |  |  |
|  | Inverse-variance weighted (fixed) | 4 | 0.99 | 0.89-1.11 | 0.894 | 0.899 |
|  | MR-Egger | 4 | / | / | 0.669* | / |
|  | Weighted median | 4 | 1.00 | 0.88-1.13 | 0.951 | / |
|  | Maximum-likelihood method | 4 | 0.99 | 0.89-1.11 | 0.894 | / |
|  | MR-PRESSO test | 4 | 0.99 | 0.95-1.04 | 0.784 | 0.874 |
| *Verrucomicrobiales* |  |  |  |  |  |  |
|  | Inverse-variance weighted (fixed) | 4 | 0.97 | 0.85-1.11 | 0.703 | 0.266 |
|  | MR-Egger | 4 | / | / | 0.549* | / |
|  | Weighted median | 4 | 0.97 | 0.82-1.15 | 0.763 | / |
|  | Maximum-likelihood method | 4 | 0.97 | 0.83-1.14 | 0.735 | / |
|  | MR-PRESSO test | 4 | 0.97 | 0.84-1.14 | 0.762 | 0.295 |
| *Victivallales* |  |  |  |  |  |  |
|  | Inverse-variance weighted (fixed) | 4 | 1.12 | 0.91-1.38 | 0.271 | 0.287 |
|  | MR-Egger | 4 | / | / | 0.647* | / |
|  | Weighted median | 4 | 1.07 | 0.82-1.39 | 0.640 | / |
|  | Maximum-likelihood method | 4 | 1.13 | 0.89-1.43 | 0.319 | / |
|  | MR-PRESSO test | 4 | 1.12 | 0.89-1.42 | 0.399 | 0.307 |
| **Phylum** |  |  |  |  |  |  |
| *Actinobacteria* |  |  |  |  |  |  |
|  | Inverse-variance weighted (fixed) | 4 | 1.00 | 0.90-1.12 | 0.951 | 0.497 |
|  | MR-Egger | 4 | / | / | 0.917* | / |
|  | Weighted median | 4 | 0.98 | 0.86-1.12 | 0.810 | / |
|  | Maximum-likelihood method | 4 | 1.00 | 0.90-1.12 | 0.951 | / |
|  | MR-PRESSO global test | 4 | 1.00 | 0.91-1.11 | 0.950 | 0.504 |
| *Bacteroidetes* |  |  |  |  |  |  |
|  | Inverse-variance weighted (fixed) | 4 | 1.00 | 0.90-1.11 | 0.948 | 0.227 |
|  | MR-Egger | 4 | / | / | 0.844* | / |
|  | Weighted median | 4 | 1.03 | 0.90-1.18 | 0.669 | / |
|  | Maximum-likelihood method | 4 | 1.00 | 0.87-1.14 | 0.956 | / |
|  | MR-PRESSO test | 4 | 1.00 | 0.88-1.13 | 0.961 | 0.301 |
| *Cyanobacteria* |  |  |  |  |  |  |
|  | Inverse-variance weighted (fixed) | 4 | 0.96 | 0.81-1.14 | 0.632 | 0.500 |
|  | MR-Egger | 4 | / | / | 0.147* | / |
|  | Weighted median | 4 | 1.00 | 0.81-1.24 | 0.994 | / |
|  | Maximum-likelihood method | 4 | 0.96 | 0.80-1.14 | 0.629 | / |
|  | MR-PRESSO test | 4 | 0.96 | 0.82-1.12 | 0.628 | 0.459 |
| *Euryarchaeota* |  |  |  |  |  |  |
|  | Inverse-variance weighted (fixed) | 4 | 0.85 | 0.67-1.08 | 0.189 | 0.368 |
|  | MR-Egger | 4 | / | / | 0.769* | / |
|  | Weighted median | 4 | 0.83 | 0.62-1.10 | 0.198 | / |
|  | Maximum-likelihood method | 4 | 0.85 | 0.66-1.09 | 0.194 | / |
|  | MR-PRESSO test | 4 | 0.85 | 0.67-1.09 | 0.290 | 0.422 |
| *Firmicutes* |  |  |  |  |  |  |
|  | Inverse-variance weighted (fixed) | 4 | 1.01 | 0.91-1.12 | 0.856 | 0.141 |
|  | MR-Egger | 4 | / | / | 0.895* | / |
|  | Weighted median | 4 | 1.02 | 0.89-1.17 | 0.757 | / |
|  | Maximum-likelihood method | 4 | 1.01 | 0.87-1.17 | 0.890 | / |
|  | MR-PRESSO test | 4 | 1.01 | 0.87-1.17 | 0.902 | 0.190 |
| *Lentisphaerae* |  |  |  |  |  |  |
|  | Inverse-variance weighted (fixed) | 4 | 1.12 | 0.91-1.38 | 0.269 | 0.233 |
|  | MR-Egger | 4 | / | / | 0.600* | / |
|  | Weighted median | 4 | 1.07 | 0.82-1.40 | 0.605 | / |
|  | Maximum-likelihood method | 4 | 1.13 | 0.88-1.46 | 0.346 | / |
|  | MR-PRESSO test | 4 | 1.12 | 0.88-1.44 | 0.423 | 0.244 |
| *Proteobacteria* |  |  |  |  |  |  |
|  | Inverse-variance weighted (fixed) | 4 | 0.98 | 0.88-1.09 | 0.669 | 0.034 |
|  | MR-Egger | 4 | / | / | 0.920* | / |
|  | Weighted median | 4 | 0.90 | 0.79-1.04 | 0.153 | / |
|  | Maximum-likelihood method | 4 | 0.97 | 0.81-1.18 | 0.792 | / |
|  | MR-PRESSO test | 3 | 0.98 | 0.81-1.17 | 0.818 | 0.040 |
| *Tenericutes* |  |  |  |  |  |  |
|  | Inverse-variance weighted (fixed) | 4 | 1.14 | 1.00-1.31 | 0.059 | 0.348 |
|  | MR-Egger | 4 | / | / | 0.112* | / |
|  | Weighted median | 4 | 1.22 | 1.02-1.46 | 0.028 | / |
|  | Maximum-likelihood method | 4 | 1.15 | 0.99-1.32 | 0.067 | / |
|  | MR-PRESSO test | 4 | 1.14 | 0.99-1.32 | 0.170 | 0.339 |
| *Verrucomicrobia* |  |  |  |  |  |  |
|  | Inverse-variance weighted (fixed) | 4 | 0.98 | 0.86-1.12 | 0.783 | 0.489 |
|  | MR-Egger | 4 | / | / | 0.499* | / |
|  | Weighted median | 4 | 0.98 | 0.83-1.15 | 0.793 | / |
|  | Maximum-likelihood method | 4 | 0.98 | 0.86-1.12 | 0.780 | / |
|  | MR-PRESSO test | 4 | 0.98 | 0.87-1.10 | 0.779 | 0.508 |

**Abbreviations:** *CI*, confidence interval; *MR*, Mendelian randomization; *MR-PRESSO test*, MR Pleiotropy RESidual Sum and Outlier test; *OR*, odds ratio; *SNP*, single nucleotide polymorphism.

**Note:** **p*-value of the intercept from MR-Egger regression analysis.

# Table S7. Summary of MR Studies related to periodontitis.

| **Title** | **Exposure** | **Association** | **OR** | **95% CI** | **PMID** |
| --- | --- | --- | --- | --- | --- |
| ***Diseases*** |  |  |  |  |  |
| Two-Sample Mendelian Randomization Analysis of Associations Between Periodontal Disease and Risk of Cancer | Cancer | ↑ | NA | NA | 34222791 |
| Relationship between periodontitis and psoriasis: A two-sample Mendelian randomization study | Psoriasis | Not found | 1.01 | 0.97-1.04 | 35362630 |
| Causal Association Between Periodontitis and Type 2 Diabetes: A Bidirectional Two-Sample Mendelian Randomization Analysis | Type 2 diabetes | Not found | 1.02 | 1.00-1.04 | 35082834 |
| Mendelian randomization supports the causal role of fasting glucose on periodontitis | Fasting glucose | ↑ | 1.12 | 1.05-1.20 | 35992145 |
| Association between inflammatory bowel disease and periodontitis: A bidirectional two‐sample Mendelian randomization study | Inflammatory bowel disease | ↑ | 1.06 | 1.02-1.11 | 36697037 |
| Association between periodontitis and breast cancer: two-sample Mendelian randomization study | Breast cancer | Not found | 0.95 | 0.89-1.02 | 36749410 |
| Periodontitis and stroke: A Mendelian randomization study | Stroke | Not found | 1.02 | 0.99-1.04 | 36621868 |
| Exploring the causal relationship between gastroesophageal reflux and oral lesions: A mendelian randomization study | Gastroesophageal reﬂux | ↑ | NA | NA | 36523763 |
| Gastroesophageal reflux disease and oral symptoms: A two-sample Mendelian randomization study | Gastroesophageal reﬂux | ↑ | 1.23 | 1.08-1.40 | 36685839 |
| Assessment of Bidirectional Relationships Between Polycystic Ovary Syndrome and Periodontitis: Insights From a Mendelian Randomization Analysis | Polycystic ovary syndrome | Not found | 0.97 | 0.88-1.06 | 33868379 |
| Causal association between asthma and periodontitis: A two-­sample Mendelian randomization analysis | Asthma | ↓ | 0.34 | 0.13-0.87 | 36959704 |
| Causal Inference Between Chronic Periodontitis and Chronic Kidney Disease: A Bidirectional Mendelian Randomization Analysis in a European Population | Chronic kidney disease | Not found | NA | NA | 34163528 |
| Causal Association between Periodontal Diseases and Cardiovascular Diseases | Cardiovascular Diseases | Not found | NA | NA | 35052354 |
| Is periodontitis a risk factor for ischaemic stroke, coronary artery disease and subclinical atherosclerosis? A Mendelian randomization study | Subclinical atherosclerosis | Not found | 0.99 | 0.97-1.02 | 3038664 |
| No Genetic Causal Association Between Periodontitis and Arthritis: A Bidirectional Two-Sample Mendelian Randomization Analysis | Rheumatoid arthritis | Not found | 1.01 | 0.97-1.06 | 35154127 |
|  | Osteoarthritis | Not found | 0.90 | 0.81-1.00 |  |
| ***Social behavior factors*** |  |  |  |  |  |
| Testing the association between tobacco smoking, alcohol consumption, and risk of periodontitis: A Mendelian randomization study | Cigarettes per day | ↑ | 1.56 | 1.18-2.07 | 34472130 |
|  | Smoking | ↑ | 1.26 | 1.04-1.53 |  |
|  | Alcohol consumption | ↑ | 1.41 | 1.04-1.90 |  |
| Association of short sleep with risk of periodontal disease: A meta‐analysis and Mendelian randomization study | Short sleep | Not found | 1.13 | 0.99-1.28 | 34109656 |
| No bidirectional relationship between depression and periodontitis: A genetic correlation and Mendelian randomization study | Depression | Not found | 1.05 | 0.95-1.15 | 35935963 |
| Appraising the causal role of smoking in multiple diseases: A systematic review and meta-analysis of Mendelian randomization studies | Smoking | ↑ | 1.47 | 1.35-1.61 | 35816897 |
| Cannabis use and the risk of periodontitis: A two‐sample Mendelian randomization study | Cannabis use | Not found | 1.05 | 0.93-1.19 | 35451161 |
| Understanding the consequences of educational inequalities on periodontitis: A Mendelian randomization study | Educational attainment | ↓ | 0.78 | 0.68-0.89 | 34866211 |
| ***Traits*** |  |  |  |  |  |
| A Mendelian randomization study on the effect of 25‐hydroxyvitamin D levels on periodontitis | 25-hydroxyvitamin D levels | Not found | 1.04 | 0.97-1.12 | 36274052 |
| Circulating vitamin C and D concentrations and risk of dental caries and periodontitis: A Mendelian randomization study | Circulating vitamin C | Not found | NA | NA | 35112385 |
|  | Circulating vitamin D | Not found | NA | NA |  |
| Reverse causal relationship between periodontitis and shortened telomere length: Bidirectional two-sample Mendelian random analysis | Shortened telomere length | ↑ | 1.06 | 1.02-1.10 | 36601105 |
| Mendelian randomization highlights the causal association of obesity with periodontal diseases | Body mass index | ↑ | 1.12 | 1.06-1.17 | 35569024 |
|  | Waist circumference | ↑ | 1.12 | 1.05-1.19 |  |
| Using genetics to test the causal relationship of total adiposity and periodontitis: Mendelian randomization analyses in the Gene-Lifestyle Interactions and Dental Endpoints Consortium | Obesity | Not found | 0.90 | 0.56-1.46 | 26050256 |
| Association between total body bone mineral density and periodontitis: A Mendelian randomization study | Bone mineral density | Not found | 1.00 | 0.92-1.08 | 36433673 |
| Inhibition of tumor necrosis factor receptor 1 and the risk of periodontitis | TNFR-1 | Not found | 1.57 | 0.38-6.46 | 36845132 |
| Assessment of bidirectional relationships between circulating cytokines and periodontitis: Insights from a mendelian randomization analysis | Circulating IL-9 | ↑ | 1.12 | 1.05-1.37 | 36793899 |
|  | Circulating IL-17 | ↓ | 0.85 | 0.74-0.98 |  |

**Abbreviation:** *CI*, Confidence interval; *IL*, Interleukin; *OR*, Odds ratio; *TNFR*, Tumor necrosis factor receptor.
