# Supplementary material for: Genetic evidence strengthens the bidirectional connection between gut microbiota and periodontitis: insights from a two-sample Mendelian randomization study
Source: J Transl Med. 2023 Sep 28;21:674. doi: 10.1186/s12967-023-04559-9 (PMC10537583; doi:10.1186/s12967-023-04559-9)
Supplement: Supplementary file 2 — Additional file 2: Fig. S1. Forest plot of the results after removing potential pleiotropic SNPs. Fig. S2. Results of leave-one-out sensitivity analysis. Fig. S3. Forest plot of the results in reverse MR for the causal effects of periodontitis on genetically predicted gut microbiota composition. [file 12967_2023_4559_MOESM2_ESM.docx]

Supporting information for

**Genetic evidence strengthens the bidirectional connection between gut microbiota and periodontitis: insights from a two-sample Mendelian randomization study**

*Xinjian Ye, Bin Liu, Yijing Bai, Yue Cao, Sirui Lin, Linshuoshuo Lyu, Haohao Meng, Yuwei Dai, Ding Ye, Weiyi Pan, Zhiyong Wang, Yingying Mao & Qianming Chen*

**Additional file 2: Supplementary Figures**

**Figure of Contents**

[***Fig. S1. Forest plot of the results after removing potential pleiotropic SNPs. 2***](#_Toc133445277)

[***Fig. S2. Results of leave-one-out sensitivity analysis. 3***](#_Toc133445278)

[***Fig. S3. Forest plot of the results in reverse MR for the causal effects of periodontitis on genetically predicted gut microbiota composition. 4***](#_Toc133445279)


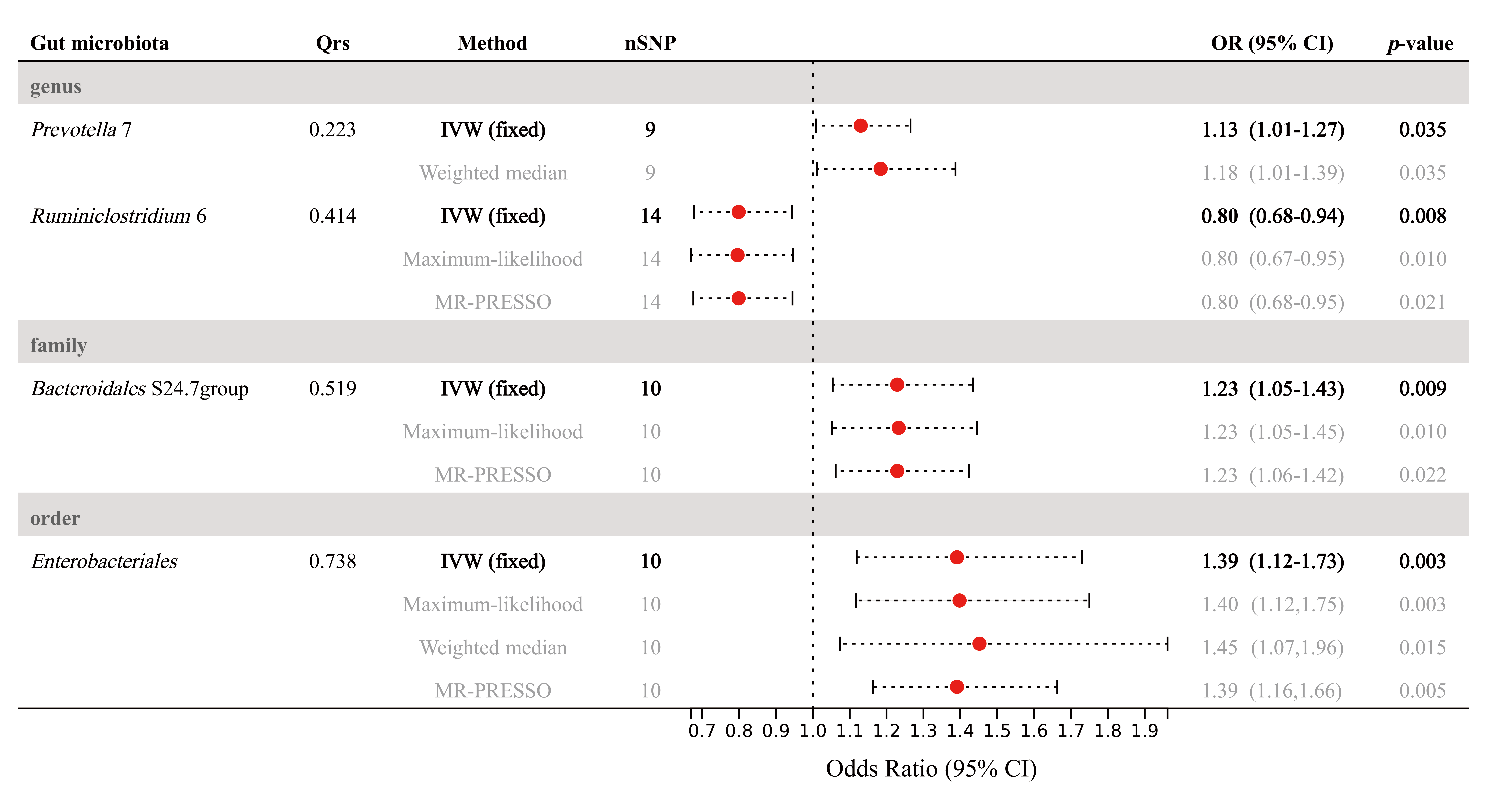


**Fig. S1** Forest plot of the results after removing potential pleiotropic SNPs. *Abbreviations*: *CI,* confidence interval; *IVW*, inverse-variance weighted; *MR*, Mendelian randomization; *MR-PRESSO*, Mendelian Randomization Pleiotropy RESidual Sum and Outlier; *OR*, odds ratio; *SNP*, single nucleotide polymorphism.


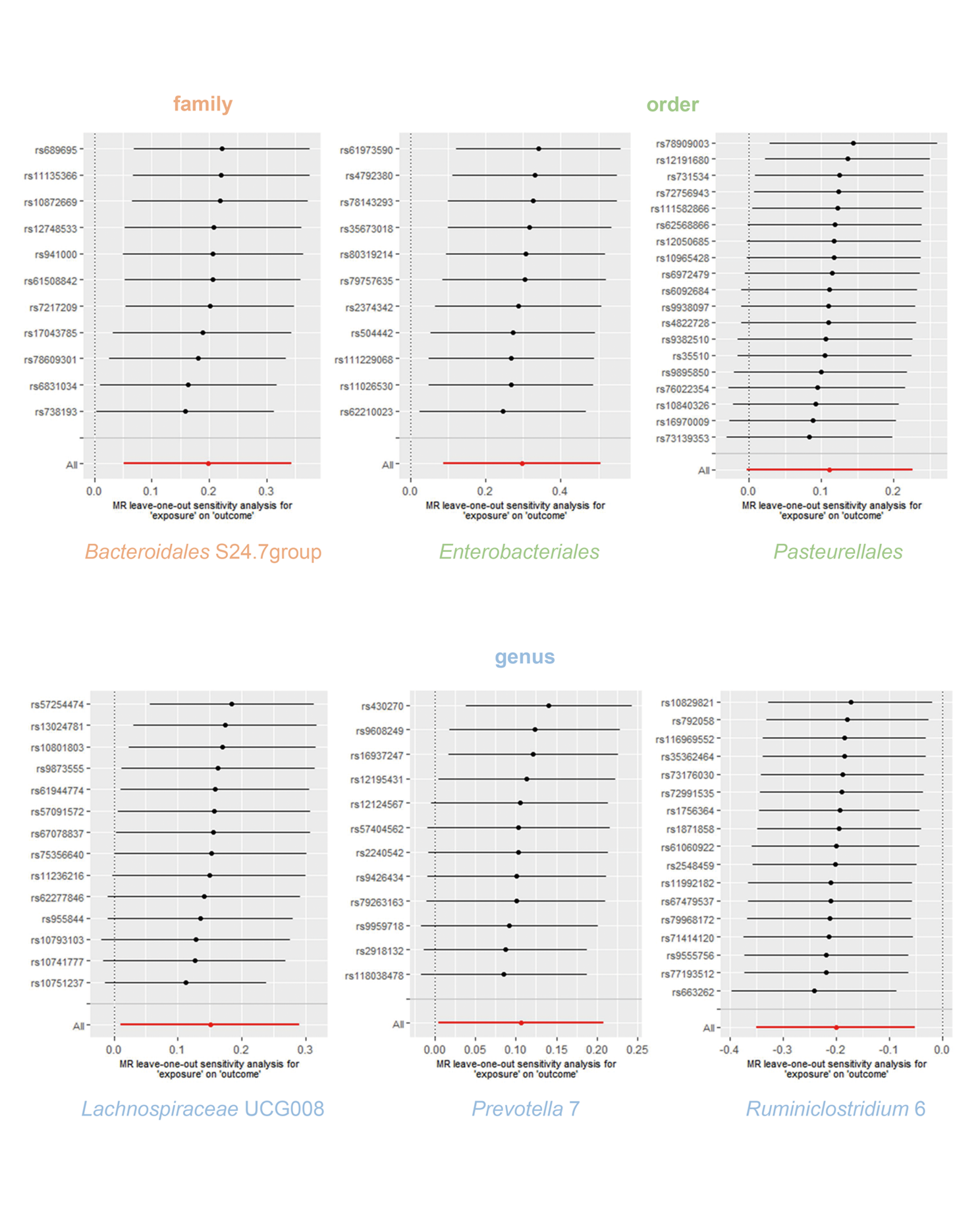


**Fig. S2** Results of leave-one-out sensitivity analysis. The effect estimates are re-calculated after removal of each SNP to identify if a single SNP is driving the association. *Abbreviations: MR*, Mendelian randomization; *SNP*, single nucleotide polymorphism.


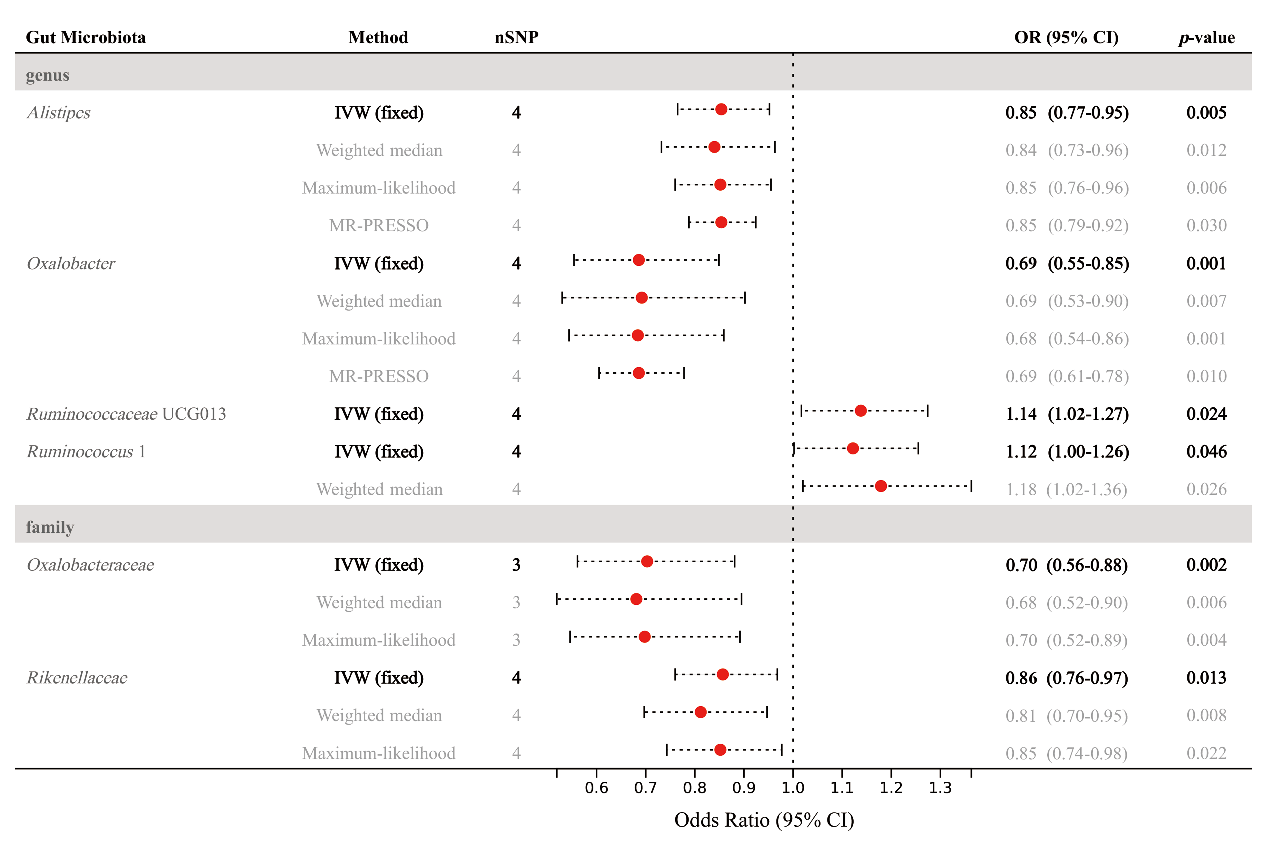


**Fig. S3** Forest plot of the results in reverse MR for the causal effects of periodontitis on genetically predicted gut microbiota composition. *Abbreviations*: *CI*, confidence interval; *IVW*, inverse-variance weighted; *MR*, Mendelian randomization; *MR-PRESSO*, Mendelian Randomization Pleiotropy RESidual Sum and Outlier; *OR*, odds ratio; *SNP*, single nucleotide polymorphism.
